# Long-term neuropsychologic outcome of pre-emptive mTOR inhibitor treatment in children with tuberous sclerosis complex (TSC) under 4 months of age (PROTECT), a two-arm, randomized, observer-blind, controlled phase IIb national multicentre clinical trial: study protocol

**DOI:** 10.1186/s13023-024-03495-1

**Published:** 2025-01-06

**Authors:** Jan H. Driedger, Julian Schröter, PROTECT-Study Group, Christoph Hertzberg, Bernhard Weschke, Angela M. Kaindl, Thomas Lücke, Charlotte Thiels, Kerstin Alexandra Klotz, Walid Fazeli, Kevin Rostásy, Lucia Wiethoff-Ubrig, Olaf Kaiser, Regina Trollmann, Dilbar Mammadova, Susanne Schubert-Bast, Alexia Bach, Matthias Eckenweiler, Jan Schönberger, Kyriakos Martakis, Andreas Hahn, Knut Brockmann, Steffi Dreha-Kulaczewski, Deike Weiss, Jonas Denecke, Hiltrud Muhle, Maria Arelin, Andreas Merkenschlager, Ingo Borggräfe, Timo Roser, Daniel Ebrahimi-Fakhari, Barbara Fiedler, Jan-Ulrich Schlump, Ilka Köster, Christoph Korenke, Michael Alber, Susanne Ruf, Martha Feucht, Theresa Scholl, Steffen Syrbe, Afshin Saffari

**Affiliations:** Division of Pediatric Epileptology, Department of Pediatrics I, Medical Faculty of Heidelberg, Heidelberg University, Heidelberg, Germany

**Keywords:** Tuberous sclerosis complex, Precision medicine, mTOR inhibitor therapy, Multicenter clinical trial

## Abstract

**Background:**

Tuberous sclerosis complex (TSC) is an autosomal dominant genetic disorder affecting multiple organ systems, with a prevalence of 1:6,760–1:13,520 live births in Germany. On the molecular level, TSC is caused by heterozygous loss-of-function variants in either of the genes *TSC1* or *TSC2*, encoding the Tuberin-Hamartin complex, which acts as a critical upstream suppressor of the mammalian target of rapamycin (mTOR), a key signaling pathway controlling cellular growth and metabolism. Despite the therapeutic success of mTOR inhibition in treating TSC-associated manifestations, studies with mTOR inhibitors in children with TSC above two years of age have failed to demonstrate beneficial effects on disease-related neuropsychological deficits. It has thus been hypothesized, that the critical time window for mTOR inhibitors may lie in early infancy, before TSC-related symptoms such as early-onset epilepsy and infantile spasms as sign of disruptive brain maturation occur. No controlled prospective clinical trials have evaluated the effect of pre-symptomatic mTOR inhibitor therapy on neuropsychological manifestations in TSC patients under two years of age.

**Methods:**

This two-arm, randomized, observer-blind, phase IIb national multicenter clinical trial aims at investigating the long-term neuropsychologic outcomes of pre-emptive mTOR inhibitor treatment in children diagnosed with TSC under four months of age. Sixty participants will be allocated to the trial with a 1:1 randomization ratio. The primary endpoint will be the neuropsychological outcome assessed by the cognitive scale of the Bayley Scales of Infant and Toddler Development III at 24 months of age compared to Standard of Care. Secondary endpoints include neuropsychologic outcomes at 12 months of age, seizure frequency, cardiac and cerebral tumor load, and safety assessments. Inclusion criteria are a definite TSC diagnosis and an age below four months at enrolment. The investigational medicinal product is sirolimus (Rapamune®), administered orally based on body surface area and surveilled by pharmacokinetic measurements, starting within the first four months of life and continuing until the second birthday.

**Conclusion:**

This study addresses a critical gap in understanding the impact of pre-emptive mTOR inhibitor therapy on neuropsychologic outcomes in young TSC patients, aiming to improve overall patient outcomes and quality of life.

EUCT number: 2022–502332-39–00, Registered 22/06/2023, https://euclinicaltrials.eu/search-for-clinical-trials/?lang=en&EUCT=2022-502332-39-00

**Supplementary Information:**

The online version contains supplementary material available at 10.1186/s13023-024-03495-1.

## Background and clinical trial rationale

Tuberous sclerosis complex (TSC) is an autosomal dominant neurodevelopmental disorder with varying penetrance and expressivity. It affects approximately 1:6,760 – 1:13,520 live births in Germany [1], presenting with variable disease severity ranging from mild skin lesions and minimal neurologic signs to severe neurodevelopmental deficits, including refractive epilepsy, autism spectrum disorder (ASD) and profound intellectual disability (ID). Mortality is elevated, especially in those with ID, epilepsy, and ASD [2, 3]. On the molecular level, TSC results from heterozygous loss-of-function variants in *TSC1* or *TSC2,* encoding the Tuberin-Hamartin complex. Together, they negatively regulate mTOR Complex 1 (mTORC1). Haploinsufficiency of either of these genes leads to a constitutive activation of the mammalian target of rapamycin (mTOR) pathway, resulting in fundamental alterations in neuronal network properties and an imbalance in neuronal excitation and inhibition, acting as a common pathway toward epilepsy, ID and ASD [4, 5]. Sirolimus binds to the intracellular protein FKBP12 to form a complex that directly inhibits mTORC1 activity [6]. By inhibiting mTORC1, sirolimus counteracts the hyperactivation caused by pathogenic *TSC1/TSC2* variants [7]. Mammalian target of rapamycin (mTOR)- inhibitors have shown efficacy in treating TSC-related manifestations, but their impact on neuropsychological outcomes remains unclear [8–11]. Pre-emptive initiation of mTOR inhibitor treatment may mitigate TSC-related neuropsychologic deficits, but controlled clinical trials in infants under 2 years are lacking [12, 13]. Neuropsychologic deficits in TSC significantly impact quality of life and lifespan [14]. Furthermore, current treatment regimens including anti-seizure medications have shown limited efficacy in improving neurodevelopmental outcomes [15, 16]. In animal models, effects have been demonstrated that suggest a reversibility of neuropsychological symptoms through mTOR inhibitor treatment [17–19]. Based on the mechanism of action, its influence on other manifestations, and preliminary evidence from animal models, it can be hypothesized that early initiation of mTOR inhibitors may offer a potential preventive strategy.

Due to limited safety data in neonates and young infants, mTOR inhibitors are often prescribed off-label. Recent studies, found that adverse events were generally mild to moderate, and no life-threatening events were reported [13, 20, 21]. Common adverse events included anemia, thrombocytosis, hyperlipidemia, and infections. Information on the long-term effects of sirolimus on growth and organ development in young infants is limited. Studies, including a 5-year follow-up of children with hyperinsulinemic hypoglycemia and data from kidney and liver transplant patients, suggest a generally manageable safety profile with common side effects such as infections and anemia, but no significant impact on growth or maturation [22–26].

In this study, we primarily focus on neuropsychological development following pre-emptive mTOR inhibitor treatment. The high risk for cognitive impairment in TSC and robust data on the positive effects of mTOR inhibition on multiple TSC manifestations, along with a manageable risk profile, justify this clinical trial.

## Study design and methods

**Fig. 1:**
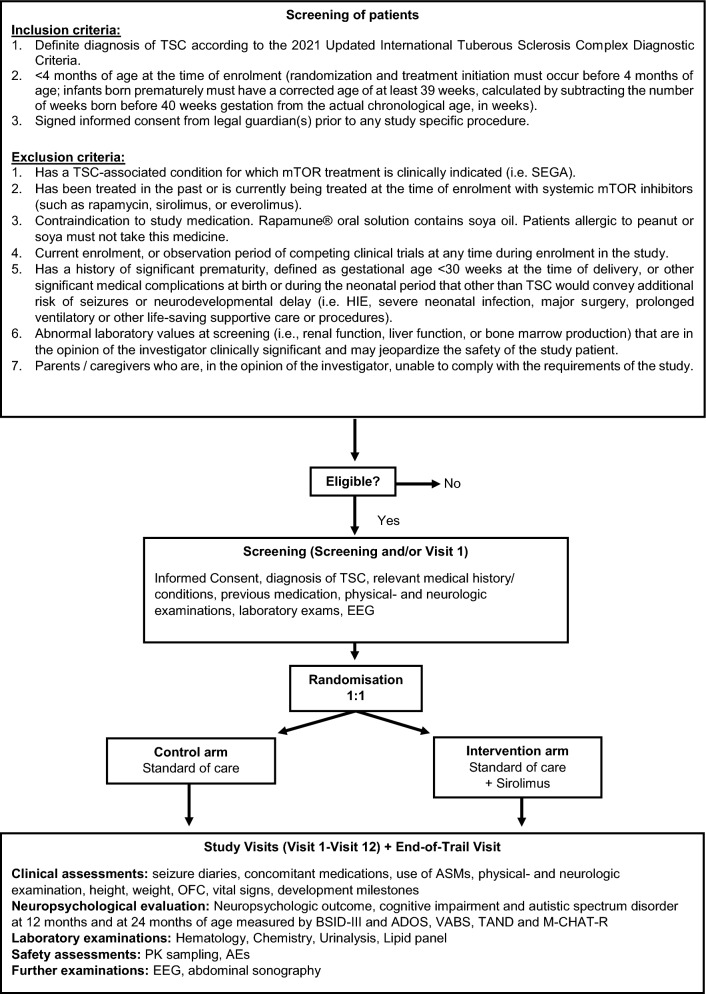
Schematic diagram of trial AE: adverse event, ADOS: autism diagnostic observation schedule, ASM: anti-seizure medication, BSID-III: bayley scales of infant and toddler development III, HIE: hypoxic ischemic encephalopathy, M-CHAT-R: modified checklist for autism in toddlers revised, OFC: occipitofrontal head circumference, PK: pharmacokinetic, SEGA: subependymal giant cell astrocytoma, TAND: tuberous sclerosis complex-associated neuropsychiatric disorders, VABS: vineland adaptive behavior scales

The clinical trial is designed as a two-armed, randomized, observer-blind, controlled phase IIb clinical trial for children < 4 months of age at inclusion. Neuropsychological testing for the primary and secondary endpoints will be conducted by a blinded external observer who is not involved in the treatment and has no access to any information regarding the treatment of study participants. The treatment group will receive daily oral mTOR inhibitor (sirolimus) therapy in addition to standard of care (SOC). Sirolimus will be continued until the 2nd birthday when neuropsychologic testing will be done. The control group will receive SOC treatment only. Patients randomized to the treatment group will be followed closely with mandatory study visits at screening, start of treatment (V1), titration visits V2-V4 and at an age of 3, 6, 9, 12, 15, 18, 21 and 24 months for safety measures, to adjust sirolimus dosages following pharmacokinetic (PK) testing according to a prespecified algorithm and to ensure compliance (Table 1). In case neonates receive their first dose of the investigational medicinal product (IMP) under the age of 4 weeks (corrected gestational age < 4 weeks of life), caregivers will have up to two additional telephone calls by the study personnel up to the end of the 4th week of life. An end-of-trial visit at 25 months of age will monitor safety parameters after discontinuation of treatment. Following the guidance provided by the German Federal Institute for Drugs and Medical Devices (BfArM) for conducting this clinical trial involving neonates and young infants in compliance with Good Clinical Practice (GCP), no placebo will be administered to the control group. This directive was aimed at reducing the study-associated burden by avoiding daily intake of sham medication and reducing the number of required laboratory examinations. Laboratory examinations in the control group ensures adequate monitoring for laboratory abnormalities unrelated to the study intervention. Therapy of TSC manifestations according to SOC in both groups will not be affected by the trial. Seizure diaries, use of anti-seizure medications and concomitant medications, adverse events, vital signs (blood pressure, temperature, heart rate), height, body weight, occipital frontal circumference, physical and neurological exam, and developmental milestones will be assessed at every study visit. Neuropsychologic testing will be performed at 12 and 24 months of age (Fig. 1).

**Table 1:** Schedule of enrolment, interventions and assessment

Visit Title	Screening	Treatment + SOC / SOC												Follow-Up	
Visit No	Screening^1^	V1^§^	V2	V3	V4 ^2^	V5 ^3^	V6	V7	V8	V9	V10	V11	V12	End of trial Visit (V13)	V14
Titration phase, then months of life	− 14 to 0 days	Treatment Start	Titration in 14-days intervals^#^			m3	m6	m9	m12	m15	m18	m21	m24	m25	m60 ^optional^
Time Slot [days]	− 14 to 0 days	0	± 4			± 7	± 7	± 7	± 14	± 7	± 7	± 7	± 14	± 7	± 30
Informed Consent	X														
Diagnosis of TSC	X														
Inclusion/ exclusion criteria	X														
Relevant medical history/current conditions	X														
Randomization		X													
Previous ASM therapy	X														
Concomitant ASM therapy	X	X	T	T	T	T/C	T/C	T/C	T/C	T/C	T/C	T/C	T/C	T/C	
Other concomitant medications	X	X	T	T	T	T/C	T/C	T/C	T/C	T/C	T/C	T/C	T/C	T/C	
Vital signs ^4^	X	X	T	T	T	T/C	T/C	T/C	T/C	T/C	T/C	T/C	T/C	T/C	
Height/weight	X	X	T	T	T	T/C	T/C	T/C	T/C	T/C	T/C	T/C	T/C	T/C	
/OFC															
Physical examination	X	X	T	T	T	T/C	T/C	T/C	T/C	T/C	T/C	T/C	T/C	T/C	
Neurologic examination	X	X	T	T	T	T/C	T/C	T/C	T/C	T/C	T/C	T/C	T/C	T/C	
Seizure diary ^5^	X	X	T	T	T	T/C	T/C	T/C	T/C	T/C	T/C	T/C	T/C	T/C	
Developmental milestones ^6^						T/C	T/C	T/C	T/C		T/C		T/C		
Neuropsychologic evaluation															
BSID-III									T/C				T/C		
ADOS									T/C				T/C		
VABS									T/C				T/C		T/C*
TAND									T/C				T/C		T/C*
M-CHAT-R													T/C		
SCQ															T/C
QOLCE ^7^															T/C
Laboratory examinations															
Hematology ^8^	X		T	T	T	T	T/C	T	T/C	T	T	T	T/C	T	
Chemistry ^9^	X		T	T	T	T	T/C	T	T/C	T	T	T	T/C	T	
Lipid panel ^10^	X						T/C		T/C		T		T/C		
Urinalysis ^11^	X								T/C		T		T/C		
Vaccination titer ^optional^									T/C				T/C		
RNA ^optional^	X												T/C		
DNA ^optional^	X												T/C		
Safety assessments															
Adverse events ^12^	X	X	T	T	T	T/C	T/C	T/C	T/C	T/C	T/C	T/C	T/C	T/C	
Individual risk–benefit evaluations							T		T		T				
Sirolimus PK sampling			T	T	T	T	T	T	T	T	T	T	T		
ASM PK sampling^13^	X		T				T		T		T		T		
Therapy (dosing)^14^		T	T	T	T	T	T	T	T	T	T	T	T		
Abdominal sonography^15^	X												T/C		
EEG	X			T			T/C		T/C		T/C		T/C		
cMRI, Echo, ECG ^16, 17^	T/C														

X: all participants, C: control group, T: treatment group, SOC: standard of care, m: months, ADOS: autism diagnostic observation schedule, ASM: anti-seizure medication, BSID: bayley scales of infant and toddler development, M-CHAT-R: modified checklist for autism in toddlers revised, OFC: occipitofrontal head circumference, PK: pharmacokinetic, QOLCE: quality of life in childhood epilepsy questionnaire, SCQ: social communication questionnaire, TAND: tuberous sclerosis complex-associated neuropsychiatric disorders, VABS: vineland adaptive behavior scales, QOLCE: quality of life in childhood epilepsy

^#^14-days intervals beginning on day 0

^§^Neonates who receive their first dose of IMP under the age of 4 weeks (corrected gestational age < 4 weeks of life): Caregivers will have up to two additional telephone calls by the study personnel up to the end of the 4th week of life. These calls will take place in those weeks (± 2 days) where no titration visit takes place. Caregivers will be asked about the condition of their child. If it turns out that any sign occurs that requires an on-site visit, the study staff will act accordingly. At the discretion of the investigator, a blood sample could be taken for a safety laboratory which includes the blood alcohol concentration (BAC) and surrogate parameters for potential propylene glycol (PG) intoxication including electrolytes, osmolality, creatinine, urea, pH, lactate, bicarbonate, anion gap, and the calculated osmol gap

^1^Screening examinations can be conducted up to 14 days before study entry including the day of Visit one (V1). Provided all inclusion criteria are met and the informed consent form (ICF) has been signed, Screening Visit and Visit one (V1) may take place at the same day. All procedures for the screening visit must be performed prior to randomization/administration of first dose

^2^Can be omitted, if target concentration range of IMP was correct without adjustment in V2 and V3

^3^Can be omitted, if the last titration visit was ≤ 1 month ago

^4^Heart rate, blood pressure, temperature

^5^Seizure diaries distributed and /or collected and evaluated

^6^For details see Supporting Information S1 chapter 6.8

^7^For details see Supporting Information S1 chapter 2.2.2

^8^Complete blood count with differential blood count

^9^For details see Supporting Information S1 chapter 6.8

^10^For details see Supporting Information S1 chapter 6.8

^11^For details see Supporting Information S1 chapter 6.8

^12^Graded by the Common Terminology Criteria of Adverse Events (CTCAE, Version 5.0 or most recent version)

^13^ASM PK sampling if applicable

^14^Regarding sirolimus dose adjustments, an additional pre-dose PK blood sample should be collected after 14 days, which during the beginning of the trial should coincide with the next regular study visit and after that during an additional appointment

^15^Allowed to be 8 weeks old at day 0

^16^Examinations are carried out as part of the standard of care

^17^Last cMRI/ECG/Echo carried out have to be entered in eCRF

^optional^ optional intervention within sub-trials, if specific informed consent is given

### Inclusion criteria

Subjects meeting all of the following criteria will be considered for admission to the clinical trial:

1. Definite diagnosis of TSC according to the 2021 Updated International Tuberous Sclerosis Complex Diagnostic Criteria [27].

2. <4 months of age at the time of enrolment (randomization and treatment initiation must occur before 4 months of age; infants born prematurely must have a corrected age of at least 39 weeks of gestation).

3. Signed informed consent from legal guardian(s) prior to any study specific procedure.

### Exclusion criteria

Subjects presenting with any of the following criteria will not be included in the clinical trial:

1. Tuberous sclerosis complex -associated condition for which mTOR treatment is clinically indicated (i.e. subependymal giant cell astrocytoma (SEGA)).

2. Previous or current treatment at the time of enrolment with systemic mTOR inhibitors (such as rapamycin, sirolimus, or everolimus).

3. Contraindication to study medication. Rapamune® oral solution contains soya oil. Patients allergic to peanut or soya must not take this medicine.

4. Current enrolment, or observation period of competing clinical trials at any time during enrolment in the study.

5. History of significant prematurity, defined as gestational age <30 weeks at the time of delivery, or other significant medical complications at birth or during the neonatal period that other than TSC would convey additional risks of seizures or neurodevelopmental delay (i.e. HIE, severe neonatal infection, major surgery, prolonged ventilatory or other life-saving supportive care or procedures).

6. Abnormal laboratory values at screening (i.e., renal, hepatic and/or bone marrow dysfunction) that are clinically significant and may jeopardize the safety of the study patient, in the opinion of the investigator.

7. Parents / caregivers who are, in the opinion of the investigator, unable to comply with the requirements of the study.

### Randomization

Within 14 days prior to the first study visit (V1) and the time of randomization, all required elements of the screening visit will be fully completed and documented, including informed consent, confirmation of the diagnosis of TSC, and review of inclusion and exclusion criteria. Vital signs, physical examination, baseline electroencephalography (EEG), abdominal sonography, and laboratory results are documented and entered into the electronic case report form (eCRF). After internal validation, randomization is secured via the eCRF at V1, and treatment starts on the same day with the calculated starting dose. The randomization uses a 1:1 ratio for interventional and control arms with block randomization to minimize imbalance. While center effects are not expected due to standardized therapeutic management, age at inclusion might introduce bias, so randomization is stratified by age groups (0 to < 2 months vs. 2 to < 4 months).

### Blinding

Neuropsychologic testing and scoring will be performed by a blinded external observer, not involved in other study procedures. During neuropsychologic testing no other study related activities (e.g. blood sampling) will be performed. Caregivers will be instructed not to disclose their study group.

Electroencephalography, cranial magnetic resonance imaging (cMRI) and abdominal sonography recordings will be centrally analyzed by independent blinded raters.

### Trial visits and investigations/assessments

The clinical trial period for an individual subject will consist of screening examinations, 21–24 months of treatment period (depending on the age at inclusion from 0 to < 4 months of age), followed by a one-month safety follow-up after treatment period. Detailed investigations at each study visit are outlined in Table 1.

### Administration of investigational medicinal product

After randomization, the starting dose of the IMP in the treatment group will be determined based on the patient's age, height, and weight. The first dose will be administered at the trial center, and caregivers will be provided with the IMP and oral syringes for administration at home. Sirolimus (Rapamune^®^) will be administered twice daily, with specific dosing adjustments for neonates and infants. Caregivers will be thoroughly trained to administer the medication, and compliance will be verified by assessing the amount of the dose administered. Sirolimus trough levels will be measured at each study visit and dose adjustments will be made to achieve serum target levels of 5–10 ng/ml.

### Initial dosing and dose modifications

Sirolimus dosing recommendations vary among studies depending on age of subjects and indications. Sirolimus has a narrow therapeutic window with considerable inter-individual pharmacokinetic variabilities. Most studies report median absolute doses of 2–2.5 mg daily [28]. Almost all dosing recommendation for sirolimus in neonates and infants with indications other than TSC vary around 0.05–0.1 mg/kg or 0.5–1 mg/m^2^ in line with our dosing scheme. The traditional fixed-dose stratification or weight-based dosing designs only control for a small part of the variable age-dependent metabolization of sirolimus, frequently leading to drug exposures outside of the targeted range [29]. Developmental changes over time in neonates and infants have a major impact on the pharmacokinetic-pharmacodynamic (PK/PD) profile of mTOR inhibitors such as sirolimus (e.g. age-dependent change in CYP3A activity) [30]. Mizuno and colleagues developed an age-appropriate sirolimus starting dose regimen for neonates and infants based on developmental changes in drug elimination capacity using 316 concentration measurements from 25 patients aged 0–4 years (15 of which were less than 2 years old) and achieved target attainment of more than 75–95% across selected regimens [31].

Based on the presented evidence, and in agreement with international TSC experts, the study uses the model proposed by Mizuno et al. accounting for pharmacokinetic data of infants/neonates, body surface area (BSA) and uses a twice daily (BID) regimen, which was shown to reduce absolute doses [32] (Table 2). To reduce the occurrence of AEs we target trough levels of 5–10 ng/ml.

**Table 2:** Calculation of starting dose of IMP

Age group (months)	Daily Dose (mg/m^2^/day)
0–< 1	0.5
1–< 2	0.6
2–< 3	0.74
3–< 4	0.9

A dose adjustment scheme was established in collaboration with the study teams of the currently active trials in the US (ClinicalTrials.gov: NCT05104983) and Poland (ClinicalTrials.gov: NCT04987463) and is guided by previous experience. Depending on PK levels at each study visit doses will be modified following Table 3.

**Table 3:** Sirolimus dose adjustment scheme

Sirolimus trough levels (PK in ng/ml)	Required dose adjustment
PK < 5	Double all subsequent doses; additional telephone contact of trial site with caregiver(s) after 7 days +/- 2 days; repeat levels in 2 weeks
5 ≤ PK ≤ 10	No adjustment
10 < PK ≤ 15	If no clinically significant AE or laboratory abnormalities occur, no dose adjustments; repeat levels in 2 weeks
15 < PK ≤ 20	Reduce all subsequent doses to 75%; repeat levels in 2 weeks
PK > 20	Skip next dose, then reduce all subsequent doses to 50%; repeat levels in 2 weeks

### Endpoint measurement and statistical analysis

As primary endpoint, the BSID-III cognitive scale at 24 months of age will be analyzed using a two-sided Mann–Whitney-U-test (alpha = 0.05) stratified by age groups (0 to < 2 and 2 to < 4 months of age) comparing the treatment versus the control group. All randomized patients will be evaluated following the treatment policy strategy (ICH E9 R1). Following power calculations, 60 patients will be randomized and an improvement of 15 points in the BSID-III cognitive scale at the age of 24 months (e.g. a score of 75 in the control group and a score of 90 in the treatment group) will be considered relevant for reducing neuropsychological impairment, therefore lifting burden of disease and improving social participation and opportunities in school and professional life (for details on statistical methods see Supporting Information S1).

As secondary endpoint, the BSID-III cognitive scale also at the age of 12 months will be analyzed. In addition, the BSID-III cognitive scale, along with the Vineland Adaptive Behavior Scales 3 (VABS-3) and Autism Diagnostic Observation Schedule 2 (ADOS-2) total scales as well as selected respective subscales will be analysed descriptively by group and time point using standard descriptive measures for continuous variables. The Bayley-III and the ADOS-2 have been utilized in several studies focusing on Tuberous Sclerosis Complex (TSC) to assess neurodevelopmental outcomes and autism spectrum disorder (ASD) symptoms (e.g., EPISTOP Trial) [33, 34].

The Modified Checklist for Autism in Toddlers, Revised with Follow-Up (M-CHAT-R/F) and the TSC-Associated Neuropsychiatric Disorders Checklist (TAND-Checklist) will be analysed descriptively by group and time point using absolute and relative frequencies.

MRI characteristics, seizure characteristics, EEG characteristics, abdominal sonography characteristics, ECG and echocardiography will be analysed by group and time point (if applicable). All available cMRI data, conducted as part of standard of care, will be collected and analyzed. Exploratory analyses will focus on MRI characteristics, stratified by study group and time point. Correlations between early MRI findings and the primary and secondary neuropsychiatric endpoints will be examined. All safety parameters will be analysed. In addition, adverse events and special situations will be analysed by categories defined in the eCRF (number of events as well as number and percentage of patients with at least one event).

### Status and timeline of the study

The duration of the clinical trial for each subject is scheduled to span 21–25 months until 24 months of age, depending on the time of enrolment (0 to < 4 months of age) and a one-month follow-up.

The overall duration of the clinical trial is expected to be 82 months. Recruitment of subjects started in Q1 2024. The actual overall and/or recruitment duration may vary. An initial safety analysis after the titration phase (V4) of 5 patients in the treatment group will be done.

### Patient involvement

This trial was designed with early-on involvement of patients and patient organizations and is supported by the *German Tuberöse Sklerose Deutschland e.V. (TSD)* and the *Allianz Chronischer Seltener Erkrankungen e.V.“ (Achse e.V.)*.

## Discussion

This trial will provide information on benefits and safety of prophylactic mTOR inhibitor treatment in reducing the occurrence and severity of neuropsychologic impairment in TSC. Using a rational, mechanism-based therapeutic strategy, this trial could fundamentally transform the therapeutic management of TSC-related neuropsychiatric manifestations from a symptomatic to a targeted disease-modifying approach. In a disease such as TSC, early intervention may improve long-term performance in school, social and professional life and TSC could serve as a model for targeted, mechanism-driven approaches for related neurodevelopmental disorders with and without dysregulated mTOR signaling. Reducing neuropsychologic deficits can improve quality of life and participation, as well as reduce morbidity and mortality, hospitalization and the number of pharmacologic and non-pharmacologic therapies required for the treatment of associated long-term complications [14, 35].

The fact that TSC is, in the majority of cases, diagnosed prenatally based on the presence of cardiac rhabdomyomas detected on gestational ultrasounds or shortly after birth due to central nervous system involvement [1, 36], provides a unique opportunity for obstetricians, clinical geneticists, pediatricians, and pediatric neurologists to work together to provide affected families with adequate information and ensure early referral to a study center or patient organization. Early diagnosis of TSC and referral will enable study inclusion in time. Eventually, the study will provide data on the influence of postnatal treatment to improve neuropsychological outcome in a patient group at risk for developmental disorders.

## Supplementary Information

Additional file 1: Study protocol.

## Data Availability

The data from the PROTECT trial will be archived electronically at University Hospital Heidelberg. “heiDATA”, a professional data repository provided by the Heidelberg University Competence Centre for Research Data. Data will be made available, as far as legally possible. Data of individual participants will be made available after deidentification when reporting the final results of the trial (text, tables, figures, appendices, study protocol, statistical analysis plan, and analytic code). Data will be shared with researchers upon request directed to the principal coordinating investigator or data repository of Heidelberg University (heiDATA, https://heidata.uni-heidelberg.de).
