# Supplementary material for: Long-term neuropsychologic outcome of pre-emptive mTOR inhibitor treatment in children with tuberous sclerosis complex (TSC) under 4 months of age (PROTECT), a two-arm, randomized, observer-blind, controlled phase IIb national multicentre clinical trial: study protocol
Source: Orphanet J Rare Dis. 2025 Jan 6;20:2. doi: 10.1186/s13023-024-03495-1 (PMC11702139; doi:10.1186/s13023-024-03495-1)
Supplement: Supplementary file 1 — Additional file 1: Study protocol. [file 13023_2024_3495_MOESM1_ESM.pdf]

CLINICAL TRIAL PROTOCOL

PROTECT

2022-502332-39-00

Version 06; 01.10.2024

Long-term neuropsychologic outcome of pre-emptive mTOR inhibitor treatment in children with tuberous sclerosis complex (TSC) under 4 months of age (PROTECT)

Neuropsychologische Langzeit-Entwicklung von vorbeugend mit mTOR-Inhibitoren behandelten Kindern im Vergleich zur Standardbehandlung bei Kindern mit tuberöser Sklerose unter 4 Monaten (PROTECT)

Phase of clinical trial: Therapeutic exploratory (Phase IIb)

Coordinating Investigator: Prof. Dr. Steffen Syrbe

CONFIDENTIAL: This protocol contains confidential information and is intended solely for the guidance of the clinical investigation. This protocol may not be disclosed to parties not associated with the clinical investigation or used for any purpose without the prior written consent of the Coordinating Investigator.

PROTOCOL SIGNATURE PAGE

The present trial protocol was subject to critical review and has been approved in the current version by the persons undersigned. The information contained is consistent with:

- the current risk-benefit assessment of the investigational medicinal product,
- the moral, ethical and scientific principles governing clinical research as set out in the latest relevant version of Declaration of Helsinki, the principles of the guidelines of ICH Good Clinical Practices and the applicable legal and regulatory requirements.

The investigators will be supplied with details of any significant change of the benefit-risk-assessment of the trial.

It will be ensured that the first subject is enrolled only after all ethical and regulatory requirements are fulfilled. Written consent from all legal guardian(s) of subjects is received after detailed oral and written information and according to the requirements of applicable local law (Art. 28, 29 CTR, § 40b of AMG). According to §40b Section 6 No. 1 of AMG it will be confirmed that legal guardian(s) of all clinical trial subjects will be informed on the type of encoding their personal data (pseudonymization) and who receives or has access to such data. Legal guardian(s) of subjects who do not agree to this data encoding and transfer will not be enrolled into the trial. In this context it will be assured (according to Art. 56 of CTR) that all investigational sites comply with the local regulatory requirements for data protection.

Via current versions of the clinical trial protocol and the SmPC it will be ensured that all principal investigators are informed about the pharmacological-toxicological assessments and results regarding the benefits and risks of the clinical trial.

Date:	___________________	Signature:	___________________________
		Name (Print Name):	Univ.-Prof. Dr. Steffen Syrbe
		Function:	Sponsor Representative / Coordinating Investigator
Date:	___________________	Signature:	___________________________
		Name (Print Name):	[redacted]
		Function:	Biometrician
Date:	___________________	Signature:	___________________________
		Name (Print Name):	[redacted]
		Function:	Author, Medical Coordinator
Date:	___________________	Signature:	___________________________
		Name (Print Name):	[redacted]
		Function:	Author, Medical Coordinator

INVESTIGATOR SIGNATURE PAGE

I have read the above-mentioned trial protocol and confirm that it contains all information to conduct the clinical trial. I pledge to conduct the clinical trial according to the protocol.

I will enroll the first subject only after all ethical and regulatory requirements are fulfilled. I will obtain written consent for trial participation from all legal guardian(s) of subjects after detailed oral and written information according to the requirements of applicable local law and regulations governing the conduct of clinical studies (e.g. Art. 28, 29 of CTR). According to local law and regulations governing the conduct of clinical studies I declare that all legal guardian(s) of clinical trial subjects will be informed on the type of encoding their personal data (pseudonymization) and who receives or has access to their data. Legal guardian(s) of subjects who do not agree to this data encoding and transfer will not be enrolled into the trial. In this context I confirm (according to Art. 56 of CTR, if applicable) that my investigational site complies with all local regulatory requirements for data protection.

I know the requirements for accurate notification of serious adverse events and serious breaches, and I pledge to document and notify such events as specified in the protocol.

I declare that I am informed about the pharmacological-toxicological assessments and results regarding the benefits and risks of the clinical trial by reading the description in the clinical trial protocol and in the current version of SmPC. I ensure that all investigators / relevant staff at my site will be informed of these results and possible new risks that are forwarded by the sponsor later on (e.g. via new version of the SmPC).

I confirm and will ensure that all staff will be adequately trained to guaranty compliance to the trial protocol incl. subsequent amendments, the trial procedures and trial specific duties and tasks. I will maintain a list specifying the tasks delegated to each team member.

I will retain all trial-related documents and source data as described. I will provide a Curriculum Vitae (CV) before trial start. I agree that the CV and Financial Disclosure (FD) may be uploaded in CTIS, if applicable.

Date: ____________________

Signature: ____________________________

Name (Print Name): ____________________________

Function: Principal Investigator (PI)

Trial Site (Address):

____

____

____

INVOLVED PARTIES

Sponsor:

Heidelberg University Hospital

Represented in law by its Commercial Managing Director [redacted]

[redacted]

On behalf of Heidelberg University, Medical Faculty Heidelberg

Coordinating Investigator

University Hospital Heidelberg

Klinik für Kinderheilkunde I

Sektion für pädiatrische Epileptologie

Univ.-Prof. Dr. med. Steffen Syrbe

Im Neuenheimer Feld 430

D-69120 Heidelberg

[redacted]

Blinded centralised EEG assessment

University Hospital Heidelberg, Klinik für Kinderheilkunde I

Sektion Pädiatrische Epileptologie

Im Neuenheimer Feld 430

D-69120 Heidelberg

BIOBANK: DNA/RNA (accompanying research only)

Institut für Humangenetik, Universitätsklinikum Leipzig

[redacted]

Project Management

Koordinierungszentrum für Klinische Studien (KKS), University Hospital Heidelberg

Berliner Str. 10

D-69120 Heidelberg

[redacted]

Statistics

Koordinierungszentrum für Klinische Studien (KKS), University Hospital Heidelberg

Berliner Str. 10

[redacted]

Data Management

Koordinierungszentrum für Klinische Studien (KKS), University Hospital Heidelberg

Berliner Str. 10

D-69120 Heidelberg

[redacted]

Pharmacovigilance

Koordinierungszentrum für Klinische Studien (KKS), University Hospital Heidelberg

Berliner Str. 10

D-69120 Heidelberg

[redacted]

Monitoring

Koordinierungszentrum für Klinische Studien (KKS), University Hospital Heidelberg

Berliner Str. 10

D-69120 Heidelberg

[redacted]

Manufacturer of IMP

Pfizer Pharma GmbH

Linkstraße 10

D-10785 Berlin

[redacted]

Pharmacy

Pharmacy, University Hospital Heidelberg

Herstellbetrieb des Universitätsklinikum Heidelberg

Im Neuenheimer Feld 670

D-69120 Heidelberg

[redacted]

Data Safety Monitoring Board (DSMB)

[redacted]

[redacted]

[redacted]

Scientific Advisory Board (SAB)

[redacted]

[redacted]

[redacted]

[redacted]

[redacted]

PARTICIPATING SITES

All participating sites are listed in a separate document.

Table of Contents

PROTOCOL SIGNATURE PAGE 2
INVESTIGATOR SIGNATURE PAGE 3
INVOLVED PARTIES 4
TABLE OF CONTENTS 8
LIST OF TABLES 12
LIST OF FIGURES 12
PROTOCOL SYNOPSIS 13
SCHEDULE OF ASSESSMENTS 16
ABBREVIATIONS 19
1. INTRODUCTION AND RATIONALE 24
1.1 Scientific Background 24
1.2 Clinical Trial Rationale 24
1.3 Risk Benefit Assessment 25
1.3.1 Study-associated burdens and procedures 26
1.3.2 Clinical impact 27
1.3.3 Patient benefit 27
1.3.4 Inclusion of minors 27
1.3.5 Socioeconomic impact 28
1.3.6 Rational for study medication 29
1.3.7 Safety of sirolimus in neonates and young infants 30
1.3.7.1 Systematic research for evidence synthesis 30
1.3.8 Long-term tolerability of sirolimus in neonates and young infants 32
1.3.9 COVID-19 related risks 33
1.3.10 Individual risk-benefit evaluations 33
2. CLINICAL TRIAL OBJECTIVES AND ENDPOINTS 35
2.1 Primary Objective and Primary Endpoint 35
2.2 Secondary Objectives and Endpoints 35
2.2.1 Main Trial 35
2.2.2 Accompanying Research 36
3. CLINICAL TRIAL DESIGN 38
3.1 Overall Trial Design 38
3.2 Overall Duration of the Trial 40
3.3 Duration of Trial Participation for each Subject 40
3.4 Interim Analysis 40
3.5 Determination of End of Trial (all subjects) 40
3.6 Patient Involvement 40
4. SUBJECT SELECTION 42
4.1 Number of Subjects and Sites 42
4.2 General Criteria for Subject Selection 42
4.3 Inclusion Criteria 43
4.4 Exclusion Criteria 43
4.5 Lifestyle Considerations 43
4.6 Screening and Randomization 44
4.6.1 Identification Numbers 44
4.6.2 Screening Failures 44
5. TREATMENT 45
5.1 Measures to minimize Bias 45
5.1.1 Randomization 45
5.1.2 Blinding 45
5.1.3 Other 46
5.2 Investigational Medicinal Product (IMP) 46
5.2.1 Approval and Indication of IMP 46
5.2.2 Physical, Chemical, and Pharmaceutical Properties of IMP 47
5.2.2.1 Molecular Structure and Chemical Name 47

5.2.2.2 Physical and Chemical Properties ... 47
5.2.2.3 Nonclinical Pharmacology ... 48
5.2.2.4 Nonclinical Pharmacokinetics and Product Metabolism ... 48
5.2.2.5 Clinical Pharmacokinetics and Product Metabolism ... 48
5.2.2.6 Toxicology ... 49
5.2.2.7 Excipients with known effect ... 50
5.2.3 IMP Supplies ... 51
5.2.4 IMP Packaging and Labelling ... 51
5.2.5 IMP Storage ... 51
5.2.6 IMP Accountability ... 52
5.3 Auxiliary Medicinal Product(s) (AxMPs) ... 53
5.3.1 AxMP Supplies/Labelling/Storage ... 55
5.4 Administration ... 55
5.4.1 Preparation of Medication ... 57
5.5 Dosing ... 57
5.5.1 Maximum duration of treatment ... 59
5.5.2 Maximum dose allowed ... 59
5.5.3 Dose modifications and interruptions ... 59
5.5.4 Management of specific toxicity ... 62
5.5.4.1 Management of infections ... 62
5.5.4.2 Management of skin toxicity ... 62
5.5.4.3 Management of stomatitis / oral mucositis / mouth ulcers ... 62
5.5.4.4 Management of diarrhea ... 62
5.5.4.5 Management of non-infectious pneumonitis ... 62
5.5.5 Subject Compliance ... 63
5.6 Concomitant Medication and Therapy ... 63
5.6.1 Interaction with other medicinal products ... 64
5.6.2 Permitted Medication and Therapies ... 67
5.6.3 Prohibited or restricted Medication and Therapies ... 67
5.6.4 Rescue Medication/Treatment ... 67
6. TRIAL VISITS AND INVESTIGATIONS/ASSESSMENTS ... 68
6.1 Definition of standard of care ... 68
6.2 Screening Visit ... 69
6.3 Titration Visits ... 69
6.4 Safety Lab Visits ... 69
6.5 Treatment Visits ... 70
6.6 End of Trial Visit ... 71
6.7 Planned Treatment after End of Trial ... 71
6.8 Assessments ... 71
7. DISCONTINUATION AND EARLY TERMINATION ... 80
7.1 Temporary Discontinuation from Treatment ... 80
7.2 Permanent Discontinuation of Treatment ... 80
7.3 Discontinuation of Trial ... 81
7.3.1 Withdrawal of Consent ... 81
7.3.2 Lost to Follow-up ... 81
7.4 Criteria for Discontinuation of Dose Escalation ... 81
7.5 Temporary Halt ... 82
7.6 Early Termination of the Clinical Trial ... 82
7.7 Premature Close-out of a Site ... 82
8. ADVERSE EVENTS ... 83
8.1 Definitions ... 83
8.1.1 Adverse event ... 83
8.1.2 Serious Adverse Event ... 84
8.1.3 Serious Adverse Reaction ... 84
8.1.4 Adverse Events of Special Interest (AESI) ... 84
8.2 Expectedness ... 84

8.3 Suspected Unexpected Serious Adverse Reaction (SUSAR) ... 85
8.4 Characteristics of Adverse Events ... 85
8.4.1 Grading of AEs ... 85
8.4.2 Causal Relationship ... 86
8.4.3 Outcome of AEs ... 86
8.4.4 Action taken with the IMP ... 86
8.4.5 Countermeasures ... 87
8.5 Special Situations Reporting ... 87
8.6 Period of Observation and Documentation ... 88
8.6.1 Adverse Events (AEs) ... 88
8.6.2 Special Situations ... 88
8.7 Investigator's Pharmacovigilance related reporting Obligations ... 88
8.7.1 Reporting of Serious Adverse Events, Adverse Events of Special Interest and Special Situations ... 88
8.7.2 Reporting of Pregnancies ... 89
8.8 Sponsor's Pharmacovigilance related Obligations ... 89
8.8.1 Sponsor's Assessment (Second Assessment) ... 89
8.8.2 Expedited Reporting of SUSARs ... 89
8.8.4 Annual Safety Report ... 90
9. STATISTICAL PROCEDURES ... 91
9.1 Definition of Clinical Trial Population to be Analyzed ... 91
9.2 Analysis Variables ... 91
9.3 General Considerations ... 91
9.4 Primary Analysis ... 91
9.5 Secondary Analyses ... 92
9.5.1 Main Trial Analyses ... 92
9.5.2 Accompanying Research Analyses ... 92
9.6 Interim Analyses ... 93
9.7 Sensitivity Analyses ... 93
9.8 Subgroup Analyses ... 93
9.9 Sample Size/Power Calculation ... 93
10. DATA MANAGEMENT ... 95
10.1 Data Collection and Handling ... 95
10.2 Data Cleaning and Quality Checks ... 95
11. ARCHIVING AND STORAGE ... 96
11.1 Essential Documents and Source Data ... 96
11.2 Collection, Storage and future Use of Biological Samples and Corresponding Data only in Accompanying Research ... 96
12. REGULATORY, ETHICAL AND TRIAL OVERSIGHT CONSIDERATIONS ... 97
12.1 Compliance Statement ... 97
12.2 Data Protection and Subject Privacy ... 97
12.3 Trial Approval ... 98
12.4 Subject Information and Informed Consent ... 98
12.4.1 General Provisions ... 98
12.4.2 Special Provision for Incapacitated Subjects ... 98
12.5 Trial Committee Structure ... 98
12.5.1 Data Safety Monitoring Board (DSMB) ... 99
12.5.2 Steering Committee (SC) ... 99
12.5.3 Scientific Advisory Board (SAB) ... 99
12.6 Insurance ... 99
13. QUALITY CONTROL AND QUALITY ASSURANCE ... 100
13.1 Quality Assurance ... 100
13.2 Monitoring ... 100
13.3 Source Documents ... 100
13.3.1 Direct Access to Source Documents ... 100
13.4 Audits and Inspections ... 101

13.5 Serious Breaches 101
13.6 Protocol Deviations 101
14. ADMINISTRATIVE AGREEMENTS 102
14.1 Financing of the Clinical trial 102
14.2 Financial Disclosure 102
14.3 Publication Policy / Dissemination of Trial Data 102
14.4 Declaration regarding Data Sharing 102
15. REFERENCES 103
16. APPENDICES 108
16.1 Appendix A 108
16.1.1 Patient Involvement: Letter of support of TSD (Tuberöse Sklerose Deutschland e.V.) 108
16.1.2 Patient Involvement: Letter of support of Achse e.V (Alliance of Chronic Rare Diseases (“Allianz Chronischer Seltener Erkrankungen“)) 109

List of Tables

Table 1: Schedule of assessments –Treatment + SOC group (T) and SOC group (C) 16
Table 2: Sirolimus adverse effects depending on age [45] 31
Table 3: Investigational medicinal product in this trial 46
Table 4: Starting daily dose of IMP 59
Table 5: Sirolimus dose adjustment 60
Table 6: Sirolimus dose modifications for toxicities 61
Table 7: Diagnostic Criteria of TSC 72
Table 8: Power for 30 patients per group under different assumptions 93

List of Figures

Figure 1: PRISMA Search Process 30
Figure 2: Workflow Risk-Benefit-Evaluation 34
Figure 3: Schematic diagram of trial 39
Figure 4: Simulated sirolimus clearances compared with the estimated age-appropriate starting dosing regimens for the target range of 10–15 ng/ml 58

PROTOCOL SYNOPSIS

**EU CT Number**	2022-502332-39-00
**Full Title**	Long-term neuropsychologic outcome of pre-emptive mTOR inhibitor treatment in children with tuberous sclerosis complex (TSC) under 4 months of age (PROTECT)
**Clinical Trial Code**	PROTECT
**Rationale**	Pre-clinical data suggest that neuropsychologic deficits in TSC are attributed to dysregulated mTOR signalling sensitive to mTOR inhibitor treatment. It can be hypothesized that pre-emptive mTOR inhibitor treatment reduces the risk for TSC-related neuropsychologic deficits.
**Primary Objective and Endpoint**	1. Neuropsychologic outcome at 24 months of age assessed by rater blinded neuropsychologic testing measured by the cognitive scale on the Bayley Scales of Infant and Toddler Development III (BSID-III) compared with Standard of Care (SOC) alone.
**Main Secondary Objectives and Endpoints**	1. Neuropsychologic outcome at 12 months of age assessed by rater blinded neuropsychologic testing using the cognitive scale on the BSID-III in both groups. Cognitive impairment will be defined as BSID-III cognitive scale score <70 similar to the EPISTOP trial.<br>2. Adaptive behaviour assessed by the Vineland Adaptive Behaviour Scales (VABS-3; digital) at the age of 12 months and 24 months.<br>3. Evidence for autism spectrum disorder measured at 12 months and 24 months of age by the Autism Diagnostic Observation Schedule (ADOS-2). Suspicion of autism spectrum disorder will be defined as ADOS >12, similar to the EPISTOP trial.<br>4. Evidence for autism spectrum disorder measured by the Modified Checklist for Autism in Toddlers revised (M-CHAT-R/F).<br>5. TSC-associated Neuropsychiatric Disorders (TAND) severity assessed by the TAND-L Checklist (German version) at 12 and 24 months.<br>6. Assessment of seizure frequency and the occurrence/severity of infantile spasms (IS) measured by seizure diaries, care giver questionnaires and electroencephalogram (EEG) recordings.<br>7. Reduction of number and size of cardiac rhabdomyoma and arrhythmia (if present).<br>8. Reduction of cerebral tumor number and size on cranial magnetic resonance imaging (cMRI) (if present)<br>9. Adverse events (AE), serious adverse events (SAE) and Adverse Events of Special Interest (AESI), assessed by the *Common Terminology Criteria of*

	Adverse Events (CTCAE, Version 5.0, or most recent version).<br>10. Assessment of laboratory data and vital signs.<br>11. Assessment of EEG recordings for the occurrence of hypsarrhythmia and epileptiform discharges.<br>12. Renal changes in abdominal sonography: Volume and size of angiomyolipomas (if present), kidney size, renal pelvis dilation, echogenicity.
**Trial Design**	A two-arm, randomized, observer-blind, controlled phase IIb national multicentre clinical trial.<br><br>Patients will remain in the trial for approx. 21-25 months.
**Sample Size**	To be assessed for eligibility (n = 120)<br>To be allocated to trial (n = 60)<br>To be analysed (n = 60)
**Clinical Trial Population**	<u>Inclusion criteria:</u><br>1. Definite diagnosis of TSC according to the 2021 Updated International Tuberous Sclerosis Complex Diagnostic Criteria.<br>2. <4 months of age at the time of enrolment (randomization and treatment initiation must occur before 4 months of age; infants born prematurely must have a corrected age of at least 39 weeks, calculated by subtracting the number of weeks born before 40 weeks gestation from the actual chronological age, in weeks).<br>3. Signed informed consent from legal guardian(s) prior to any study specific procedure.<br><br><u>Exclusion criteria:</u><br>1. Has a TSC-associated condition for which mTOR treatment is clinically indicated, i.e. subependymal giant cell astrocytoma (SEGA).<br>2. Has been treated in the past or is currently being treated at the time of enrolment with systemic mTOR inhibitors (such as rapamycin, sirolimus, or everolimus).<br>3. Contraindication to study medication. Rapamune® oral solution contains soya oil. Patients allergic to peanut or soya must not take this medicine.<br>4. Current enrolment, or observation period of competing clinical trials at any time during enrolment in the study.<br>5. History of significant prematurity, defined as gestational age < 30 weeks at the time of delivery, or other significant medical complications at birth or during the neonatal period that, apart from TSC, would convey additional risk of seizures or

	neurodevelopmental delay (i.e. hypoxic ischemic encephalopathy (HIE), severe neonatal infection, major surgery, prolonged ventilatory or other life-saving supportive care or procedures).<br>6. Abnormal laboratory values at screening (i.e., renal function, liver function, or bone marrow production) that are in the opinion of the investigator clinically significant and may jeopardize the safety of the study subject.<br>7. Parents / caregiver of the child who are, in the opinion of the investigator, unable to comply with the requirements of the study.
**Interventions and Treatments**	Investigational Medicinal Product (IMP): mTOR inhibitor sirolimus<br>Trade name: Rapamune®<br><br>Pal formulation: 1 mg/ml oral solution<br>Routes of administration: oral<br><br>Dosage: Sirolimus will be administered as oral solution adapted to body surface area with a starting dose of 0.5 mg/m^2/day (<1 months of age) to 0.9 mg/m^2/day (3- <4 months of age) divided BID (twice daily) until 2 years of age. Sirolimus 12h trough levels will be measured at every study visit and doses titrated and adapted to reach target trough levels of 5-10 ng/ml.<br><br>Control intervention: standard of care (SOC)<br><br>Treatment duration: starting within the first 4 months of life and continued until the 2nd birthday.
**Ethical Considerations**	Reducing neuropsychologic deficits will improve quality of life and participation, as well as reduce morbidity and mortality, hospitalization, socio-economic costs, and the number of pharmacologic and non-pharmacologic therapies required for the treatment of associated long-term complications.
**Number of Sites**	17-20

SCHEDULE OF ASSESSMENTS

Table 1: Schedule of assessments –Treatment + SOC group (T) and SOC group (C)

**Visit Title**	**Screening**	**Treatment + SOC / SOC**												**Follow-Up**	
**Visit No.**	**Screening**[1]	**V1**[§]	**V2**	**V3**	**V4** [2]	**V5** [3]	**V6**	**V7**	**V8**	**V9**	**V10**	**V11**	**V12**	**End of trial Visit (V13)**	**V14**
**titration phase, then months of life**	-14 to 0 days	Treatment Start	titration in 14-days intervals[#]			m3	m6	m9	m12	m15	m18	m21	m24	m25	m60 optional
**Time Slot [days]**	-14 to 0 days	0	+/-4			+/-7	+/-7	+/-7	+/-14	+/-7	+/-7	+/-7	+/-14	+/-7	+/-30
Informed Consent	X														
Diagnosis of TSC	X														
Inclusion/ exclusion criteria	X														
Relevant medical history/current conditions	X														
Randomization		X													
Previous ASM therapy	X														
Concomitant ASM therapy	X	X	T	T	T	T/C	T/C	T/C	T/C	T/C	T/C	T/C	T/C	T/C	
Other concomitant medications	X	X	T	T	T	T/C	T/C	T/C	T/C	T/C	T/C	T/C	T/C	T/C	
Vital signs [4]	X	X	T	T	T	T/C	T/C	T/C	T/C	T/C	T/C	T/C	T/C	T/C	
Height/weight /OFC	X	X	T	T	T	T/C	T/C	T/C	T/C	T/C	T/C	T/C	T/C	T/C	
Physical examination	X	X	T	T	T	T/C	T/C	T/C	T/C	T/C	T/C	T/C	T/C	T/C	
Neurologic examination	X	X	T	T	T	T/C	T/C	T/C	T/C	T/C	T/C	T/C	T/C	T/C	
Seizure diary [5]	X	X	T	T	T	T/C	T/C	T/C	T/C	T/C	T/C	T/C	T/C	T/C	
Developmental milestones [6]						T/C	T/C	T/C	T/C		T/C		T/C		
**Neuropsychologic evaluation**															
BSID-III									T/C				T/C		
ADOS									T/C				T/C		
VABS									T/C				T/C		T/C*

Visit Title	Screening	Treatment + SOC / SOC												Follow-Up	
**Visit No.**	**Screening[1]**	**V1[$]**	**V2**	**V3**	**V4 [2]**	**V5 [3]**	**V6**	**V7**	**V8**	**V9**	**V10**	**V11**	**V12**	**End of trial Visit (V13)**	**V14**
**titration phase, then months of life**	-14 to 0 days	Treatment Start	titration in 14-days intervals[#]			m3	m6	m9	m12	m15	m18	m21	m24	m25	m60 optional
**Time Slot [days]**	-14 to 0 days	0	+/-4			+/-7	+/-7	+/-7	+/-14	+/-7	+/-7	+/-7	+/-14	+/-7	+/-30
TAND									T/C				T/C		T/C*
M-CHAT-R													T/C		
SCQ															T/C*
QOLCE [7]															T/C*
**Laboratory examinations**															
Hematology [8]	X		T	T	T	T	T/C	T	T/C	T	T	T	T/C	T	
Chemistry [9]	X		T	T	T	T	T/C	T	T/C	T	T	T	T/C	T	
Lipid panel [10]	X			T			T/C		T/C		T		T/C		
Urinalysis [11]	X			T			T/C		T/C		T		T/C		
Vaccination titer optional									T/C				T/C		
RNA optional	X												T/C		
DNA optional	X												T/C		
**Safety assessments**															
Adverse events [12]	X	X	T	T	T	T/C	T/C	T/C	T/C	T/C	T/C	T/C	T/C	T/C	
Individual risk-benefit evaluations							T		T		T				
Sirolimus PK sampling			T	T	T	T	T	T	T	T	T	T	T		
ASM PK sampling[13]	X		T				T		T		T		T		
Therapy (dosing)[14]		T	T	T	T	T	T	T	T	T	T	T	T		
Abdominal sonography[15]	X												T/C		
EEG	X			T			T/C		T/C		T/C		T/C		
cMRI, Echo, ECG [16, 17]	T/C														

X= all participants, C = Control Group, T = Treatment Group, ASM = Anti-seizure medication, SOC: Standard of Care, m: months

* Items will be recorded separate from the eCRF (data of accompanying research after data base lock of main trial)

14-days intervals beginning on day 0

§ **Neonates who receive their first dose of IMP under the age of 4 weeks (corrected gestational age <4 weeks of life):** Caregivers will have up to two additional telephone calls by the study personnel up to the end of the 4th week of life. These calls will take place in those weeks (+/-2 days) where no titration visit takes place. Caregivers will be asked about the condition of their child. If it turns out that any sign occurs that requires an on-site visit, the study staff will act accordingly. At the discretion of the investigator, a blood sample could be taken for a safety laboratory which includes the blood alcohol concentration (BAC) and surrogate parameters for potential propylene glycol (PG) intoxication including electrolytes, osmolality, creatinine, urea, pH, lactate, bicarbonate, anion gap, and the calculated osmol gap.

1. Screening examinations can be conducted up to 14 days before study entry including the day of Visit one (V1). Provided all inclusion criteria are met and the informed consent form (ICF) has been signed, Screening Visit and Visit one (V1) may take place at the same day. All procedures for the screening visit must be performed prior to randomization/administration of first dose.
2. Can be omitted, if target concentration range of IMP was correct without adjustment in V2 and V3.
3. Can be omitted, if the last titration visit was ≤ 1 month ago.
4. Heart rate, blood pressure, temperature.
5. Seizure diaries distributed and /or collected and evaluated.
6. For details see chapter 6.8.
7. For details see chapter 2.2.2.
8. Complete blood count with differential blood count.
9. For details see chapter 6.8.
10. For details see chapter 6.8.
11. For details see chapter 6.8.
12. Graded by the Common Terminology Criteria of Adverse Events (CTCAE, Version 5.0 or most recent version).
13. ASM PK sampling if applicable.
14. Regarding sirolimus dose adjustments, an additional pre-dose PK blood sample should be collected after 14 days, which during the beginning of the trial should coincide with the next regular study visit and after that during an additional appointment.
15. Allowed to be 8 weeks old at day 0.
16. Examinations are carried out as part of the standard of care.
17. Each available cMRI/ECG/Echo carried out has to be entered in eCRF as an unscheduled visit.

[optional] optional intervention within sub-trials, if specific informed consent is given.

ABBREVIATIONS

ADL	Activities of Daily Living
ADOS	Autism Diagnostic Observation Schedule
AE	Adverse Event
AESI	Adverse Event of Special Interest
ALAT/ALT	Alanine Aminotransferase
AMG	Arzneimittelgesetz (German Drug Law)
ASD	Autism Spectrum Disorder
ASM	Anti-Seizure Medication
ASR	Annual Safety Report
ASAT/AST	Aspartate Aminotransferase
ATC	Anatomical-Therapeutic-Chemical Code, part of WHO-DRL (Drug Reference List)
ATNR	Asymmetrical Tonic Neck Reflex
AUC	Area Under the Curve
AxMP	Auxiliary Medicinal Product
BAC	Blood Alcohol Concentration
BDSG	Bundesdatenschutzgesetz (National Regulatory Requirements)
BESA	Brain Electrical Source Analysis
BfArM	Bundesinstitut für Arzneimittel und Medizinprodukte (Federal Institute for Drugs and Medical Devices, Germany)
BID	twice daily
BMBF	Bundesministerium für Bildung und Forschung (Federal Ministry for Education and Research, Germany)
bmp	Beats Per Minute
BOOP	Bronchiolitis Obliterans with Organizing Pneumonia
BSA	Body Surface Area
BSID	Bayley Scales of Infant and Toddler Development
[c.]	Complementary (c) DNA according to HGVS [1]
CD	Compact Disc
CONSORT	Consolidated Standards of Reporting Trials
CI	Confidence Interval
CL	Clearance
C_{max}	Maximum Serum Concentration
C_{min}	Minimum Serum Concentration

cMRI	Cranial Magnetic Resonance Imaging
CRF	Case Report Form
CsA	Ciclosporin A
CTCAE	Common Terminology Criteria of Adverse Events
CTFG	Clinical Trial Facilitation Group
CTIS	Clinical Trials Information System
CTR	Clinical Trial Regulation (Regulation (EU) No 536/2014)
CV	Curriculum Vitae
DBL	Data Base Lock
DFG	Deutsche Forschungsgemeinschaft (German Research Foundation)
DMP	Data Management Plan
DNA	Deoxyribonucleic Acid
DSGVO	General Data Protection Regulation
DSMB	Data Safety Monitoring Board
DVP	Data Validation Plan
eCRF	electronic Case Report Form
EC	Ethics Committee
ECG	Electrocardiogram
EDTA	Ethylene Diamine Tetraacetic Acid
EEG	Electroencephalography
EMA	European Medicines Agency
ENST	Ensembl Transcript
EURD	European Union Reference Dates
FAS	Full Analysis Set
FD	Financial Disclosure
FDA	Food and Drug Administration
FSI	First Subject In
G1-Phase	Gap-Phase
GCP	Good Clinical Practice
GDPR	General Data Protection Regulation (EU Datenschutz-Grundverordnung (DSGVO))
HGVS	Human Genome Variation Society
HIE	Hypoxic Ischemic Encephalopathy
HRQoL	Health Related Quality of Life

IB	Investigator´s Brochure
IBD	International Birth Date
ID	Intellectual Disability
ICD	International Classification of Diseases
ICF	Informed Consent Form
ICH	International Council on Harmonization of Technical Requirements for Registration of Pharmaceuticals for Human Use
ICH GCP	ICH harmonized tripartite guideline on GCP
ICMJE	International Committee of Medical Journal Editors
IDDM	Insulin-dependent Diabetes Mellitus
IMP	Investigational Medicinal Product
IMPD	Investigational Medicinal Product Dossier
INN	International Nonproprietary Name
IQ	Intelligence Quotient
IRT	Interactive Response Technology
IS	Infantile Spasm
ISF	Investigator Site File
ISRCTN	International Standard Randomized Controlled Trial Number
ITT	Intention To Treat
IV	Intravenous
KKS	Coordination Centre for Clinical Trials (Koordinierungszentrum für Klinische Studien)
LDH	Lactate Dehydrogenase
LDL	Low-Density-Lipoprotein
LDSG BW	Landesdatenschutzgesetz Baden-Württemberg
LSI	Last Subject In
LSO	Last Subject Out
MedDRA	Medical Dictionary for Regulatory Activities
MRI	Magnetic Resonance Imaging
M-CHAT-R	Modified Checklist for Autism in Toddlers revised
mRNA	Messenger Ribonucleic Acid
mTOR	Mammalian Target of Rapamycin
OFC	Occipitofrontal Head Circumference
[p.]	protein (letter prefix indicating a protein reference sequence, according to the Human Genome Variation Society (HGVS) recommendations for the description of sequence variants)

PCNA	Proliferating Cell Nuclear Antigen
PEI	Paul-Ehrlich-Institut
PG	Propylene Glycol
P-gp	P-Glykoprotein
PI	Principal Investigator
PK	Pharmacokinetic
PO	Oral
PP	per-Protocol
PPS	per-protocol set
PSTE	Potential Sight Threatening Event
PV	Pharmacovigilance
Q	quarter (time span)
QoL	Quality of Life
QOLCE	Quality of Life in Childhood Epilepsy Questionnaire
RACT	Risk Assessment and Categorization Tool
RBC	Red Blood Cells
RDE	Remote Data Entry
RNA	Ribonucleic Acid
RSI	Reference Safety Information
S-Phase	Synthesis Phase
SAB	Scientific Advisory Board
SAE	Serious Adverse Event
SAF	Safety Analysis Set
SAP	Statistical Analysis Plan
SAR	Serious Adverse Reaction
SC	Steering Committee
SCQ	Social Communication Questionnaire
SEGA	Subependymal Giant Cell Astrocytoma
SmPC	Summary of Product Characteristics
SLE	Systemic Lupus Erythematosus
SOC	Standard of Care
SOP	Standard Operating Procedure
SUDEP	Sudden Unexpected Death in Epilepsy
SUSAR	Suspected Unexpected Serious Adverse Reaction
SVL	Severe Visual Loss

TAND	Tuberous Sclerosis Complex-associated Neuropsychiatric Disorders
t_{max}	Time at which C_{max} is observed
TSC	Tuberous Sclerosis Complex
TSD	Tuberöse Sklerose Deutschland e.V.
TMF	Trial Master File
US	United States
UV	Ultraviolet
VABS	Vineland Adaptive Behavior Scales
V	Visit
Vss	Volume of Distribution at Steady State
WB	Whole Blood
WBC	White Blood Cells
WHO	World Health Organization
QOLCE	Quality of Life in Childhood Epilepsy

1. INTRODUCTION AND RATIONALE

1.1 Scientific Background

Tuberous sclerosis complex (TSC) is an autosomal dominant neurodevelopmental disorder affecting 1:6,760 – 1:13,520 live births in Germany [2] with an estimated prevalence of 1:20,000 individuals equally distributed among ethnic groups and both sexes. Penetrance and expressivity are variable ranging from mild skin lesions and minimal neurologic signs to severe neurodevelopmental manifestations, including refractive epilepsy, autism spectrum disorder (ASD) and profound intellectual disability (ID) [3, 4]. Mortality is significantly elevated, up to fivefold compared to the general population. The most common causes of TSC-attributable deaths are status epilepticus, sudden unexpected death in epilepsy (SUDEP), kidney complications, and infections. Mortality is especially increased in individuals with ID [5]. About 90% of TSC patients develop neurologic complications, such as epilepsy, ID and ASD [3, 6]. Epilepsy, usually starting during the first year of life, occurs in 83.5% of patients and is the most prevalent and challenging manifestation [3, 7]. Early-onset epilepsy typically presents with focal seizures and infantile spasms (IS). IS or West syndrome is a severe epileptic encephalopathy characterized by epileptic spasms and a pathognomonic EEG pattern (hypsarrhythmia), occurring in 38.8% of affected individuals [7]. Infantile onset epilepsy and in particular IS are suspected to cause alterations in brain maturation with significant impact on neurodevelopment and are considered the most important risk factors for neuropsychologic impairment (ID and ASD) [8]. On the molecular level, TSC is caused by heterozygous loss-of-function variants in the genes *TSC1* and *TSC2*, encoding the Tuberin-Hamartin complex, acting as a critical upstream suppressor of the mammalian target of rapamycin (mTOR), a key signaling pathway controlling cellular growth and metabolism. Haploinsufficiency of *TSC1* or *TSC2* leads to a constitutive activation of the mTOR pathway, resulting in fundamental alterations in neuronal network properties and an imbalance in neuronal excitation and inhibition, acting as a common pathway toward epilepsy, ID and ASD [9, 10]. Therapeutic mTOR inhibition is a promising molecular target for the treatment of TSC-associated manifestations. The mTOR inhibitors sirolimus and everolimus have been increasingly used in TSC and have gained approval for the treatment of TSC-related manifestations, such as subependymal giant cell astrocytomas (SEGA), renal angiomyolipomas and recently, refractory focal epilepsy above the age of 2 years. With respect to neuropsychological manifestation, preliminary trials with mTOR inhibitors in TSC children above 2 years have so far failed to demonstrate beneficial effects. This might be due to the fact that the window of opportunity for the neuromodulatory effects of mTOR inhibitors lies within the first year of life before TSC-related early-onset epilepsy and in particular IS occur as sign of disrupted brain maturation and synaptic formation. Since the influence of mTOR inhibitors on the emerging neuronal network disorder is not fully understood, the exact time at which a therapy has to be started cannot be clearly delineated. Considering the underlying molecular pathomechanisms of TSC and the fact that mTOR inhibitors can reverse many TSC-related manifestations in cell and animal models as well as in anecdotal reports [11-13], it can be hypothesized that pre-emptive initiation of mTOR inhibitor treatment reduces the risk for TSC-related neuropsychologic deficits. To date, there are no published controlled, prospective clinical trials evaluating the effect of pre-symptomatic mTOR inhibitor therapy on neuropsychologic manifestations in TSC patients under 2 years of age.

1.2 Clinical Trial Rationale

Neuropsychologic impairment in TSC exerts a large burden of disease on affected individuals, their families and the health care system. According to the EPISTOP trial [14], a long-term prospective study on TSC, only 54% of TSC children at 2 years of age showed normal cognitive development, while severe cognitive deficits with a developmental quotient < 70 measured by the Bayley Scales of Infant and Toddler Development (BSID) or ASD measured by the Autism

Diagnostic Observation Schedule (ADOS) were present in 46% of cases [15]. Similarly, the TOSCA study, the largest natural history study on TSC, reported mild to profound degrees of ID in 55.6% [16]. According to a meta-analysis the burden of disease in TSC is at least twice as high as in the general population. Thereby, children with ID, ASD and epilepsy have a high risk for hospitalization and are more likely to require intensive care treatment [5]. Outpatient visits were even more frequent, due to the high number of specialists that are required in the multimodal care. Quality of life (QoL) is reduced by a high number of neurotropic medications and more diagnostic procedures than in the general population. Global intellectual impairment in TSC is highly correlated to a history of IS and severe intractable seizures [17]. 74.4% of TSC patients with IS develop cognitive impairment in contrast to 39.2% without IS [4, 18]. Furthermore, a higher incidence of ASD is reported in TSC patients with early-onset intractable epilepsy [19]. Antiepileptic treatment has proven to stop IS in 71.5% of cases and showed mitigating effects with respect to ID and ASD [7, 20]. In 2019, Jozwiak and colleagues suggested that pre-symptomatic antiepileptic treatment can reduce occurrence and severity of cognitive impairment in TSC [21]. Recently, the EPISTOP trial published the results of a controlled multicenter study comparing the preventive effect of the antiseizure medication vigabatrin, started after detection of first epileptiform activity on electroencephalography (EEG), versus conventional treatment, on TSC-related epilepsy. The trial showed that preventive treatment was associated with a >2-fold reduction in the risk of drug-resistant epilepsy compared to conventional management. Neurodevelopmental delay at age 2 years was seen in 33% of those on preventive treatment and 50% of those on conventional treatment, however without reaching significance on statistical testing [14]. Two major messages can be derived from this trial. First, the time at onset of epileptiform activity on EEG might be too late for preventive ASD treatment, since aberrant neurophysiological changes that cannot be reversed by treating seizures might have already occurred. Second, isolated antiepileptic treatment with vigabatrin is not sufficient to improve neuropsychologic outcome, highlighting the need for mechanistic and disease modifying therapies. mTOR inhibitors present a promising targeted therapy in TSC and have shown encouraging results in reducing TSC-related epilepsy in patients older than 2 years [22, 23]. Neuropsychologic symptoms were not impacted by treatment, possibly due to the fact that TSC-related implications on brain maturation, laying the structural basis for neuropsychologic impairment, occur before onset of epilepsy [9]. Delayed initiation of mTOR inhibitors might therefore fail to restore cognitive function. There is promising pre-clinical data suggesting that the extent of neuropsychologic impairment in TSC is associated to dysregulated mTOR signaling and that mechanistic treatment with mTOR inhibitors can attenuate some of those symptoms. In *Tsc1* mutant mice depicting abnormal behavior, representative for ASD, mTOR inhibitor treatment starting on postnatal day 7 was able to prevent aberrant neurodevelopment [24]. Additionally, ASD-like phenotypes as well as deficits in learning and memory in *Tsc2* haploinsufficient animal models were reversible with mTOR inhibitor treatment [25, 26]. Clinical trials evaluating these effects in infants and young children remain elusive.

1.3 Risk Benefit Assessment

The primary goal of this study is to assess the benefits of pre-emptive mTOR inhibitor therapy on neuropsychologic outcomes in TSC. As detailed above, neuropsychological impairment in TSC exerts a significant burden of disease, limiting lifespan of affected individuals and families' quality of life. The neuropsychologic deficits observed in TSC are at least in part attributed to mTOR pathway activation and animal models recapitulating the neuropathology of TSC show sensitivity to mTOR inhibitor therapy as described above. Yet, current treatment options do not adequately target the primary signaling pathway that is suspected to trigger neuropsychologic deficits. Encouraging evidence comes from preclinical trials [11-13] and small case series [27, 28] that justify the clinical evaluation of mTOR inhibitors in infants and young children with TSC to improve neuropsychologic outcome.

The neuropsychological development is the main outcome parameter of our study, as it is impaired in around 46% of adult individuals with TSC [29], [30], which represents a high risk for all newborns with TSC. To date, no biomarker nor prognostic factor has been identified that is useful to identify individuals with TSC at risk for abnormal neurodevelopment. Identification of novel biomarkers together with explanations for the phenotypic heterogeneity in TSC are therefore major research priorities in TSC [31]. As no valid individual biomarker can discern infants at risk for neurodevelopmental disorders, no stratification can be applied and all newborns and infants before 6 months of age are at similar risk. This high individual risk about 46% for impaired neurofunction justifies our strategy to include all infants before 4 months with a diagnosis of TSC.

All infants with TSC have a relevant risk for additional manifestations of disease, which includes a very high risk for epilepsy up to 90% [18], being often refractory to treatment. Nearly all individuals with TSC also develop cerebral malformations (>90%) and cardiac rhabdomyoma (>90%) with a risk for arrythmia and heart failure and about one quarter of individuals will develop SEGA during childhood. Half of affected individuals develop facial angiofibroma and renal angiomyolipoma until adolescence. All of these manifestations are associated with high morbidity and mortality (renal angiomyolipoma) and affect the quality of life of individuals with TSC [29], [5]. Overall, TSC-related morbidity from organ manifestations outside functional neurodevelopmental disorders is highly relevant. As mTOR inhibitor treatment has been shown and has been repeatedly reported with beneficial effects on all these manifestations [28] it can be assumed that all infants and children can have positive effects from the study intervention with sirolimus. It is even hypothesized that long-term positive disease modification can be achieved with mTOR inhibition in TSC [31].

There is a risk for SAEs during treatment with sirolimus and there have been single reports on death in association to sirolimus treatment. Rössler et al. 2021 [32] described SAE in a cohort of children with vascular anomalies and described cases of severe pneumonia. They provide a review on previous deaths during sirolimus treatment, that occurred in young children with kaposiform hemangioendothelioma, which itself is associated with a high mortality. Larger studies assessing the risk-benefit for sirolimus in infants with kaposiform hemangioendothelioma described mainly mild side effects and support sirolimus treatment [33].

In TSC, there have been no reports on death in childhood related to sirolimus treatment so far. Deaths, that occurred during long-term safety studies [29] in mTOR inhibitor treatment with everolimus were not considered to be related to treatment.

Regarding the risk for malignancy, data is scarce and not conclusive for a potential risk-benefit analysis. There are data that therapy with sirolimus after specific organ transplantation can be associated with an increased risk of malignancy [34], however this can be outweighed by the positive effects of mTOR inhibitor treatment on proliferation risk in TSC. TSC itself bears a risk for proliferating disease and specific tumors and current meta analyses support that mTOR inhibitors are associated with tolerable side effects and may be beneficial in TSC-associated malignancies [35].

1.3.1 Study-associated burdens and procedures

Current SOC recommends close monitoring of infants and children with TSC in a collaborative and interdisciplinary healthcare setting during their first two years of life. This includes routine clinical evaluations and EEG recordings, which take place every 4-6 weeks in the first year, and every 8-12 weeks in the second year. The majority of study-related procedures include regular outpatient clinic visits, medical history and seizure diary assessment, measurement of vital signs and anthropometric data, thorough physical and neurological examination, developmental assessment, EEG follow-ups, and imaging studies such as MRI, ECG/Echo, and abdominal ultrasonography – all of which are required as part of the SOC of TSC patients. In the treatment

group, blood tests are conducted more frequently to monitor the levels of the study medication and ensure optimal dosing and identify possible safety-relevant parameters. This may cause discomfort or pain and rare cases of bruising, local irritation, inflammation or bleeding during blood sampling. In very rare cases, injury to a superficial nerve may occur, causing superficial sensory disturbances. No placebo will be administered in the control group. This will minimize the number of laboratory examinations at screening, 6 months, 12 months, and 24 months of age to only those required to control for laboratory abnormalities unrelated to the study intervention. The study medication carries the risk of potential side effects and may cause additional discomfort when administered twice daily, which was however shown to reduce total doses as opposed to a once daily regimen (detailed below). At 12 months and 24 months of life, detailed neuropsychological evaluations will be conducted in an outpatient setting to assess the child's developmental status and behavior. These evaluations may take several hours. In addition, caregivers are asked to answer several questionnaires about the developmental status and behavior of their children, which might take up to 90 minutes.

1.3.2 Clinical impact

This trial will provide detailed information about benefits and safety of prophylactic mTOR inhibitor treatment in reducing the occurrence and severity of neuropsychologic impairment in TSC. Using a rational, mechanism-based therapeutic strategy, this trial could fundamentally transform the therapeutic management of TSC from a symptomatic to a targeted disease-modifying approach. Eventually, TSC represents a disease, where early intervention can improve long-term performance in school, social and professional life and can serve as a model for targeted mechanism-driven approaches for related neurodevelopmental disorders with and without dysregulated mTOR signaling.

1.3.3 Patient benefit

Reducing neuropsychologic deficits can improve quality of life and participation, as well as reduce morbidity and mortality, hospitalization and the number of pharmacologic and non-pharmacologic therapies required for the treatment of associated long-term complications.

The study group around He *et al*. assessed the effect of sirolimus on reduction of seizure frequency in TSC and reported the highest response rates within the first 2 years of life which supports our hypothesis that early initiation of mTOR inhibitor treatment yields the highest benefit. This study reported no Grade 3 or higher AEs. The authors report beneficial effects on neurodevelopment, identifying early treatment initiation as major contributor for improving neurocognition [36]. Based on the results of the EPISTOP study [14], two important messages for the treatment of TSC can be derived: First, neurophysiological changes that have already occurred at the time of seizure onset, might not be reversible with conventional antiepileptic treatment, highlighting the potential need for innovative and preventive treatment strategies. Second, antiepileptic treatment alone might not be sufficient to improve neuropsychologic outcomes, emphasizing the need for multimodal approaches and disease modifying therapies. mTOR inhibitors have shown encouraging results in reducing TSC-related epilepsy in patients older than 2 years of age [22, 23] and could serve as mechanism-based preventive treatment for TSC.

1.3.4 Inclusion of minors

Inclusion of minor participants is justified under §20 of the Declaration of Helsinki because the research project specifically responds to the health needs and priorities of this group and the research project cannot be conducted in a nonvulnerable group (adults only). The development of epileptic encephalopathy and mental retardation originates in infancy and early childhood. In order to investigate the influence of the study medication on these endpoints, it is essential to include pediatric subjects. The current evidence and assessment of therapeutic approaches in this age

group is incomplete. It is expected that medical findings resulting from this study will have a future benefit for this patient group.

Measures to reduce distress and pain

The study design is centered on minimizing stress, pain, discomfort, and anxiety among study participants. Infants and toddlers with TSC are typically closely monitored during the first two years of life within standard care: According to the current treatment standard for TSC patient´s electroencephalography (EEG) examinations and clinical assessments are conducted every 4-6 weeks in the first year of life and every 8-12 weeks in the second year. As a result, most study-related assessments (regular outpatient clinic visits, medical history discussions, seizure diary, blood pressure and heart rate measurements, height, weight, and head circumference measurements, physical and neurological examinations, developmental assessments, EEG measurements, kidney ultrasound, head MRI (magnetic resonance imaging), and cardiac ultrasound) do not differ from those required in the context of standard treatment. Parents will be informed whether a procedure is part of usual care or the study. All examinations are conducted using size- and age-appropriate tests, materials, and devices. Study sites are equipped for pediatric care, and personnel are trained in pediatric care and supervised by experienced health experts.

Qualified study personnel with at least several years of experience in pediatric care and specific knowledge of TSC and the aforementioned measurements and interventions (such as blood sampling in neonates) will minimize emotional and physical pain. Blood samples for the study are taken at the same time as those required for usual clinical care. In consultation with caregivers, the use of anesthetic plasters to minimize pain is encouraged. Emphasis is placed on communicating with parents so they are informed about the procedures. Separation of infants from parents or familiar individuals is avoided whenever possible. Facilities are designed to be child-friendly, providing a comfortable environment with appropriate furniture and toys, while avoiding cold, light, and noise. Blood collection is carefully managed, with the maximum amount based on age and weight to ensure safety. The research-related blood loss (including waste) does not exceed 3% of the total blood volume over a period of four weeks and should not exceed 1% at any time. In the group receiving the study medication (treatment group), apart from general blood tests, regular determinations of the study medication's concentration and safety-related blood parameters are collected and analyzed to control the dosage of the study medication. The number of tests and assessments is minimized whenever possible. A single blood sample (SOC and PK sampling) comprises approximately 5-10 ml. Throughout the entire study period of 21-24 months, these measurements are conducted 13 times in the group receiving the study medication, totaling approximately 65-130 ml during the study. By not including a placebo group, the number of blood collections is reduced in the comparison group. Throughout the entire study period of 21-24 months, these measurements are conducted 4 times in the group not receiving the study medication, totaling 20-40 ml during the study.

Age-appropriate formulas are used for calculating the glomerular filtration rate (GFR), and laboratory reference ranges are adjusted to the pediatric age groups. The chosen initial dose is scientifically justified within the protocol, utilizing a pharmacokinetic/pharmacodynamic model for age-adjusted dosing. [37]

1.3.5 Socioeconomic impact

Due to the chronic disease course and multiorgan involvement, TSC patients incur costs at least twice as high as in the general population [5]. These are comprised of direct costs of inpatient/outpatient visits, medication and treatments (physiotherapy, occupational and speech therapy), and indirect costs due to loss of productivity (days off work, reduced working hours, job

loss of patient or caregiver). Reducing the prevalence of neuropsychologic deficits will therefore lower both direct and indirect costs.

1.3.6 Rational for study medication

Two mTOR inhibitors are available for the treatment of TSC, sirolimus and everolimus. These two medications differ only by one additional ethylester group in everolimus, resulting in differences in their half-lives, solubility, elimination, interindividual tolerability and efficacy. Everolimus is currently approved for the treatment of most TSC-manifestations, including the treatment of SEGAs for all age groups, mostly because Novartis Pharmaceuticals, that developed everolimus, supported the key clinical studies leading to approval by the FDA and EMA [38]. Similar trials with sirolimus failed due to lack of funding and the only approved indication for sirolimus is spontaneous pulmonary lymphangioleiomyomatosis due to proposed somatic mutation of TSC [39]. Direct head-to-head clinical trials comparing both medications in TSC are not available and most authors treating older children and adults, currently prefer everolimus, due to the more robust clinical trial experience [40]. However, sirolimus holds a key advantage over everolimus, in that it is available as oral solution (Rapamune®) facilitating the careful administration of precise doses in neonates and infants. Everolimus, by contrast, is only available as tablet with limited solubility in water, which renders precise dosing difficult and bears higher potential risk of accidental application of inaccurately low or high doses. In addition, sirolimus and everolimus work in a similar fashion, and are considered equally effective and interchangeable by leading TSC experts (reviewed in detail by Franz *et al.* [41]), supported by recent evidence of equal tolerability and efficacy of sirolimus in TSC-related seizures [36, 42]. Furthermore, due to the significant higher cost for everolimus for this publicly funded trial, the investigators favor the use of sirolimus.

1.3.7 Safety of sirolimus in neonates and young infants

1.3.7.1 Systematic research for evidence synthesis

To obtain a full synthesis of the available evidence for the risk and side effects, as well as long-term effects of Sirolimus in infancy and early childhood, we conducted a systematic literature review. The databases MEDLINE via PubMed, CENTRAL via Cochrane Library and Embase were searched for reviews and clinical trials on January 15, 2023.

Search terms:
#1 sirolimus/
#2 rapamune/
#3 mammalian target of rapamycin inhibitor/
#4 OR/1-3
#5 risk assessment*/
#5 safety/
#6 adverse event*/
#7 long term outcome*/
#8 OR/5-7
#9 AND/4,8

Filters:
#10 cochrane review
#11 controlled clinical trial
#12 review
#13 systematic review
#14 randomized controlled trial
#15 meta-analysis
#16 OR/10-15
#17 embryo
#18 fetus
#19 infant
#20 newborn
#21 preschool
#22 OR/17-21
#23 [2000-2023]/py)
#24 AND/23

Figure 1: PRISMA Search Process

The following PRISMA 2009 Flow-diagram summarizes the search process [43]:

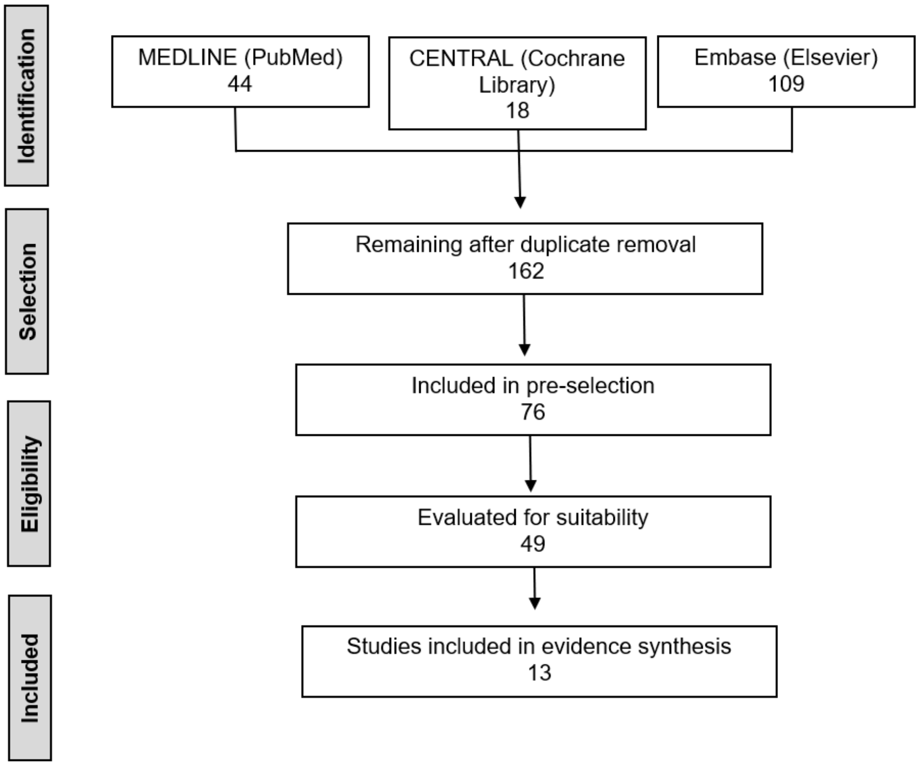

Reports of mTOR inhibitor therapy in neonates with TSC predominantly focus on the treatment of cardiac rhabdomyomas, however raise no major safety concerns (summarized in [28]). Due to the absence of safety and exploratory studies on mTOR inhibitors in infants and young children, these therapies are usually prescribed off-label [44]. To address this issue, we recently published data on the safety and efficacy of mTOR inhibitor treatment in 17 young children with TSC under 2 years. Treatment was well tolerated and most adverse events (AEs), rated by the *Common Terminology Criteria of Adverse Events (CTCAE, current Version)* were limited to mild (grade 1) and moderate (Grade 2). Adverse events included mild transient stomatitis (2 cases), worsening of infantile acne (1 case), increases of serum cholesterol and triglycerides (4 cases), changes in serum phosphate levels (2 cases), increase of cholinesterase (2 cases), transient neutropenia (2 cases), transient anemia (1 case), transient lymphopenia (1 case) and recurrent infections (7 cases). No grade 3-4 adverse events were reported [28]. In a study of 45 infants reported by Krueger and colleagues, 7 patients experienced severe (grade 3) AEs, including ulcers/stomatitis (N = 1), upper respiratory tract infections (N = 2), gastroenteritis (N = 1), other infections (N = 1), abnormal complete blood count (N = 1) and elevated cholesterol (N = 1). No life-threatening (grade 4) AEs or death/disability (grade 5) were reported in either study [27]. A recent two-center retrospective data analysis of medical records of individuals with TSC who were started on sirolimus under the age of two years, reported at least one adverse event in all participants (n=21, median age at onset of sirolimus 104 days). The majority of adverse events were graded as 1 and 2 according to the CTCAE. No adverse events grade 4 or higher were reported. In all patients, sirolimus was administered once a day as oral solution (Rapamune® 1 mg/mL). Initial doses were calculated to be 0.5 mg/m^2/day and varied from 0.01 to 0.07 mg/kg/day. Dosing was adjusted according to sirolimus plasma levels. Trough levels were targeted to range between 3–4 ng/mL, with median plasma levels throughout the study of 4.34 ng/mL (0.79–13.89 ng/mL). The most prevalent AEs were anaemia, thrombocytosis, and hyperlipidemia (see Table 2). Infections and mouth ulcerations, often reported in older patients, were infrequent and of mild or moderate grade. The authors concluded, that adverse effects associated with sirolimus use in infants and young children with TSC are frequent yet not life- or health-threatening. [45]

Table 2: Sirolimus adverse effects depending on age [45]

Adverse Effect | Age	0–6 Months	6–12 Months	12–24 Months	24–36 Months
	N [1] (%)	N (%)	N (%)	N (%)
Hyperlipidemia	10/12 (83.33)	11/13 (84.62)	13/13 (100)	5/8 (62.5)
Hypercholesterolemia	8/12 (66.67)	5/12 (41.67)	8/13 (61.54)	3/8 (37.5)
Elevated LDL	5/11 (45.45)	5/12 (41.67)	7/13 (53.85)	2/7 (28.57)
Hypertrigliceridemia	7/12 (58.33)	8/12 (66.67)	10/13 (76.92)	3/7 (42.86)
Anemia	11/12 (91.67)	8/14 (57.14)	9/14 (64.29)	5/10 (50)
Thrombocytosis	7/12 (58.33)	5/14 (35.71)	11/14 (78.57)	7/10 (70)
Neutropenia	5/12 (41.67)	3/14 (21.43)	5/14 (35.71)	1/10 (10)
Elevated D-dimers	2/2 (100)	0/1 (0)	1/2 (50)	0/0 (0)
Elevated bilirubin	3/7 (42.86)	0/4 (0)	0/4 (0)	1/6 (16.67)
Low ALT	4/12 (33.33)	4/13 (30.77)	6/12 (50)	4/7 (57.14)
Elevated AST	2/12 (16.67)	1/13 (7.69)	4/12 (25)	2/7 (28.57)
Elevated fibrinogen	0/6 (0)	1/2 (50)	1/3 (33.33)	0/1 (0)
Infections	2/12 (16.67)	3/13 (23.08)	5/13 (38.46)	5/10 (50)
Mouth ulcers	2/11 (18.18)	0/14 (0)	0/13 (0)	1/10 (10)

[1] N—number of patients who reported the adverse effect divided by the number of patients who were tested for the adverse effect.

Along these lines, Overwater and colleagues conducted a clinical trial investigating whether sirolimus can reduce seizure frequency in children and infants with TSC [42]. The trial showed potential beneficial effects on seizure reduction, however, likely due to the small number of children enrolled (N = 23), failed to reach significance. No changes in cognitive development were observed, however, the youngest child enrolled was 1.8 years old, and most children already suffered from severe intellectual disability at the time of inclusion, thus likely limiting therapeutic effects. All patients in this trial reported at least one AE, most commonly upper respiratory tract infections, gastrointestinal problems and acne-like lesions. Severe AEs occurred in 5 patients, including aphthous ulcers (N = 2), pneumonia requiring hospitalization, upper respiratory tract infection and increase in seizure frequency, thus requiring discontinuation of treatment in these 5 cases. Further evidence for the use of mTOR inhibitors in young children comes form He *et al*., who evaluated the effect of sirolimus on reduction of seizure frequency in 91 TSC-patients between 2 months and 11.3 years (median 21.2 months), reporting the highest response rates within the first 2 years of life, thus supporting the hypothesis that early initiation of mTOR inhibitor treatment yields the highest benefit [36].

For further information to the excipients and their potential adverse effects of Rapamune® (contains up to 25 mg ethanol and 350 mg propylene glycol (PG; E1520) per ml), see chapter 5.2.2.7.

1.3.8 Long-term tolerability of sirolimus in neonates and young infants

Information on the tolerability and long-term effects of sirolimus on growth, development, and maturation of relevant organ systems in this young age group is limited. Maria *et al.* presented data of a 5-year follow-up study of 22 children with hyperinsulinemic hypoglycaemia (CHI) with a median age at start of sirolimus treatment of 3.87 months and a median starting dose of 0.96 $mg/m^2/d$ [46]. Side effects of treatment occurred in 86.4% with a median delay of 13 months (range 1-52). Infections were most commonly reported (17/22). No growth disturbances or failure to thrive were observed. A review of the off-label use of sirolimus und everolimus in 101 published reports of diverse indications, including 255 children with TSC treated with sirolimus at a median dose of 3.6 $mg/m^2/d$, did not report relevant long-term use complications of treatment [44]. The experience with sirolimus in kidney transplant patients shows a good overall safety profile and was associated with a lower risk of developing malignancies compared to other immunosuppressants [47]. A retrospective, multicenter chart review of severe adverse events (SAE) associated to sirolimus “off-label” therapy for vascular anomalies identified 17 SAEs in 14 patients. The age at initiation of sirolimus therapy was under 2 years (n = 5), 2–6 years (n = 5), and older than 12 years (n = 4). SAEs occurred during the first 3 months of therapy (n = 7), between 3 and 12 months (n = 7) and after 1 year of therapy (n = 3). The most frequent SAE was viral pneumonia (n = 8) resulting in one death due to a metapneumovirus infection in a 3-month-old child and a generalized adenovirus infection in a 28-month-old child. Sirolimus blood level at the time of SAEs ranged between 2.7 and 21 ng/L [32]. In a study of pediatric liver transplant recipients (n=50) under therapy with everolimus in combination with reduced tacrolimus or cyclosporine A, most male patients (87.5%) had testosterone levels below normal range. This was not associated to changes in height or weight percentiles. Among patients available for Tanner staging after 12 months of therapy, all patients aged <8 years belonged to Tanner stages 1 or 2. No events of abnormal sexual maturation were reported during the study period. Although limited by sample size, these findings indicate that combination of mTOR inhibitors such as everolimus with tacrolimus or cyclosporine A does not appear to negatively impact linear growth or sexual maturation in pediatric liver transplant recipients up to 12 months post treatment initiation [48]. Other clinical data on potential adverse effects on longitudinal growth in renal transplant recipients is inconclusive [49, 50], and information on the effects of low-dose treatments with sirolimus on organ development and growth is lacking. Given the lack of data, we additionally assessed available information from

our previously published cohort of early treated infants and children (Saffari et al 2019). We analysed available novel data on growth (height, weight) and also on cardiac function (data from original echocardiographies with markers for hypertrophy and ejection fraction) and compared it to controls. Considering the limitation from the small sample size, we were not able to identify specific safety concerns in our cohort for effects on growth parameters or cardiac function (personal data).
The TOSCA-PASS registry as a post authorization safety study to document the long-term safety and tolerability of everolimus (as similar mTOR inhibitor with a comparable profile) provided the final report in June 2020 [29]. While the data was still limited to draw general conclusions, no additional concerns were reported, 3 deaths in adult patients with TSC and everolimus were not attributed to the medication and no novel concerns were raised, regarding the long-term use of the mTOR inhibitor everolimus. There is no systematic data available, that assessed the impact of the therapeutic range to the long-term tolerability of sirolimus in neonates and young infants.
Taking into account this body of evidence from the literature, including work from others and our group [27, 28], two clinical trials in the United States (ClinicalTrials.gov: NCT05104983) and Poland (ClinicalTrials.gov: NCT04987463) are currently evaluating whether infantile-onset sirolimus treatment can prevent/delay seizure onset in TSC, using a very similar study design.

1.3.9 COVID-19 related risks

The current COVID-19 pandemic is not expected to bring any additional risks to the study. Participants in the treatment group will need to visit the outpatient clinic more frequently during the 6-week dosing phase, compared to SOC. However, these visits cannot be carried out over the telephone as a physical examination and the collection of blood samples are required. While the study medication may have possible immunosuppressive effects, particularly at higher doses, study results in elderly patients suggest a possible protective effect with respect to SARS-CoV-2 infections at lower doses [51]. The impact of the medication on the risk of acquiring COVID-19 in infants and young children is yet to be determined.

1.3.10 Individual risk-benefit evaluations

In order to continuously monitor risk-benefit ratios of participants in the treatment group, the following procedures are implemented into the study visits:

1. At each study site, two subinvestigators with clinical experience in the treatment of children with TSC will evaluate the individual risk-benefit profile of the participants treated with the IMP at 6, 12, and 18 months of life, considering the individual IMP pharmacovigilance as well as possible novel findings in the research field being considered as relevant for the respective individual. These evaluations are implemented into the study visits V6, V8, and V10. If one of the evaluating pediatricians comes to the decision that the respective participant is unlikely to benefit from continuation of IMP application, the treatment will be terminated. The participant will be continued receiving SOC and will be followed-up until the primary endpoint of the study. The results from each risk-benefit evaluation will be documented in the eCRF and on a separate, signed worksheet at the respective study visit. All decisions will be reported to the coordinating investigator.
2. For independent supervision of the process, risk-benefit evaluations of the study sites will also be assessed by the DSMB within the scope of the regular meetings to minimize assessment bias risk at the study sites and to identify participants-at-risk with a comparable safety profile.
3. The coordinating investigator will continuously secure implementing novel knowledge on early prognostic factors that help to assess the individual child’s risk for abnormal neurodevelopment as well as current knowledge on the risk profile of the IMP in infants. Novel findings will be shared and discussed with the Scientific Advisory Board and additionally with the DSMB and the other investigators in a timely manner.

Figure 2: Workflow Risk-Benefit-Evaluation

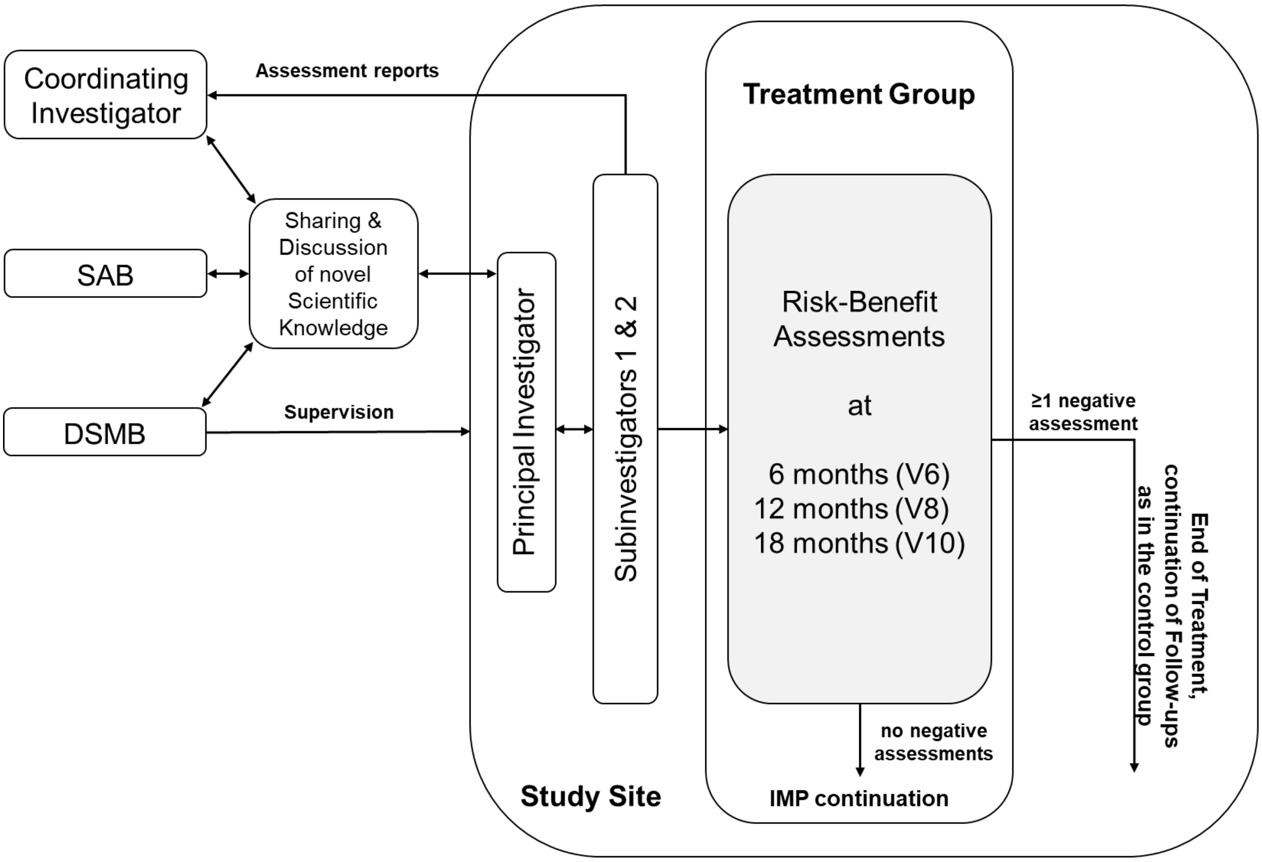

2. Clinical Trial Objectives and Endpoints

2.1 Primary Objective and Primary Endpoint

The aim of this trial is to reduce neuropsychologic impairment in tuberous sclerosis patients by restoring disrupted mTOR signaling by early preventive addition of the mTOR inhibitor sirolimus to SOC. The primary endpoint is the **neuropsychologic outcome at 24 months of age assessed by rater blinded neuropsychologic testing measured by the cognitive scale on the Bayley Scales of Infant and Toddler Development III (BSID-III) compared with SOC alone.**

2.2 Secondary Objectives and Endpoints

2.2.1 Main Trial

The secondary objectives are adapted to the purpose of this study from the EPISTOP trial, a phase IIb study evaluating the impact of early versus late preventive treatment with vigabatrin on epilepsy in infants with TSC at 2 years of age [14] and will be compared between intervention and control groups:

1. Neuropsychologic outcome at 12 months of age assessed by rater blinded neuropsychologic testing using the cognitive scale on the BSID-III in both groups. Cognitive impairment will be defined as BSID-III cognitive scale score <70 similar to the EPISTOP trial [14].
2. Adaptive behaviour assessed by the Vineland Adaptive Behaviour Scales (VABS-3; digital) at the age of 12 months and 24 months.
3. Evidence for autism spectrum disorder measured at 12 months and 24 months of age by the Autism Diagnostic Observation Schedule (ADOS-2). Suspicion of autism spectrum disorder will be defined as ADOS > 12, similar to the EPISTOP trial [14].
4. Evidence for autism spectrum disorder measured by the Modified Checklist for Autism in Toddlers revised (M-CHAT-R/F).
5. TSC-associated Neuropsychiatric Disorders (TAND) severity assessed by the TAND-L Checklist (German version) at 12 and 24 months.
6. Assessment of seizure frequency and the occurrence/severity of Infantile Spasm measured by seizure diaries, caregiver questionnaires and EEG recordings.
7. Reduction of number and size of cardiac rhabdomyoma and arrhythmia (if present).
8. Reduction of cerebral tumor number and size on cMRI (if present).
9. Adverse events (AE), serious adverse events (SAE) and Adverse Events of Special Interest (AESI), assessed by the *Common Terminology Criteria of Adverse Events (CTCAE, Version 5.0)*.
10. Assessment of laboratory data and vital signs.
11. Assessment of EEG recordings for the occurrence of hypsarrhythmia and epileptiform discharges.
12. Renal changes in abdominal sonography: Volume and size of angiomyolipomas (if present), kidney size, renal pelvis dilation, echogenicity.

2.2.2 Accompanying Research

Several accompanying studies will evaluate:

1. Genetic profiling of patient-derived biomaterial to identify predictors for treatment response and TSC-associated complications.
2. Assessment of response to vaccinations under treatment with IMP compared to SOC.
3. Neuropsychological follow-up of children with tuberous sclerosis treated preventively with mTOR inhibitors at 60 months of age measured by the Vineland Adaptive Behaviour Scales (VABS-3; digital), the Social Communication Questionnaire (SCQ, German version), the TAND-L Checklist (German version) and Health-related quality of life measured by the Quality of Life in Childhood Epilepsy (QOLCE) questionnaire.

<u>Project 1. Genetic profiling approach to identify individual predictors for treatment response and TSC-associated complications</u>

TSC is highly variable in age at onset, disease severity, presence of core symptoms and occurrence of TSC-associated complications, even for patients of the same genotype [52]. Differences in genetic modifier genes and gene expression signatures (transcriptomics) may account for this heterogeneity. Analyses of genetic and transcriptomic profiles have been successfully used to identify risk profiles for TSC-associated cerebral and renal malformations [53, 54]. By using an unbiased genomic/transcriptomic approach, we aim to establish personalized disease profiles and identify predictors for individual trajectories and treatment responses. Ultimately, these results hold the potential to inform on patient stratification and tailoring of personalized treatment approaches.

Possible applications to identify subgroups are:

1. Genomics: Exome/Genome sequencing to identify shared genetic modifiers predisposing for/protective of TSC-associated manifestations and response to the study medication.
2. Epigenetics: Analysis of methylation profiles to identify epigenetic signatures correlating with disease outcomes and longitudinal changes under therapy.
3. Transcriptomics: mRNA-Sequencing to identify up-/down-regulated genes/pathways and correlations with disease outcomes and their longitudinal changes under therapy with the study medication.

This approach can broaden the understanding of disease mechanisms and increase the accuracy of predictions.

To allow a detailed analysis of our cohort, standardized sample collection will be done longitudinally at two timepoints:

- Screening: DNA, RNA
- Visit 12 (24 months of age): DNA, RNA

Blood for DNA (~1ml) and RNA (~2,7ml), will be collected during venous blood sampling procedures necessary to determine safety and efficacy of the study medication (intervention group) or establishment of the control group (non-intervention group). Since the total amount of required blood might be too high for neonates and infants, not all studies will be conducted for each individual. Blood sampling for DNA can be delayed to a future visit if not collected at screening, since changes to the DNA are not expected to occur under therapy.

Project 2. Assessment of response to vaccinations under treatment with IMP compared to SOC

Recently, mTOR inhibitors have been shown to enhance immune function and the response to vaccination (e.g. the influenza vaccine) at doses that were relatively well tolerated in the elderly [55, 56]. The impact of mTOR inhibitors on basic immunization in children has been insufficiently studied. Measurement of vaccination titers in the treatment group compared to the control group will assess differences in vaccination response and immunization. For this purpose, the titers of vaccines applied during the first year (measles, mumps, rubella, tetanus, pertussis, varicella; hepatitis b, polio) will be assessed in both groups.

To allow a detailed analysis of our cohort, standardized sample collection will be done longitudinally at two timepoints:

- Visit 8 (12 months of age) and Visit 12 (24 months of age).

Project 3. Long-term developmental outcome at 60 months of age

As part of a long-term longitudinal follow-up assessment of study results, a follow-up survey of developmental status and indicators of the presence of autism spectrum disorder will be collected at 60 months of age (5 years) measured by the Vineland Adaptive Behaviour Scales (VABS-3; digital), the Social Communication Questionnaire (SCQ) and the TAND-L Checklist (German version). This aims at investigating the long-term effect of the study medication beyond the treatment period. In addition, signs of autism spectrum disorder can be detected at this age with a higher diagnostic certainty. By comparing subsequent outcomes to the study data from the 24-month assessment, potential predictors for long-term outcome will be identified. In addition, the impact on quality of life will be stratified based on data on the severity of TSC impairment (cognitive development, seizure burden, autism spectrum disorder) and measured by the quality of life in childhood epilepsy (QOLCE; caregiver-report for aged 2- < 11) [57]. As part of the consent for this accompanying study, guardians are asked for their consent to store their contact details until the time of the follow-up survey.

3. Clinical Trial Design

3.1 Overall Trial Design

The clinical trial is planned as a two-armed, randomized, observer-blind, controlled phase IIb clinical trial for children <4 months of age at inclusion.

The treatment group will receive daily oral mTOR inhibitor (sirolimus) therapy in addition to standard of care (SOC), starting within the first 4 months of life. Sirolimus will be continued until the 2nd birthday when neuropsychologic testing will be done. The control group will receive SOC treatment only. Patients randomized to the treatment group will be followed closely with mandatory study visits at screening, start of treatment (V1), titration visits V2-V4 and at an age of 3, 6, 9, 12, 15, 18, 21 and 24 months for safety measures, to adjust sirolimus dosages following PK testing according to prespecified algorithms and to ensure compliance. In case neonates receive their first dose of IMP under the age of 4 weeks (corrected gestational age <4 weeks of life), caregivers will have up to two additional telephone calls by the study personnel up to the end of the 4th week of life. These calls will take place in those weeks where no titration visit takes place. An end-of-trial visit at 25 months of age will monitor safety parameters after end of trial. Subjects randomized to the control group will be treated according to SOC. No placebo will be administered to reduce the number of laboratory examinations at screening, 6 months, 12 months, and 24 months of age to the minimum required to control for laboratory abnormalities unrelated to the study intervention. Mandatory study visits for the control group are at screening and at 3, 6, 9, 12, 15, 18, 21 and 24 months of age. The enrolment in the primary study ends with the end-of-trial visit at age 25 months. Therapy of TSC manifestations according to SOC in both groups will not be affected by the trial. Seizure diaries, use of anti-seizure medications and concomitant medications, adverse events, vital signs (blood pressure, temperature, heart rate), height, body weight, OFC, and physical / neurological exam, and developmental milestones will be assessed at every visit. EEG and abdominal sonography will be done as described in chapter 6. Neuropsychologic testing will be performed at 12 and 24 months of age.

A schematic of the trial design is shown:

Figure 3: Schematic diagram of trial

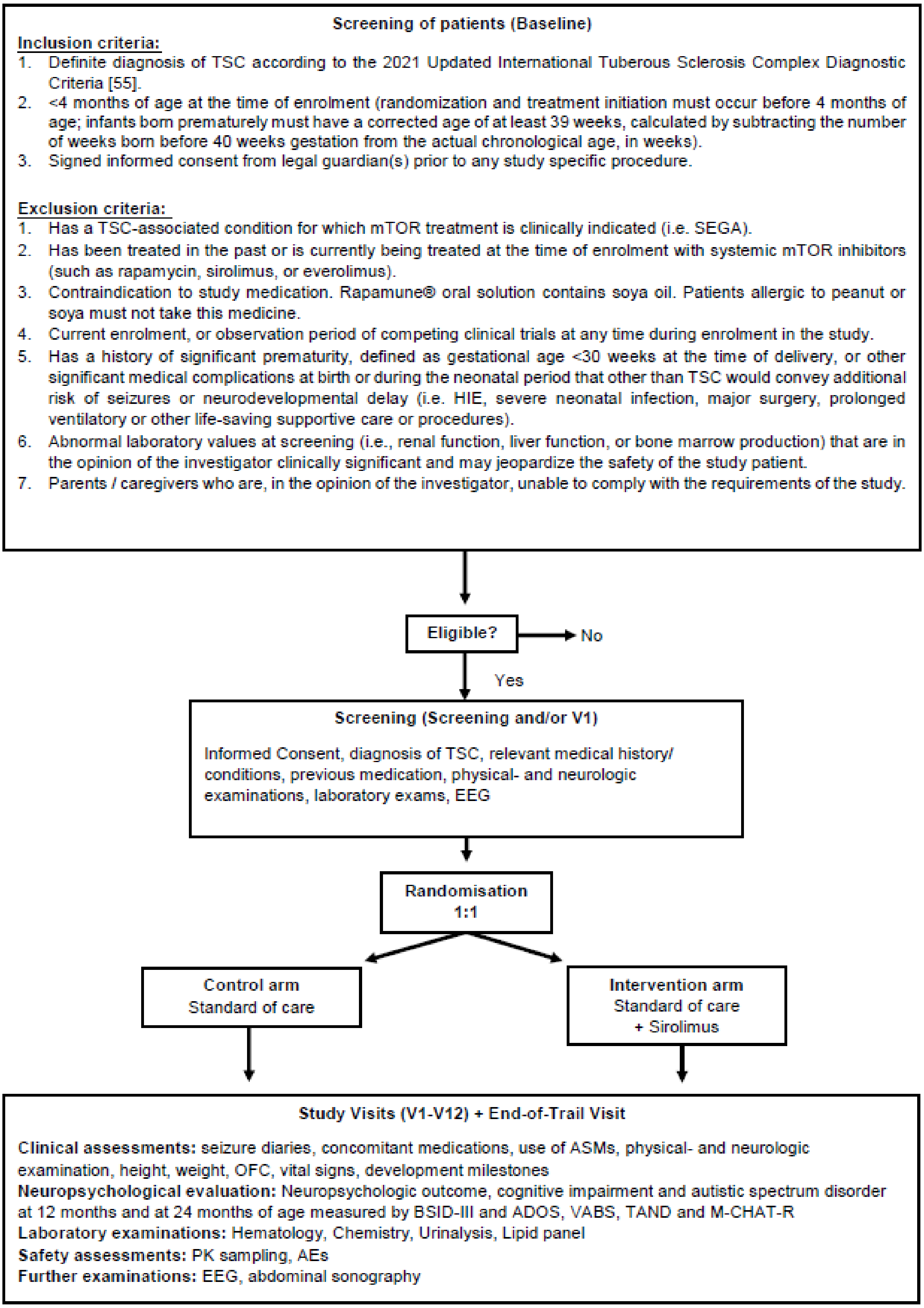

3.2 Overall Duration of the Trial

The duration of the clinical trial for each subject is scheduled to be 21-25 months, depending on the time of enrolment in the study (0 to <4 months of age), including a treatment phase until 24 months of age and a one-month follow-up.

The overall duration of the clinical trial is expected to be 82 months. Recruitment of subjects will start in Q2 2023. The actual overall and/or recruitment duration may vary. An initial safety analysis after titration phase (V4) of 5 patients in the treatment group will be done. The clinical trial end is defined as LSLV (last subject last visit).

Total clinical trial duration:	82 months
Duration of the clinical phase:	67 months
FSI (First Subject In):	Q1 2024
LSLV (Last Subject Last Visit):	Q2 2029
DBL (Data Base Lock):	Q2 2029
Statistical analysis completed:	Q3 2029
Reporting	Q4 2029

3.3 Duration of Trial Participation for each Subject

The trial consists of:

- a 2-week screening period
- a 21-24-month treatment period
- a 1-month post treatment follow-up period

In total the duration of the clinical trial for each subject is expected to be 21-25 months.

3.4 Interim Analysis

No interim analysis is planned.

3.5 Determination of End of Trial (all subjects)

The clinical trial end is defined as “last subject last visit” (LSLV).

In case of an early termination of the trial (see chapter 7.6), the date of the early termination will be the date of the end of trial.

3.6 Patient Involvement

Early-on involvement of patients in this study will improve participants’ experience in clinical trials, increase recruitment, reduce upfront protocol amendments and dropout rates and ensure investigation and communication of most important treatment benefits. During the clinically active trial, structured surveys (i.e., feedback questionnaires) and spontaneous reports of the study participants will help to decrease challenges and improve study performance. At the end of the study the patients/patient advocates are encouraged to support the dissemination of its results among patients and patient groups. The patient organization *“Tuberöse Sklerose Deutschland e.V.” (TSD),* has been involved in the planning of this study from the beginning. Prior to planning, the outline of this study has been discussed during a German wide TSD meeting with TSC

specialists, caregivers of affected patients and patient advocates. During the preparation of this proposal, regular meetings and discussions with the TSD have taken place and adjustments to the protocol have been made according to the recommendations of the scientific committee of the TSD, patient advocates and German TS clinics. Recently, the TSD awarded us with the *"TSC-Forschungspreis"* honoring our previous contribution to the field and supporting our prospective efforts regarding mTOR inhibitor treatment in children with TSC. In addition, the patient organization "*Allianz Chronischer Seltener Erkrankungen e.V." (Achse e.V.)* announced their support for our trial and committed to regular involvement in further planning. See Appendix A, letters of endorsement of the *TSD* and *Achse e.V.*.

4. Subject Selection

4.1 Number of Subjects and Sites

A total of 60 subjects will be enrolled in the clinical trial, i.e. 30 subjects per treatment group (see chapter 9.9).

The clinical trial will be multicenter study.

It is intended that the clinical trial will take place at approximately 17-20 sites.

To ensure the best possible distribution, the study has been announced on a German wide TSC meeting in February 2021. Subsequently, 16 German centres have declared their support in participating in the trial. To achieve the recruitment goal, the most promising centres were selected during pre-study visits. German-speaking centres in Austria and Switzerland were also included in the evaluation. All German TSC centres and associated clinics have announced their support in recruiting and / or referring patients to study centres.
If enrolment is delayed, additional sites may be recruited. Screening of subjects for this trial is competitive, i.e. screening for the trial will stop at all sites at the same time once a sufficient number of subjects has been included into the study. Investigators will be notified about screening completion and will then not be allowed to screen additional subjects. Subjects already in screening at this time will be allowed to continue to randomization if eligible.

If an insufficient number of study participants have been enrolled to meet the clinical endpoint, an extension of the enrolment period may occur.

4.2 General Criteria for Subject Selection

Children with at least 39 weeks of corrected gestational age until the age of <4 months, with a definite diagnosis of TSC according to the 2021 Updated International Tuberous Sclerosis Complex Diagnostic Criteria are eligible for the study [58]. A history of significant prematurity, defined as gestational age <30 weeks at the time of delivery, or other significant medical complications at birth or during the neonatal period that, apart from TSC, would convey additional risk of seizures or neurodevelopmental delay (i.e. HIE (Hypoxic Ischemic Encephalopathy), severe neonatal infection, major surgery, prolonged ventilatory or other life-saving supportive care or procedures) are excluded.

As there will be no preferences on the selection of sexes to be included, it is anticipated that the clinical trial results will give a representative sex distribution, which should reflect the natural distribution in the underlying disease.

The EPISTOP trial can be considered as a broadly comparable study, however in a European setting as detailed above (6). Considering the incidence of TSC (1:6,760 – 1:13,520 live births) in Germany and the multicenter study design, a recruitment period of 3.5 years was calculated as sufficient to answer our research question.

Children with TSC are usually not born completely healthy, but in the majority of cases show clinical signs prenatally or shortly after birth. A recent prospective epidemiologic study on 86 TSC patients in Germany, aimed at determining the incidence and diagnostic delay of TSC, reports that diagnosis is oftentimes suspected in the prenatal stage due to cardiac rhabdomyomas detected on gestational ultrasounds (22.1%), or shortly after birth due to central nervous system involvement (73.3%). Median age at possible or definite diagnosis was, thereby, reported as 6 months (range: 5 months prior to birth to 16.4 years of age) [2]. An even earlier diagnosis was reported in 2017 by Davis *et al*., who conducted a prospective study aimed at evaluating the presentation and diagnosis of TSC in infants from 5 centers in the United States. The study analyzed data of 130 infants with TSC and found that 35% presented prenatally and 41% at birth

or within the first moth of life, calculating the median time of presentation to be at birth. 74% met criteria for a definite TSC diagnosis at or within 30 days of presentation (median age at diagnosis 32 days) [30]. Thus, TSC is in most cases suspected around birth and confirmed shortly after.

4.3 Inclusion Criteria

Subjects meeting all of the following criteria will be considered for admission to the clinical trial:

1. Definite diagnosis of TSC according to the 2021 Updated International Tuberous Sclerosis Complex Diagnostic Criteria [58].
2. <4 months of age at the time of enrolment (randomization and treatment initiation must occur before 4 months of age; infants born prematurely must have a corrected age of at least 39 weeks, calculated by subtracting the number of weeks born before 40 weeks gestation from the actual chronological age, in weeks).
3. Signed informed consent from legal guardian(s) prior to any study specific procedure.

4.4 Exclusion Criteria

Subjects presenting with any of the following criteria will not be included in the clinical trial:

1. Has a TSC-associated condition for which mTOR treatment is clinically indicated (i.e. SEGA).
2. Has been treated in the past or is currently being treated at the time of enrolment with systemic mTOR inhibitors (such as rapamycin, sirolimus, or everolimus).
3. Contraindication to study medication. Rapamune® oral solution contains soya oil. Patients allergic to peanut or soya must not take this medicine.
4. Current enrolment, or observation period of competing clinical trials at any time during enrolment in the study.
5. Has a history of significant prematurity, defined as gestational age <30 weeks at the time of delivery, or other significant medical complications at birth or during the neonatal period that other than TSC would convey additional risk of seizures or neurodevelopmental delay (i.e. HIE, severe neonatal infection, major surgery, prolonged ventilatory or other life-saving supportive care or procedures).
6. Abnormal laboratory values at screening (i.e., renal function, liver function, or bone marrow production) that are in the opinion of the investigator clinically significant and may jeopardize the safety of the study patient.
7. Parents / caregivers who are, in the opinion of the investigator, unable to comply with the requirements of the study.

4.5 Lifestyle Considerations

Not applicable.

4.6 Screening and Randomization

Subjects not meeting inclusion criteria or subjects meeting exclusion criteria evident prior to enrolment are considered screening failures, which will be recorded on the screening list (see section 4.6.2)

No subject will be allowed to enrol in this clinical trial more than once.

4.6.1 Identification Numbers

All screened subjects receive a *screening number*, i.e. a consecutive, three-digit number per site. The *patient number* consists of the *two-digit site number* and the *screening number*, e.g. 01-001.

Each number will be assigned only once. For allocation to a treatment arm see section 5.1.1. No subject will be allowed to enroll (randomized) in this clinical trial more than once.

4.6.2 Screening Failures

Screening failures are defined as subjects who consent to participate in the clinical trial but are not subsequently included. The identification number as well as the inclusion and exclusion criteria have to be recorded in the eCRF. Individuals who do not meet the criteria for participation in this trial (screening failure) may be re-screened once. Re-screened subjects should be assigned a new screening number.

Re-screening is also allowed once provided that the reasons for screening failure were reversible and have been resolved, based on investigator judgement. A subject is considered a “re-screener” if he/she was not eligible for the trial initially and is subsequently re-screened, going through the informed consent process for a second time, receiving a new unique screening number and repeating the screening period assessments.

5. Treatment

5.1 Measures to minimize Bias

5.1.1 Randomization

Within -14 days prior to study visit V1 and the time of randomization, all required elements of the screening visit will be fully completed and documented by a qualified staff member. For this purpose, informed consent must be obtained from legal guardian(s). The diagnosis of TSC must be confirmed based on the diagnostic criteria. Inclusion criteria and exclusion criteria must be reviewed and documented. Vital signs, physical examination results, EEG, abdominal sonography, and laboratory results must be complete. The results will be entered by a qualified member of the local study team with access to the eCRF. Subsequently, after internal validation, randomization is performed directly via the eCRF at the site on visit 1 and treatment will start at the same day after calculation of the starting dose. The randomization via the eCRF will trigger an email to inform the sponsor and the delegated persons by the sponsor. Eligible patients will be randomized 1:1 to interventional and control arm. Block randomization will be used to minimize the imbalance between the number of patients in each treatment group. While we are expecting no centre effects due to the highly standardized therapeutic management of TSC patients, age at inclusion might bias results. Since the primary endpoint is assessed at two years of age and age at inclusion might vary from 0 to <4 months, the total treatment duration will be different depending on the age at inclusion. Therefore, the randomization will not be stratified by centre but by age groups (0-<2 vs. 2-<4 months of age).
Blinding procedures for the groups are not applicable.

5.1.2 Blinding

Blinding of trial personnel as well as patients is not feasible due to the nature of the interventions.

However, neuropsychologic testing will be performed by a blinded external observer (by the UKHD). The tests will be performed and the questionnaires will be scored by a person not involved in the treatment at the site of investigation. He or she has no access to any information concerning treatment of the study participant. During neuropsychologic testing no other study related activities (e.g. blood sampling) will be performed. Caregivers will be instructed not to disclose their study group. Questions from caregivers concerning the study are not discussed with the observer.

EEG recordings will be sent to the coordinating investigator and be analyzed by two independent blinded raters. The raters are not involved in the care of the participants they are evaluating and have no knowledge of their treatment status.

cMRI recordings will be sent to the coordinating investigator and be analyzed by blinded raters. The raters are not involved in the care of the participants they are evaluating and have no knowledge of their treatment status.

Abdominal sonography recordings will be sent to the coordinating investigator and be analyzed by blinded raters. The raters are not involved in the care of the participants they are evaluating and have no knowledge of their treatment status.

5.1.3 Other

Selection bias:

Consecutively screened and eligible patients will be enrolled. Eligible patients will be randomized using the eCRF, which maintains allocation concealment by accepting patient inclusion before announcing the treatment.

Performance bias:

Participating study personnel will be instructed about aims and procedures of the trial during initiation visits performed by a clinical monitor (KKS).

Publication bias:

The trial will be registered in the EU database which is an ICMJE-compliant data provider. The final results will be published.

5.1.3.1 Breaking the Blind

Not applicable.

5.2 Investigational Medicinal Product (IMP)

Per Regulation 536/2014 Art. 2(5) an IMP is defined as "a medicinal product which is being tested or used as a reference, including as a placebo, in a clinical trial".

Pfizer will provide sirolimus free of charge. Pfizer will take no compensation and will have no role in data acquisition or analysis. The company will have the right to review the final manuscript and make non-binding suggestions for improvement. In case of providing sirolimus, Pfizer will further be mentioned as trial supporting entity in the acknowledgements of the potential manuscript.

Table 3: Investigational medicinal product in this trial

Product name	Active Substance	Authori-zation Status	Pharma-ceutical formulation	Strength	Storage conditions	Source	RSI
Rapamune®; ATC-Code: L04AA10	Sirolimus	Not authorized for children <18 years, see also chapter 1.3.6	oral solution	1mg/ml Sirolimus	2°C to 8°C; Protect from light	Pfizer	SmPC

The IMP is delivered in 60ml bottles.

5.2.1 Approval and Indication of IMP

Rapamune® is indicated for the prophylaxis of organ rejection in adult patients at low to moderate immunological risk, receiving a renal transplant and for the treatment of adult patients with sporadic lymphangioleiomyomatosis with moderate lung disease or declining lung function. As of now, Rapamune® has no approval for the age group and indication of this above-mentioned study population.

5.2.2 Physical, Chemical, and Pharmaceutical Properties of IMP

This information has been extracted from: IB Brochure 2015, available at participating centers, withdrawn and not updated or revised by Pfizer Inc. since 2015 (Information Pfizer GmbH) [59].

5.2.2.1 Molecular Structure and Chemical Name

The chemical name of sirolimus is:

(3S,6R,7E,9R,10R,12R,14S,15E,17E,19E,21S,23S,26R,27R,34aS)-

9,10,12,13,14,21,22,23,24,25,26,27,32,33,34,34a-hexadecahydro-9,27-dihydroxy-3-[(1R)-2-

[(1S,3R,4R)-4-hydroxy-3-methoxycyclohexyl]-1-methylethyl]-10,21-dimethoxy-

6,8,12,14,20,26-hexamethyl-23,27-epoxy-3H-pyrido[2,1-c][1,4] oxaazacyclohentriacontine-

1,5,11,28,29 (4H,6H,31H)-pentone.

The chemical structure of sirolimus is:

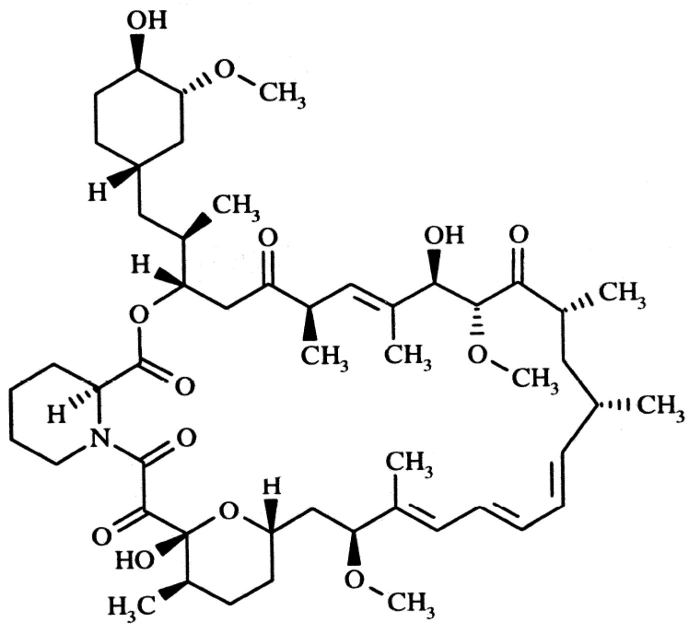

5.2.2.2 Physical and Chemical Properties

Sirolimus is a member of the Biopharmaceutics Classification System class 2 showing high permeability and low solubility [60].

Molecular formula: C51H79NO13

Molecular weight: 914.2

Appearance: White to off-white powder

Solubility: Low solubility in water (about 1.0 µg/mL); highly soluble in

benzyl alcohol, chloroform, acetone, and acetonitrile

5.2.2.3 Nonclinical Pharmacology

"Sirolimus (rapamycin or Rapamune®), a naturally occurring macrocyclic lactone, is a potent immunosuppressive agent. It has been demonstrated to prolong graft survival in many animal models of transplantation, ranging from rodents to primates, and for both heterotopic and orthotopic organ grafting, bone marrow transplantation, and islet cell grafting. Whole blood sirolimus concentrations between 5 and 60 ng/mL support maximum prolongation of allografts in the rabbit, dog, pig, and rat without significant side effects. In addition, sirolimus is highly effective in preventing the onset and severity of disease in several animal models of autoimmune diseases, such as insulin-dependent diabetes mellitus (IDDM), systemic lupus erythematosus (SLE), and arthritis. Sirolimus inhibits balloon catheter injury-induced arterial intimal thickening in the rat, because of its inhibitory effects on smooth muscle proliferation. The results of preclinical studies suggest that, in addition to organ transplantation, sirolimus has potential for clinical use in autoimmune diseases and in hyperproliferative disorders, such as vascular disease and restenosis. […] Sirolimus binds to FKBP12, in contrast to tacrolimus, the sirolimus: FKBP complex has no effect on calcineurin activity. Rather, this complex binds to a specific cell cycle regulatory protein called the mammalian target of rapamycin (mTOR) and inhibits its activation. The inhibition of mTOR suppresses cytokine-driven (IL-2, IL-4, IL-7 and IL-15) T-cell proliferation, inhibiting the progression from G1 to the S phase of the cell cycle. mTOR is a key regulatory kinase and its inhibition by sirolimus has several related effects. These include 1) inhibition of translation of a family of messenger RNAs (mRNAs) that code for proteins essential for cell cycle progression; 2) inhibition of IL-2-induced transcription of proliferating cell nuclear antigen (PCNA) that is essential for DNA replication; 3) blocking of CD28-mediated sustained upregulation of IL-2 transcription in T cells; and 4) inhibition of the kinase activity of the cdk4/cyclin D and cdk2/cyclin E complexes, causing decreased synthesis of the cell cycle proteins cdc2 and cyclin A essential for cell cycle progression." [59]

5.2.2.4 Nonclinical Pharmacokinetics and Product Metabolism

"After oral (PO) administration, the bioavailability of sirolimus was low in rats (1.9%) and monkeys (3.7%) because of significant first-pass metabolism by liver and intestinal wall, as well as poor absorption. The pharmacokinetics (PK) of sirolimus were generally linear, except at the highest toxicological dosages, and no unexpected accumulation of sirolimus occurred following chronic PO administration. After intravenous (IV) administration, the sirolimus clearance (CL) and volume of distribution at steady state (Vss) in whole blood (WB) were lower than the corresponding values in plasma, as a result of sirolimus distribution into red blood cells. Species differences in WB/plasma distribution were observed, with WB/plasma ratios following the order human ≥ monkey > pig ≥ rabbit > rat > mouse. In all species, sirolimus was extensively metabolized and the metabolites were primarily excreted via the bile into the feces. Circulating metabolites retained 30% or less of the total potency of sirolimus in the thymocyte proliferation assay. Cytochrome P450 (CYP) 3A was identified as the major CYP responsible for the metabolism of sirolimus, and there was no evidence that sirolimus causes induction of rat hepatic CYP enzymes. In vitro, sirolimus is a competitive and time-dependent inhibitor of CYP3A4/5. Urinary excretion is minor, indicating that renal dysfunction would have little effect on elimination." [59]

5.2.2.5 Clinical Pharmacokinetics and Product Metabolism

"The PK parameters of sirolimus have been well characterized in phase 1, phase 2, phase 3, and postmarketing studies involving healthy subjects and patients after administration of single, or multiple doses of IV or oral administration of either solution or tablets. Sirolimus has relatively low bioavailability, a large volume of distribution (12±8 L/kg), blood to plasma ratio of 36, and is extensively bound to human plasma proteins (92%). It is slowly eliminated from the body with a half-life of 62±16 hours in patients with renal transplants receiving concomitant CsA. Sirolimus is

a substrate of both CYP3A4 and P-gp, so administration of potent inhibitors or inducers of either enzyme will cause changes in the PK of sirolimus. Population PK analyses have not shown changes in PK parameters related to old age, race or ethnicity, size, or sex. No change in clearance was observed in subjects with impaired renal function, but clearance was decreased by 32% in subjects with moderate hepatic impairment and by 69% in subjects with severe hepatic impairment compared with subjects with normal hepatic function." [59] Within-patient fluctuations in sirolimus concentrations associated with intercurrent infection and with changes in diet are reported [61]. For more information on age-dependent pharmacokinetics, please refer to chapter 5.5.

5.2.2.6 Toxicology

"In repeated-dose toxicity studies in rats and monkeys, the majority of compound-related findings that occurred were similar to those seen for other compounds of this class, such as CsA and tacrolimus, or were secondary to long-term immunosuppression. However, certain toxicities observed with CsA and tacrolimus, notably neurotoxicity and nephrotoxicity, are not seen with sirolimus. It is hypothesized that these toxicities are mediated through the ability of CsA and tacrolimus, but not sirolimus, to inhibit the phosphatase activity of calcineurin. Most of the effects seen in nonclinical studies have not been observed in controlled clinical studies in which sirolimus was administered under the proposed therapeutic regimen. In nonclinical studies, the sirolimus-induced decreases in testosterone levels were considered likely explanations for the effects on male reproductive organs (decreased weights, testicular atrophy and degeneration, and decreased sperm counts) and on bone (osteopenia). Decreases in testosterone levels (males) and bone density have not been seen in renal transplant patients given sirolimus. When sirolimus was administered in combination with CsA, increased toxicities were observed: renal basophilia; pancreatic islet cell vacuolation, and associated hyperglycemia; thymic and testicular tubular atrophy; myocardial degeneration and accumulation of pulmonary alveolar macrophages; and fetal mortality and development. The increased severity of these effects was attributed to the significant increases in exposure levels of each compound and associated biological activity when the compounds were given in combination. In a phototoxicity study in rabbits given sirolimus, there was no evidence that exposure to ultraviolet (UV) light induced a photo-mediated toxicologic response to sirolimus; furthermore, there was no melanin binding to the pigmented tissues of rats. Sirolimus is neither a mutagen nor a clastogen and does not pose a genotoxic risk to humans.
In carcinogenicity studies, sirolimus resulted in increased incidences of lymphoma in mice, hepatocellular tumors in male mice, granulocytic leukemia in female mice, and testicular interstitial cell adenoma in male rats. The results of genotoxicity tests on sirolimus were negative, and each of these neoplasms can be related to immunosuppression. Therefore, these neoplasms are considered to be secondary to the pharmacologic effects of sirolimus and to be nongenotoxic in origin. The increased risk of lymphoma, and of other malignancies is a well-known complication of immunosuppression in transplant patients. Sirolimus does not appear to further increase this risk based upon the clinical information available to date. Although sirolimus is not considered to pose a teratogenic risk, it is not recommended for use in pregnant women because of its embryo/fetal toxicity.
Overall, based on the results of the toxicity and drug metabolism studies, sirolimus does not appear to pose an increased risk for human use under the proposed therapeutic regimen compared with that of other marketed immunosuppressive agents." [59]

5.2.2.7 Excipients with known effect

The IMP Rapamune® contains up to 25 mg ethanol and 350 mg propylene glycol (PG; E1520) per ml. In infants and neonates, both excipients are known to have potential adverse effects in case of intoxication such as hypoglycemia, acidosis, tachycardia, hypothermia, hyporesponsiveness, and disorders of consciousness (ethanol) as well as CNS toxicity, hyperosmolarity, hemolysis, cardiac arrhythmia, seizures, agitation, and lactic acidosis (PG) [62, 63]. Given the male 50th percentile, neonates will be exposed to approximately 0.8 mg/kg/d ethanol and 11 mg/kg/d PG following the herein depicted dosing scheme. Both substances are frequently found in oral applications of drugs used in neonatological and pediatric units but have to be critically applied in neonates regarding the reduced capacity of the two predominantly metabolizing enzymes, especially alcohol dehydrogenase, and catalase [63-66]. In case of ethanol, the European Medicines Agency (EMA) states the lacking information on the effects on long-term exposure to low levels of ethanol on child health and development (EMA/CHMP/43486/2018 Corr.1). *Pandya et al.* conducted a study in preterm infants, in which blood ethanol levels of 49 neonates were measured under treatment with drugs containing ethanol as an excipient [64]. On average, the exposure was 1.1-11.8 mg ethanol/kg/d, up to 15-fold daily amounts compared to the regimen of this study, that did not result in significantly elevated blood ethanol levels compared to neonates without any medication. Of note, ethanol-exposed neonates showed elevated concentrations of the toxic ethanol metabolite acetaldehyde, with yet unknown clinical significance. The authors concluded that drugs with 0.3–7.5% ethanol content do not result in toxicologically relevant systemic blood concentrations of ethanol of preterm infants, compared to 3 vol% ethanol content of the IMP [64]. In this study, neonates will be approximately exposed to 11 mg/kg/d PG by IMP application. Due to studies showing low body clearance of PG in this age group and further potential sources of PG on neonatology units [66-69], the EMA sets the safety limit to 1 mg/kg/d (EMA/175205/2014). Furthermore, the co-administration of ethanol and PG might lead to additive effects as both substances are metabolized by the same enzyme, alcohol dehydrogenase [63]. In contrast, in the first weeks of life, an increasing metabolization of both excipients, as a continuum, can be expected regarding age-dependent alcohol dehydrogenase enzyme capacities [65, 70]. In case of PG, this is reflected by extension of the reference value to 50 mg/kg/d in children >1 month of age (EMA/CHMP/704195/2013) [71, 72]. Due to the IMP formulation, ethanol and PG exposures are not avoidable within the scope of this study, also for infants <4 weeks of age. Although this vulnerable age group will likely represent only a small fraction of probands, it is nevertheless expected to benefit in particular as early treatment is likely crucial for neuropsychological outcome. For risk minimization, several measures will therefore be taken: 1.) Excipient exposure is minimized by a low-dose regimen that includes dose fractioning to twice daily to avoid toxic peak concentrations. In the neonatal period, dose fractioning will be maintained for daily volumes <0.2ml to address the reduced excipient metabolization in this age group. For infants ≥4 weeks, minimal single volumes of 0.1ml will be applied. If necessary, switching to a once-daily regimen is allowed, given the improved excipient metabolization with a 50-fold higher safety limit of PG exposure. 2.) Caregivers are sensitized to elimination of further ethanol and PG sources wherever possible. This includes concomitant medications such as phenobarbital, lorazepam, phenytoine, and further drugs [63, 73]. 3.) Neonates will receive a close risk monitoring for accumulation and systemic toxicity with additional visits. Caregivers of the neonates who receive their first dose of IMP under the age of 4 weeks (corrected gestational age <4 weeks of life) will have up to two additional telephone calls by the study personnel up to the end of the 4th week of life. These calls will take place in those weeks (+/-2 days) where no titration visit takes place. Telephone calls instead of onsite-visits will be performed to reduce stress to the neonates wherever possible. Subinvestigators and caregivers will be sensitized to the above stated clinical signs of intoxication and neurologic, cardiovascular, and respiratory status will be conducted at any visit. Caregivers will be advised to report any clinical signs suspicious for intoxication to the study personnel whenever they occur. If any signs occur that require an on-site

visit, the study staff will act accordingly. At the discretion of the investigator, a blood sample could be taken for a safety laboratory which include the blood alcohol concentration (BAC) and surrogate parameters for potential PG intoxication including electrolytes, osmolality, creatinine, urea, pH, lactate, bicarbonate, anion gap, and the calculated osmol gap as proposed by *Barnes et al.* [74]. For further reference, the currently recruiting STOP2 trial (ClinicalTrials.gov: NCT05104983) conducted by *Krueger et al.* includes probands from 1 day to 6 months of age and did not report any SAE with suspicion of intoxication or leading to study dropout so far.

5.2.3 IMP Supplies

The IMP will be provided free of charge by Pfizer to the pharmacy of the University Hospital Heidelberg (central pharmacy). The appropriate number of drug bottles will be dispensed to the sites by the central pharmacy.

The trial site will be equipped with one drug bottle Rapamune® by the pharmacy of the University Hospital Heidelberg for the first scheduled screening visit.

Re-supplies of the IMP have to be obtained by sending an order form to the central pharmacy. The required amount of investigational medicinal product will be shipped to the study sites by the pharmacy.
The trial centres have to keep records of the trial medication on a patient-specific drug accountability log and a site-specific inventory log on receipt, distribution, use, return, loss, or other disposition of medication in alignment to the receipts of drug shipment. Above-mentioned logs will be approved and provided by the sponsor. The documents contain information about dates, quantities, batches or code / serial numbers and patient identification codes.

At each study visit the caregivers have to return the used bottles. At the trial site, a responsible member of the study team will assess the returned amount of study medication by measuring the filling level of the used medication bottle using a scale with a quantity indication in ml provided by the sponsor.

Trial medication that may no longer be used (e.g. excess temperature, expired) may be destroyed before the end of trial after verified and released by the monitor. At the end of the trial, all unused and expired trial medication has to be destroyed at trial site. This must be documented on the respective logs. If the destruction at trial site is not possible, the medication can be sent to the sponsor for destruction. The trial site will receive a report for the ISF.

5.2.4 IMP Packaging and Labelling

IMP is an authorised investigational medicinal product (Rapamune®) provided free of charge by Pfizer (see 5.2.1) which will be labelled (immediate as well as the outer packaging) by the central pharmacy of the University Hospital Heidelberg according to good manufacturing practice and in compliance with Annex VI of CTR.
Rapamune® will be distributed to the investigators by the central Pharmacy (Apotheke des Universitätsklinikums Heidelberg, Germany).

5.2.5 IMP Storage

For the shipment from the central pharmacy to the trial site, the cold chain has to be maintained.

The investigator, or an approved representative, e.g. pharmacist, will ensure that all investigational products, including any comparator and/or marketed products are stored in a secured area with controlled access under required storage conditions and in accordance with applicable regulatory requirements. Investigational medicinal product should be stored in its original container and in accordance with the label (see also

Table 3). Site systems must be capable of measuring and documenting (e.g. via a log), at least every working day the minimum and maximum temperatures for all site storage locations (as applicable, including refrigerated products). This should be captured from the time of investigational medicinal product receipt throughout the trial. Even for continuous monitoring systems, a log or site procedure that ensures active daily evaluation for excursions should be available. The operation of the temperature monitoring device should be regularly inspected to ensure it is maintained in working order.

In case of excursions from the product label storage conditions, such excursions should be reported upon discovery and the site should actively pursue options for returning the product to the storage conditions as described in the labelling, as soon as possible. Deviations from the storage requirements, including any actions taken, must be documented and reported to the sponsor. Once an excursion is identified, the investigational product must be quarantined and not used until the sponsor provides documentation of permission to use the investigational product. It will not be considered a protocol deviation if the sponsor approves the use of the investigational product after the temperature excursion. However, use of the investigational product prior to sponsor approval will be considered a protocol deviation. The sites will be provided with specific details on reporting of excursions.

Site staff will instruct subjects on the proper storage requirements for take home investigational product, how to report temperature excursions and the use of the medication (see also chapter 5.4).

5.2.6 IMP Accountability

The site must maintain adequate records documenting the receipt, use, loss, or other disposition of the investigational medicinal product supplies. To ensure adequate records, all drug supplies will be accounted for in the drug accountability inventory forms as instructed and will be monitored periodically to ensure the correct accountability of all IMP used (see also chapter 5.2.3).

5.3 Auxiliary Medicinal Product(s) (AxMPs)

The standard of care applied in this trial includes regular examinations and observation of the patients, as well as symptom-related therapies. In addition to therapeutic treatments, these may also include drug treatments, which may vary depending on the severity of the symptoms shown.

Only authorized products will be used as AxMPs and the specific use will always depend on the patients´ needs and will be done according to the investigators´ judgement at site. The most common treatments are only defined by active substance; therefore, the used products may vary from site to site.

Active Substance	Pharma-ceutical formulation	App. dosage	Attached SMPC example
Vigabatrin	Oral Granulate	50-150 mg/kg/day	Sabril® 500 mg Filmtabletten<br>Sabril® Beutel
Prednisolone	Oral Tablet	4x10 mg/kg for 14 days	Prednisolon JENAPHARM®
Tetracosactid	Suspension for i.m.Injection	40 I.U. every other day for 14 days	Synacthen Depot ®
Oxcarbazepine	Oral Suspension	20-35 mg/kg	Trileptal® Suspension
Carbamazepine	Oral Suspension	20-35 mg/kg	Tegretal® Suspension
Valproic acid	Oral Suspension<br>Microcapsules	20-30 mg/kg	Valproat-neuraxpharm<br>® 300 mg/ml<br>Orfiril long® 150/300mg
Phenobarbital	Tablet	3-5mg/kg/day	Luminal®/Luminaletten®
Propranolol	Solution for Injection	0,25-1mg/kg<br>3-4 times/day	Dociton® Injektionslösung 1 mg/ml
Propranolol	Film-coated Tablet	0,25-1mg/kg<br>3-4 times/day	Dociton® 10/40/80 mg Filmtabletten
Verapamil	Film-coated Tablet	40mg/1-2 times/day	Isoptin® mite, 40 mg, Filmtabletten
Midazolam	Buccal Suspension	Rescue medication	BUCCOLAM<br>2,5 mg / 5 mg
Diazepam	Rectal Tube	Rescue medication	Diazepam Desitin® rectal tube<br>5 / 10mg

Organ manifestations in TSC are age-dependent. Medical treatment for TSC-associated manifestations in the first two years of life are addressing mainly manifestations of the central nervous system (epilepsy) and cardiac manifestations (arrhythmia) [4]. Other organ manifestations, e.g. renal manifestations usually occur later in life and are not primarily treated with medical treatment apart from mTOR inhibitors [7], [29]. Treatment strategies as part of SoC involves mainly diagnostic procedures and non-medical interventions (e.g. physical therapy, occupational therapy, cardiac surgery).

AxMPs in infants with TSC that are commonly used in infancy and early childhood are antiseizure medications, including rescue medications and much less frequent anti-arrhythmia medications for cardiac complications.

So far, no medical treatment in tuberous sclerosis is known to have an impact on neuropsychologic outcome at 24 months of age. The only study addressing effects of early treatment on neuropsychologic outcome in TSC by [14] did not show significant effects. Studies comparing vigabatrin to hormonal treatment (prednisone or ACTH) or a combination of vigabatrin and hormonal treatment failed to show differences on long-term cognitive outcome [75]. It has been shown that approved antiseizure medications in this age group, such as phenobarbital have no positive effect and can be harmful to cognitive development [76].Overall, it can be assumed that antiseizure medications or other symptomatic treatment in TSC has no disease-modifying effect on neuropsychological outcome.

Antiseizure medication

Vigabatrin

Vigabatrin is an irreversible GABA transaminase inhibitor and used as first line therapy in infants with TSC presenting with infantile spasms (West syndrome). (It is also now used off-label as preventive treatment in TSC and can reduce the risk of infantile spasms [14]. Vigabatrin is not known to have interactions with sirolimus (see also chapter 5.6.1). In children preventively treated with vigabatrin no significant difference was seen regarding future development of autisms spectrum disorder or intellectual disability at 2 years [14].

Prednisolone / Adrenocorticotropic hormone

While the exact mechanism is not completely understood, hormonal treatment with prednisolone or ACTH (Synacthen) are approved and beneficial for infantile spasms (West syndrome) in non-TSC infants and in infants with TSC. Effects on cognitive outcome seem to be comparable to vigabatrin. As vigabatrin is currently the treatment of choice for infants with TSC [77], hormonal treatment is less frequently used in the study population and vigabatrin is recommended for SoC. No relevant pharmacological interactions with sirolimus are known.

If ACTH is used in the treatment group, antibiotic prophylaxis, e.g. with cotrimoxazole, should be considered.

Oxcarbazepine and Carbamazepine

Carbamazepine is approved for the treatment of focal seizures and oxcarbazepine as adjunct treatment for focal seizures in the study population. Both are sodium channel blockers. No positive effects on neuropsychological outcome are expected. As CYP3A4 inducers, pharmacologic interactions with sirolimus should be respected. Significant clinical interactions regarding primary study outcome are not expected.

Valproate (valproic acid)

Valproate is a GABAergic drug, that is approved for focal and generalized seizures and as adjunct treatment in all epilepsy syndromes. Valproate, similar to carbamazepine can have effects on neuropsychological outcome, however, the effects on cognition have been shown to usually mild [78].

Phenobarbital

Phenobarbital is approved as treatment for early and neonatal seizures. As an inducer of CYP3A4, it may increase the metabolism of sirolimus and decrease sirolimus blood levels, warranting additional drug monitoring or sirolimus trough levels. Regarding neuropsychological outcome, in clinical practice, novel antiseizure medications (e.g. not approved levetiracetame) are preferred over phenobarbital as first line treatment in infancy, given the known unfavourable effects of phenobarbital on cognitive outcome [76].

Antiarrhythmia / treatments for cardiac manifestations

Propranolol

Propranolol is a beta-adrenergic receptor blocker. It is not known to have any effect on general child development and neuropsychological outcome in TSC. Possible effects on the nervous system are not likely to be relevant to the study outcome (vertigo, temporary confusion). It is approved as antiarrhythmic treatment and might be used in infants with TSC according to treating cardiologists. Propranolol can have effects on fat metabolism and also on other laboratory results (see chapter 5.6.1).

Verapamil

Verapamil is a calcium-channel blocker used for arrhythmia. While verapamil can have central nervous system effects, no studies assessed systematically effects on development or epilepsy. Regarding interactions, sirolimus levels can be increased and should be monitored, appropriate dose reductions of both medicinal products should be considered (see chapter 5.6.1).

Rescue medications

Midazolam (buccally, from 3 months) and rectal diazepam are approved rescue medications for prolonged epileptic seizures. They have anti-seizure effects and they can have sedative effects. Recurrent or chronic use should be avoided, as negative effects on neuropsychological outcome similar to phenobarbital can be expected.

5.3.1 AxMP Supplies/Labelling/Storage

All used AxMPs will be used at discretion and judgment of the responsible investigator at each site and with the products available at site. The sponsor will not provide any of these products. As all products are authorized and used according to clinical routine, no labeling is necessary.

All products will be stored at clinical site according to the applicable storage conditions in the SmPC and the sites local policy.

No drug account will be done for any AxMP. The use of AxMPs will be documented in the eCRF.

5.4 Administration

After randomization is completed, the correct starting dose of the IMP in the treatment group is determined by the study personnel by entering the patient's age in months, height in centimetres, and weight in kilograms into the eCRF (drug account). The first dose will be administered at the center. Caregivers of individuals in the treatment group will then receive a sufficient amount of the investigational drug as well as a sufficient number of oral syringes (Amber Barrel 1ml, product

code: 1ml, ABLDS-PFCO-1-EU; 0,01ml-scale) which are light-protected and adapter for the bottles (adapter: Size 1 (12 – 15 mm), ENFIT® Bottle Adapter, BAISO1-S).

Sirolimus will be dispensed by study center personnel on an outpatient basis at start of treatment visit (V1), if necessary, on titration visits (V2-V4) and at each study visit in a three-months interval at 3, 6, 9, 12, 15, 18, and 21 months of age. Caregivers will be provided with an adequate supply for self-administration at home, with exception of the days when PK sampling will be performed, and sirolimus will be administered at the site after blood sample collection. Caregivers will be instructed by the trained study personnel to administer sirolimus exactly as prescribed. The first dose will be administered at the trial center. All dosages prescribed and all dose changes during the study will be recorded on the Dosage Administration Record in the eCRF. The medication administered, specifics of administration, AEs, and comedications are entered into the patient diary by the caregivers. Sirolimus oral solution will be administered in an oral syringe before feeding or meal. Rapamune® oral solution is not allowed to be diluted with grapefruit juice or any other liquids. Caregivers will administer the predetermined amount of clinical trial medication twice daily: in the morning directly before breakfast (e.g. breastfeeding or other food/liquid intake) and in the evening directly before dinner (e.g. breastfeeding or another food/liquid intake). The dosing regimen will differentiate between the two age groups of neonates <4 weeks and infants ≥4 weeks in order to address the reduced metabolization capacity of IMP excipients in the first month of life (see also section “5.2.2.7 Excipients with known effect”). For neonates <4 weeks, the IMP application twice daily will be maintained even for single volumes <0.1 ml. A possibly reduced dosing accuracy for these small volumes will therefore be tolerated in this age group to avoid toxic peak concentrations of IMP excipients. For infants ≥4 weeks receiving a daily dose of <0.2 ml, administration is reduced to once daily in the morning. For infants ≥4 weeks receiving daily volumes <0.1 ml in the morning the administration will be every two days with the double daily dose to ensure dosing accuracy. The application regimen will be switched to a twice-daily regimen by the study team at the earliest timepoint possible. A new oral syringe should be used for each administration. Caregivers will be instructed to ensure the entire dose is administered. In the event of spitting out the study medication without any portion having already been swallowed, the full dose should be re-administered. This should not be done more than once. In case of vomiting, no attempt should be made to replace the vomited dose. If the administration of the medication is missed, it can be carried out up to the next scheduled administration, in concordance with the SmPC. Missed doses should be documented and discussed with the study team at the next visit to work out solutions. On the days of PK sampling, patients should not be administered the sirolimus morning dose until after blood work is drawn so that an accurate trough level can be obtained. On days of scheduled visits, patients should bring their daily dose of sirolimus into the clinic for administration after blood work is drawn. Sirolimus 12h-trough levels will be measured when IMP is administered twice daily, 24h-trough levels in cases of 24h or 48h intervals of administration, at every study visit. Dose adjustments to achieve target levels of 5-10 ng/ml will be made. Trough levels will be communicated to caregivers in person or by telephone by study personnel. Until adjustments are communicated, the current dose will be continued.

Caregivers will be requested to bring their unused study drug, empty bottles and partly used bottles, to the hospital at each visit. Compliance will be verified by the investigator’s staff by assessing the amount of dose administered (see also chapter 5.2.3). Families will be requested to return all unused study drug at each dispensing visit and at the end of the study. After checking the remaining quantities, bottles that have already been opened and have a sufficient shelf life (maximum 30 days after opening) can be given to the study participants again.

The investigator or other personnel allowed to dispense the IMP will be responsible for ensuring that the IMP used in the clinical trial is securely maintained as specified by the Sponsor and in accordance with applicable regulatory requirements.

The clinical trial medication must be carefully stored in accordance with manufacturer's instructions:

- The Rapamune® medicine bottles should be stored in a cool (2–8°C), dark and dry environment at the subject's home.
- After preparation in a syringe, the solution should be stored light-protected at < 25°C and no longer than 24h.
- The Rapamune® medicine bottles and prepared syringes should be stored in a secure place where they are not accessible to other children or individuals.
- It is forbidden to give Rapamune® to third parties.
- The Rapamune® medicine bottles and prepared syringes should be stored separately from other drugs. In the treatment group, caregivers will be instructed by the trained study personnel about correct dosing (see also chapter 5.4).

The administration process shall be discussed and trained in detail with the caregivers by the trial staff. The caregivers will also receive an information / training leaflet (Additional Information to Rapamune® solution -Handling, Dosage, Administration-) as part of the Informed Consent (Annex II of the IC).

5.4.1 Preparation of Medication

A syringe adapter must be inserted into the bottle until it is aligned with the top edge of the bottle. An 1ml oral syringe is inserted into the opening of the syringe adapter. Before the syringe can be filled, the plunger of the syringe must be fully inserted into the syringe. The plunger of the syringe is carefully pulled out of the syringe until the liquid level inside the syringe reaches the desired value in ml on the 0,01-syringe scale. The bottle should be turned upside down for drawing up the solution. If bubbles appear in the solution during collection, the solution should be emptied back into the bottle and the collection repeated: for a good result, the syringe should be drawn up and back 5 times to remove air bubbles in the syringe. The full content of the oral syringe should be immediately, but slowly and gently, dispensed into the mouth of the patient. Care should be taken to ensure that the entire dose is administered. If an immediate administration of the prepared dose does not take place, the application syringe must be stored no longer than 24h at <25°C (best light-protected).

5.5 Dosing

Sirolimus dosing recommendations vary among studies, depending on age of subjects and indications. Sirolimus has a narrow therapeutic window with considerable inter -individual pharmacokinetic variabilities. Most studies report median absolute doses of 2-2.5 mg daily (summarized in Overwater *et al.*, 2016, table e-1). Overwater and colleagues calculated a starting dose of 0.033 mg/kg once daily, based on the normal starting dose of 2 mg daily for an adult of >50kg body weight, initially targeting trough levels of 10-15 ng/ml, which had to be reduced to 5-10 ng/ml after the occurrence of two serious adverse events [42]. He *et al.*, by contrast utilize doses of 1mg/m^2 BSA once daily targeting trough concentrations of 2-10 ng/ml [36]. Almost all dosing recommendation for sirolimus in neonates and infants with indications other than TSC vary around 0.05-0.1 mg/kg or 0.5-1 mg/m^2 in line with our dosing scheme. The traditional fixed-dose stratification or weight-based dosing designs only control for a small part of the variable age-depended metabolization of sirolimus, frequently leading to drug exposures outside of the targeted range [79]. Developmental changes over time in neonates and infants have a major impact on the pharmacokinetic-pharmacodynamic (PK/PD) profile of mTOR inhibitors such as sirolimus (e.g. age-dependent change in CYP3A activity) [80]. Sirolimus is extensively metabolized through CYP3A4 and CYP3A5 enzymes [81, 82] and there are several in vitro studies showing age-dependent changes in each hepatic CYP3A isoform expression [83, 84]. Emoto *et al.* described

the relationship between patient's age and allometrically size-normalized sirolimus clearance estimates by a sigmoidal E_{max} model. They demonstrated the age-dependent trajectory of sirolimus clearance in neonates and infants after considering the effect of body size. They conclude that the developmental change in CYP3A expression is the most important factor for the age-dependent trajectory in neonates and infants [80]. An interesting report by Wang and colleagues used population pharmacokinetics and nonlinear mixed effect modeling to predict the optimal sirolimus dosing regimen in pediatric patients with TSC [85]. Based on data of 15 children between 1.08 and 13.95 years and designed to achieve target concentrations of 5-15 ng/ml, the authors recommend a starting dose of 0.1 mg/kg once daily for children between 5-10 kg body weight or 0.04 mg/kg twice daily for children between 5-20 kg body weight [85]. An important limitation to this study, however, is the absence of pharmacokinetic data from infants and children under 1 year of age, particularly considering the developmental differences in the activity of intestinal and hepatic drug-metabolizing enzymes and efflux transporters altering the bioavailability of drugs [86-88]. Driven by this motivation, Mizuno and colleagues used a similar prediction model to develop an age-appropriate sirolimus starting dose regimen for neonates and infants based on developmental changes in drug elimination capacity using 316 concentration measurements from 25 patients aged 0-4 years (15 of which were less than 2 years old). The trajectory of simulated sirolimus clearance increased with age. Mizuno proposed dosing regimens depending on age and body surface area and achieved target attainment of more than 75–95% across selected regimens [89]. The results are depicted in the following Figure 4 by Mizuno et al. [89].

Figure 4: Simulated sirolimus clearances compared with the estimated age-appropriate starting dosing regimens for the target range of 10–15 ng/ml

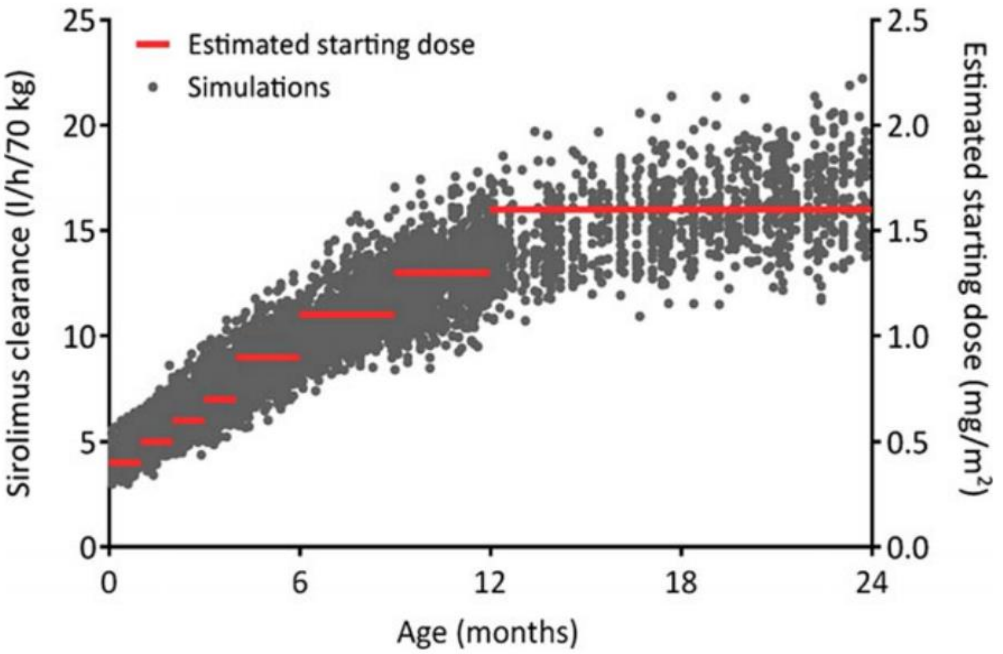

Based on the presented evidence, and in agreement with international TSC experts and our scientific advisory board, we believe that the model proposed by Mizuno *et al*. best serves this study since it includes pharmacokinetic data of infants/neonates, BSA and uses a twice daily (BID)

regimen, which was shown to reduce absolute doses [89, 90]. To reduce the occurrence of AEs we will target trough levels of 5-10 ng/ml.

Table 4: Starting daily dose of IMP

Age group (months)	Daily Dose (mg/m²/day)
**0 - <1**	**0.5**
**1 - <2**	**0.6**
**2 - <3**	**0.74**
**3 - <4**	**0.9**

In general, the daily dose is divided into two administrations. In particular, a distinct dosing regimen will be used for neonates <4 weeks addressing the reduced excipient metabolization (see also sections "5.2.2.7 Excipients with known effect" and "5.4 Administration"): For neonates <4 weeks, the BID regimen will be maintained for daily volumes <0.2 ml, even for single volumes <0.1 ml. For infants ≥4 weeks receiving a daily dose <0.2 ml, administration is reduced to once daily in the morning to ensure dosing accuracy and switched to a BID regimen by the study team at the earliest timepoint possible. For infants ≥4 weeks receiving daily volumes <0.1 ml in the morning the administration will be every two days with the double daily dose to ensure dosing accuracy. Subjects randomized to the treatment group will receive oral sirolimus (Rapamune® oral solution) therapy. Sirolimus will be administered adapted to body surface area (BSA) with an age-dependent starting dose of **0.5 mg/m^2/day (0 - <1 months of age) to 0.9 mg/m^2/day (3 - <4 months of age)** divided BID, in addition to SOC.

The starting dose is calculated by the investigator by entering the patient's age in months, height in centimetres, and weight in kilograms into the eCRF. The system will calculate the appropriate starting dose. Rounding down on the discretion of subinvestigators for steps of 10µl will be accepted. The body surface area in m^2 will be calculated using the Gehan-George-formula: body surface area [m2] = 0.0235 x height [cm]$^{0.42246}$ x weight [kg]$^{0.51456}$ [91].

5.5.1 Maximum duration of treatment

Start of treatment at <4 months of age until 24 months of age. The maximum duration of treatment is 24 months inside the study.

5.5.2 Maximum dose allowed

After the study drug has been started at the doses listed in, subsequent dosing will be individually adjusted informed by measurements of trough levels carried out at each study visit to reach target trough levels of 5-10 ng/ml.

5.5.3 Dose modifications and interruptions

Two clinical trials in the United States (ClinicalTrials.gov: NCT05104983) and Poland (ClinicalTrials.gov: NCT04987463) are currently evaluating whether infantile-onset sirolimus

treatment can prevent/delay seizure onset in TSC. The following scheme for dose adjustments was established in collaboration with the study teams of the currently active trials in the US and Poland and is informed by previous experience in the use of sirolimus in this age group. No relevant AEs or overdosing occurred in the above-mentioned studies to date (as of 12/2022). Along these lines, in a previous study of patients with TSC–related seizures (EXIST-3 study), the investigators report that even twofold increases of normal everolimus blood levels did not significantly increase the risk of stomatitis or infections [92].

Table 5: Sirolimus dose adjustment

Sirolimus trough levels (PK in ng/ml)	Required dose adjustment
**PK < 5**	double all subsequent doses; additional telephone contact of trial site with caregiver(s) after 7 days +/- 2 days; repeat levels in 2 weeks
**5 ≤ PK ≤10**	no adjustment
**10 < PK ≤ 15**	if no clinically significant AE or laboratory abnormalities occur, no dose adjustments; repeat levels in 2 weeks
**15 < PK ≤ 20**	reduce all subsequent doses to 75%; repeat levels in 2 weeks
**PK > 20**	skip next dose, then reduce all subsequent doses to 50%; repeat levels in 2 weeks

<u>Legal guardian(s´) should be informed by telephone or e-mail of dose adjustment by trial site as soon as laboratory results are available at trial site. Legal guardian(s´) should be advised to contact the trial site if they did not receive a phone call or e-mail within 4 days after the last study visit.</u>

<u>Vaccinations:</u>

Sirolimus may affect the response to vaccinations. Sirolimus should be paused 14 days before until 14 days after receiving a live vaccine. The SmPC of sirolimus recommends avoiding live vaccinations during therapy. Individual risk/ benefits should be discussed with infectious disease specialists.

<u>Surgical interventions:</u>

Treatment with sirolimus may affect wound healing. We recommend pausing treatment 14 days before until 14 days after elective surgical interventions. For non-elective interventions, therapy should also be paused until 14 days after the procedure.

Table 6: Sirolimus dose modifications for toxicities

This section is adapted from [23].

Non-infectious pneumonitis grade 2	Interrupt study drug administration until resolution to ≤ grade 1. If resolution occurs within ≤ 4 weeks, study drug should be re-started at the dose level prior to interruption. If resolution takes > 4 weeks, or if event recurs, hold study drug until recovery to ≤ grade 1 and reduce subsequent doses to 50%.
Non-infectious pneumonitis grade 3	Interrupt study drug administration until resolution to ≤ grade 1. If resolution occurs ≤ 4 weeks reduce subsequent doses to 50%. If resolution takes > 4 weeks, discontinue study drug.
Non-infectious pneumonitis grade 4	Discontinue study drug
Intolerable grade 2 and 3 mucositis	Interrupt study drug administration until resolution to ≤ grade 1. If resolution occurs within ≤ 7 days, study drug should be re-started at the dose level prior to interruption. If resolution takes > 7 days, or if event recurs within 28 days, hold study drug until recovery to ≤ grade 1 and reduce subsequent doses to 50%.
AST or ALT elevation grade 1 (> ULN - 3.0 x ULN) or grade 2 (> 3.0 - 5.0 x ULN)	Maintain current dose level
AST or ALT elevation grade 3 (> 5.0 - 20.0 x ULN)	Interrupt study drug administration until resolution to ≤ grade 1 (or ≤ grade 2 if screening values were within the range of grade 2). If resolution occurs ≤ 7 days, study drug should be re-started at the dose level prior to interruption. If resolution takes > 7 days, or if event recurs within 28 days, hold study drug until recovery to ≤ grade 1 and reduce subsequent doses to 50%.
AST or ALT elevation grade 4 (> 20 x ULN)<br>Recurrence of grade 4 after dose reduction or toxicity requiring study drug interruption for > 28 days	Interrupt study drug administration until resolution to ≤ grade 1 (or ≤ grade 2 if screening values were within the range of grade 2). If resolution occurs ≤ 7 days reduce subsequent doses to 50%. If resolution takes > 7 days, discontinue study drug.
Any other grade 4	Hold study drug until recovery to ≤ grade 1 or screening value. Reduce subsequent doses to 50%.
Recurrence of grade 4 after dose reduction	Discontinue study drug
Grade 3 or 4 clinical liver failure (asterixis or encephalopathy/coma)	Discontinue study drug
Any non-hematologic toxicity requiring study drug interruption for > 28 days	Discontinue study drug
Grade 2 thrombocytopenia	No action
Grade 3 thrombocytopenia	Interrupt study drug until resolution to ≤ grade 1 or screening level. If resolution occurs ≤ 7 days, reintroduce study drug at the dose level prior to interruption. If resolution occurs > 7 days, or event reoccurs within 28 days, reduce subsequent doses to 50%.
Grade 4 thrombocytopenia	Interrupt study drug until recovery to ≤ grade 1. Then reduce subsequent doses to 50%.
Grade 2 neutropenia or anaemia	No action
Grade 3 neutropenia or anaemia	Interrupt study drug until resolution to ≤ grade 1 or screening level. If resolution occurs ≤ 7 days, reintroduce study drug at the dose level prior to interruption. If resolution occurs > 7 days, or event reoccurs within 28 days, reduce subsequent doses to 50%.
Grade 4 neutropenia or anaemia	Interrupt study drug until recovery to ≤ grade 1. Then reduce subsequent doses to 50%.
Febrile neutropenia	Interrupt study drug until resolution to ≤ grade 1 or screening level. If resolution occurs ≤ 7 days, reintroduce study drug at the dose level prior to interruption. If

	resolution occurs > 7 days, or event reoccurs within 28 days, reduce subsequent doses to 50%.
Any hematologic toxicity requiring study drug interruption for > 28 days	Discontinue study drug

Re-initiation of treatment with the IMP will be done under close and appropriate clinical and/or laboratory monitoring if according to the investigator's medical judgment the concerned event is unlikely to be related to the IMP and if the selection criteria for the trial are still met. For all temporary treatment discontinuations, duration must be recorded by the investigator in the appropriate pages of the eCRF.

5.5.4 Management of specific toxicity

This section extracted and adapted from [23].

5.5.4.1 Management of infections

Sirolimus has immunosuppressive properties and may predispose patients to bacterial, fungal, viral or protozoal infections, including infections with opportunistic pathogens.

Investigators and caregivers should be aware of the increased risk of infection during sirolimus treatment. Pre-existing infections should be treated prior to starting with sirolimus. While taking sirolimus, symptoms and signs of infection should be monitored; if a diagnosis of infection is made, appropriate treatment should be given and interruption or discontinuation of sirolimus should be considered (see section 5.5.3, Table *6*, section 7).

If ACTH for infantile spasms is used in the treatment group, antibiotic prophylaxis, e.g. with cotrimoxazole, should be considered.

If a diagnosis of invasive systemic fungal infection is made, discontinue sirolimus and treat with appropriate antifungal therapy.

5.5.4.2 Management of skin toxicity

For study participants with grade 1 toxicity, no specific supportive care is needed or indicated. A rash must be reported as an AE. Patients with grade 2 or higher toxicity should be treated with age-appropriate supportive measures at the discretion of the investigator.

5.5.4.3 Management of stomatitis / oral mucositis / mouth ulcers

Caregivers should be instructed to report the first onset of buccal mucosa irritation/reddening to their study team immediately.

Stomatitis/oral mucositis/mouth ulcers due to the study drug should be treated using local supportive care. Interruption or discontinuation of sirolimus should be considered (see section 5.5.3, Table *6*, section 7).

5.5.4.4 Management of diarrhea

Appearance of grade 1-2 diarrhea attributed to study drug toxicity may be treated with supportive care at the earliest onset.

5.5.4.5 Management of non-infectious pneumonitis

Non-infectious pneumonitis is a class effect of rapamycin derivatives. Cases of interstitial lung disease (including pneumonitis, and infrequently bronchiolitis obliterans with organizing

pneumonia [BOOP] and pulmonary fibrosis), with no identified infectious etiology have occurred in adult patients receiving immunosuppressive regimens including sirolimus. Some of these have been severe and on rare occasions, a fatal outcome was observed. In some cases, the interstitial lung disease has resolved upon discontinuation or dose reduction of sirolimus. These risks may increase with higher sirolimus trough levels. Individuals participating in this trial will be routinely assessed for the occurrence of new or changes in existing pulmonary symptoms consistent with lung toxicity. Consultation with a pulmonologist is recommended for any case of pneumonitis that develops during the study.

A diagnosis of non-infectious pneumonitis should be considered in patients presenting with non-specific respiratory signs and symptoms such as hypoxia, pleural effusion, cough, or dyspnea, and in whom infectious, neoplastic and other non-medicinal causes have been excluded by means of appropriate investigations. Caregivers should be advised to promptly report any new or worsening respiratory symptoms.

Consider corticosteroids if infective origin is ruled out. Taper as medically indicated.

5.5.5 Subject Compliance

Compliance is measured by the amount of study medication left in the medication bottles at each study visit.

Subject compliance will be assessed at each visit during the trial and will be verified by interviewing caregivers and through accounting of returned trial medication at each visit and the patient diary kept by the caregiver(s). Compliance will be recorded by measuring the amount of sirolimus returned at each study visit. The person responsible for the clinical trial medication calculates the amount of used medication by subtracting the unused clinical trial medication (in ml) from the total contents of the study medication bottles and comparing it with the documentation of administered medication. The amount of the unused medication and the documentation should be reliable and consistent (with a tolerance of 15%). Details will be recorded in the eCRF.

Inadequately low levels (under the detection level of the laboratory) given the administered dose and not otherwise explicable, e.g. by interindividual rates of metabolization, should raise the suspicion for reduced compliance

Non-compliance (i.e., missed or incorrect dosing) will be documented in the source data and the eCRF. In subjects and / or caregiver(s) of the subject who demonstrate non-compliance between visit intervals, the caregiver(s) of the subject will be counselled by trial staff to address reasons for non-compliance. If after counselling the subject and / or caregiver(s) of the subject continues to exhibit non-compliance over two consecutive trial visits, the subject should be withdrawn from the trial. Dosing interruptions of 7 days or more must be discussed with the sponsor.

5.6 Concomitant Medication and Therapy

A concomitant medication is any treatment received by the subject concomitantly to the IMP. Therapy of TSC manifestations according to SOC will not be affected by the trial, except for manifestations requiring mTOR inhibitor treatment (e.g. SEGA). Treatments in addition to the study medication should be kept to a minimum during the trial. However, if these are considered necessary for the subject’s health and are unlikely to interfere with the IMP, they may be given at the discretion of the investigator, with a stable dose (if possible). Any treatments, which are initiated, continued and/or changed during the trial, must be recorded in the source data and in the eCRF. Medication taken after informed consent is obtained but before the first dose of the study medication will be documented as prior medication. Medication taken after the first dose of the study medication has been administered will be documented as concomitant medication.

Caregivers will be instructed not to administer any medications (over-the-counter or other products) during the treatment period without prior consultation with the investigator. The investigator should instruct caregivers to inform the study site of any new medications taken by the study participant after initiation of treatment with the study medication.
If concomitant drugs (other than study drug) are administered, these must be recorded in the subject file and in the eCRF, stating:

- The type (preferably the generic name / International Nonproprietary Name (INN), or trade name)
- The route of administration
- The regimen, including dosage schedule, daily dose (if not indicated by the type), and form of application
- The indication (heart, infection, neuro, other)
- The duration

Additional treatments, including rescue medication for e.g. prolonged seizures, will be administered according to SOC.

Additional pre-dose blood samples for determination of sirolimus concentrations should be collected one weeks after starting or changing the dose of a CYP3A4 inducer/inhibitor.

5.6.1 Interaction with other medicinal products

The following paragraph is extracted and adopted from the "Rapamune - EPAR - Product Information, ANNEX I, SUMMARY OF PRODUCT CHARACTERISTICS, section 4.5" of the "European Medicines Agency" as of 25.07.2022 [93]:

“Sirolimus is extensively metabolized by the CYP3A4 isozyme in the intestinal wall and liver. Sirolimus is also a substrate for the multidrug efflux pump, P-glycoprotein (P-gp) located in the small intestine. Therefore, absorption and the subsequent elimination of sirolimus may be influenced by substances that affect these proteins. Inhibitors of CYP3A4 (such as ketoconazole, voriconazole, itraconazole, telithromycin, or clarithromycin) decrease the metabolism of sirolimus and increase sirolimus levels. Inducers of CYP3A4 (such as rifampin or rifabutin) increase the metabolism of sirolimus and decrease sirolimus levels. Co-administration of sirolimus with strong inhibitors of CYP3A4 or inducers of CYP3A4 is not recommended.”

Rifampicin (CYP3A4 inducer)

Administration of multiple doses of rifampicin decreased sirolimus whole blood concentrations following a single 10 mg dose of Rapamune® oral solution. Rifampicin increased the clearance of sirolimus by approximately 5.5-fold and decreased area under the curve (AUC) and C_{max} by approximately 82% and 71%, respectively. Co-administration of sirolimus and rifampicin is not recommended.

Ketoconazole (CYP3A4 inhibitor)

Multiple-dose ketoconazole administration significantly affected the rate and extent of absorption and sirolimus exposure from Rapamune® oral solution as reflected by increases in sirolimus C_{max}, t_{max}, and AUC of 4.4-fold, 1.4-fold, and 10.9-fold, respectively. Co-administration of sirolimus and ketoconazole is not recommended.

Voriconazole (CYP3A4 inhibitor)

Co-administration of sirolimus (2 mg single dose) with multiple-dose administration of oral voriconazole (400 mg every 12 hours for 1 day, then 100 mg every 12 hours for 8 days) in healthy subjects has been reported to increase sirolimus C_{max} and AUC by an average of 7-fold and 11-fold respectively. Co-administration of sirolimus and voriconazole is not recommended.

Diltiazem (CYP3A4 inhibitor)

The simultaneous oral administration of 10 mg of Rapamune® oral solution and 120 mg of diltiazem significantly affected the bioavailability of sirolimus. Sirolimus C_{max}, t_{max}, and AUC were increased 1.4-fold, 1.3-fold, and 1.6-fold, respectively. Sirolimus did not affect the pharmacokinetics of either diltiazem or its metabolites desacetyldiltiazem and desmethyldiltiazem. If diltiazem is administered, sirolimus blood levels should be monitored and a dose adjustment may be necessary.

Verapamil (CYP3A4 inhibitor)

Multiple-dose administration of verapamil and sirolimus oral solution significantly affected the rate and extent of absorption of both medicinal products. Whole blood sirolimus C_{max}, t_{max}, and AUC were increased 2.3-fold, 1.1-fold, and 2.2-fold, respectively. Plasma S-(-) verapamil C_{max} and AUC were both increased 1.5-fold, and t_{max} was decreased 24%. Sirolimus levels should be monitored, and appropriate dose reductions of both medicinal products should be considered.

Erythromycin (CYP3A4 inhibitor)

Multiple-dose administration of erythromycin and sirolimus oral solution significantly increased the rate and extent of absorption of both medicinal products. Whole blood sirolimus C_{max}, t_{max}, and AUC were increased 4.4-fold, 1.4-fold, and 4.2-fold, respectively. The C_{max}, t_{max}, and AUC of plasma erythromycin base were increased 1.6-fold, 1.3-fold, and 1.7-fold, respectively. Sirolimus levels should be monitored and appropriate dose reductions of both medicinal products should be considered.

Ciclosporin (CYP3A4 substrate)

The rate and extent of sirolimus absorption was significantly increased by ciclosporin A (CsA). Sirolimus administered concomitantly (5 mg), and at 2 hours (5 mg) and 4 hours (10 mg) after CsA (300 mg), resulted in increased sirolimus AUC by approximately 183%, 141% and 80%, respectively. The effect of CsA was also reflected by increases in sirolimus C_{max} and t_{max}. When given 2 hours before CsA administration, sirolimus C_{max} and AUC were not affected. Single-dose sirolimus did not affect the pharmacokinetics of ciclosporin (microemulsion) in healthy volunteers when administered simultaneously or 4 hours apart. It is recommended that Rapamune® be administered 4 hours after ciclosporin (microemulsion).

Cannabidiol (P-gp inhibitor)

There have been reports of increased blood levels of sirolimus during concomitant use with cannabidiol. Co-administration of cannabidiol with another orally administered mTOR inhibitor in a healthy volunteer study led to an increase in exposure to the mTOR inhibitor of approximately 2.5-fold for both C_{max} and AUC, due to inhibition of intestinal P-gp efflux by cannabidiol. Caution should be used when cannabidiol and Rapamune® are co-administered, closely monitoring for side effects. Monitor sirolimus blood levels and adjust the dose as needed."[93]

Oxcarbazepine and Carbamazepine (CYP3A4 inducer)

Oxcarbazepine is a cytochrome P450 (CYP) 3A4 inducer and sirolimus is primarily metabolized by the cytochrome CYP3A4 and CYP3A5 [81, 82, 90, 94]. Concomitant medication with oxcarbazepine may accelerate sirolimus metabolism by inducing 3A4 activity. At the same weight, the ratios of sirolimus clearance were 1:1.16 from children without oxcarbazepine and children with oxcarbazepine, respectively [90]. These results by Chen *et al.* suggest that the dose of sirolimus should be increased when oxcarbazepine was included in the epilepsy regimen. In addition, carbamazepine is also an inducer of CYP3A4. Theoretically, it may also have potential interaction with sirolimus [90]. We recommend performing trough level measurements of sirolimus

two weeks after starting additional treatments with oxcarbazepine or carbamazepine and adjusting the dose as directed.

Phenobarbital, Phenytoin (CYP3A4 inducer)

Inducers of CYP3A4 may increase the metabolism of sirolimus and decrease sirolimus blood levels. We recommend performing a trough levels of sirolimus one week after starting additional treatments with Phenobarbital or Phenytoin and adjusting the dose as directed.

St. John's Wort (Hypericum perforatum) (CYP3A4 inducer)

May increase the metabolism of sirolimus and decrease sirolimus blood levels. Co-administration of sirolimus and hypericum perforatum is not recommended in this study.

Other Inhibitors of CYP3A4

May decrease the metabolism of sirolimus and increase sirolimus blood levels. Such inhibitors include certain antifungals (e.g. clotrimazole, fluconazole, itraconazole, voriconazole), certain antibiotics (e.g. troleandomycin, telithromycin, clarithromycin), certain protease inhibitors (e.g. ritonavir, indinavir, boceprevir, telaprevir), nicardipine, bromocriptine, cimetidine, danazol and letermovir.

Other possible interactions:

Grapefruit juice

Affects CYP3A4-mediated metabolism and should therefore be avoided.

Vigabatrin

Since vigabatrin is neither metabolised nor bound to proteins and is not an inducer of the hepatic cytochrome P450 enzyme system, drug-drug interactions are unlikely. Very rarely (<1/10,000) hepatitis can occur during treatment with Vigabatrin. Vigabatrin may decrease plasma activity of alanine aminotransferase (ALAT) and to a lesser extent aspartate aminotransferase (ASAT). The extent of ALAT suppression varied between 30-100%. Therefore, quantifying these liver function tests in patients receiving vigabatrin is unreliable.

Propranolol

Propranolol can lead to disturbances in fat metabolism. A decrease in HDL cholesterol and an increase in plasma triglycerides have been observed with mostly normal total cholesterol in plasma. This may lead to an amplification of the effects on fat metabolism by sirolimus. In > 0.1% - < 1% thrombocytopenia was observed. Very rarely (<1/10,000) there may be an increase of the transaminases (ASAT, ALAT).

Gastrointestinal prokinetic agents

Pharmacokinetic interactions may be observed with gastrointestinal prokinetic agents, such as cisapride and metoclopramide.

Breastfeeding:

Mothers of breastfed infants should avoid strong CYP3A4 inhibitors or inducers, as transfer to breast milk may occur. Consumption of rooibos tea and nettle tea has also been associated with enzyme induction in adult patients and should be avoided.

Although sirolimus inhibits human liver microsomal cytochrome P450 CYP2C9, CYP2C19, CYP2D6, and CYP3A4/5 in vitro, the active substance is not expected to inhibit the activity of these isozymes in vivo since the sirolimus concentrations necessary to produce inhibition are

much higher than those observed in patients receiving therapeutic doses of Rapamune®. Inhibitors of P-gp may decrease the efflux of sirolimus from intestinal cells and increase sirolimus levels.

No clinically significant pharmacokinetic interaction was observed between sirolimus and any of the following substances: acyclovir, atorvastatin, digoxin, glibenclamide, methylprednisolone, nifedipine, prednisolone, and trimethoprim/sulfamethoxazole.

5.6.2 Permitted Medication and Therapies

Additional treatments, including rescue medication for e.g. prolonged seizures, will be administered according to standard of care and should not be influenced by the study. Special caution and possible dose adjustments are necessary with the substances mentioned in section 5.6.1).

5.6.3 Prohibited or restricted Medication and Therapies

Patients must not take any other mTOR inhibitors while in the study.

The substances mentioned in section 5.6.1 are not prohibited or restricted, if there is medical necessity and indication. Additional pre-dose blood samples for determination of sirolimus concentrations should be collected one week after starting or changing the dose of any CYP3A4 inducer/inhibitor.

5.6.4 Rescue Medication/Treatment

There is no specific rescue medication for AEs under the treatment with sirolimus. The choice of rescue medications for epileptic seizures (e.g. buccal midazolam, rectal diazepam) should follow the local institution’s practice and guidelines. This may lead to potential interaction via CYP3A4 metabolism.

6. Trial Visits and Investigations/Assessments

The clinical trial period for an individual subject consists of screening examinations, 21-24 months of treatment period (depending on the age at inclusion from 0 to <4 months of age), followed by a one-month follow-up phase. Screening examinations can be taken up to 14 days before study entry including the day of Visit one (V1). Provided all inclusion criteria are met and the informed consent form (ICF) has been signed, Screening and Visit one (V1) may be on the same day. All procedures for Screening must be performed prior to first dose.

Data of SOC visits and additional visits will be entered in the eCRF.

This section describes the trial procedures and tests required to be performed at specified trial visits as outlined in the schedule of assessments. For details on the individual assessments please refer to section 0.

6.1 Definition of standard of care

The standard of care is adapted from “The 2021 *Updated International Tuberous Sclerosis Complex Diagnostic Criteria and Surveillance and Management Recommendations”* [58] including:

<u>Surveillance and Management Recommendations for Diagnosed or Suspected TSC in the first two years of life:</u>

- Genetics: Genetic testing and counseling should be offered to individuals with TSC and suspected TSC and first-degree relatives. Obtain three-generation family history to assess for additional family members at risk of TSC.
- cMRI: Obtain screening MRI of the brain regardless of age to assess for the presence of tubers, subependymal nodules, migrational defects, and SEGA. Follow-up brain MRIs every 1-3 years in asymptomatic children, more frequently in asymptomatic cases with large or growing SEGAs, or associated ventricular enlargement.
- EEG: Obtain screening routine EEG while asleep. If abnormal, consider follow-up with 8 to 24h video-EEG to assess for seizure activity. The frequency of routine EEGs should be determined by clinical need rather than a specific defined interval. Routine video-EEGs during the EPISTOP trial, however, were conducted every 4 weeks (age <6 months), every 6 weeks (age 6-12 months) and every 8 weeks (age >12 months), as is currently practiced by the majority of TSC centers. Prolonged video-EEG for 24h or longer is appropriate when seizure occurrence is unclear or when unexplained sleep or behavioral changes, or other alteration in cognitive or neurological function are present.
- Seizures: Educate parents/caregivers to recognize infantile spasms and focal seizures, even if none have occurred at the time of first diagnosis. Vigabatrin is the first-line therapy for TSC-associated infantile spasms (in accordance with the latest, 02/2021 updated, German AWMF-guideline *“Therapie der Blitz-Nick-Salaam Epilepsie (West-Syndrom), aktualisierte Version 3.0.”[95]*. Other than infantile spasms, antiseizure medications for other seizure types in TSC should generally follow recommendations for other epilepsies.
- Heart: Consider fetal echocardiography to detect individuals with high risk of heart failure after delivery when rhabdomyomas are identified via prenatal ultrasound. Screening echocardiography and ECG at diagnosis if not obtained yet. The frequency of routine echocardiography and ECG should be determined by clinical need rather than a specific defined interval. Follow-up every 6-12 months in asymptomatic patients.
- Skin: Perform a detailed clinical dermatologic inspection/examination at diagnosis and follow-up with annual skin examinations.
- Teeth: Perform a detailed clinical dental inspection/examination at diagnosis and at minimum every 6 months.

- Eye: Perform a complete ophthalmologic evaluation at diagnosis, including dilated fundoscopy, to assess for retinal findings (astrocytic hamartoma and achromic patch) and visual field deficits. Perform annual ophthalmologic evaluation for those with or without visual symptoms at screening.
- Kidney: Obtain ultrasound of kidneys at diagnosis and follow-up every year. Consider MRI of the abdomen to assess for angiomyolipoma and renal cystic disease every 1-3 years throughout the lifetime of the patient. Assess renal function including determination of glomerular filtration rate, proteinuria, and blood pressure at least annually.
- TAND: Screening and annual follow-up screening for TSC-associated neuropsychiatric disorders (TAND). Perform comprehensive assessment for all levels of potential TAND manifestations. If appropriate, refer to suitable professionals to initiate evidence-based interventions based on the TAND profile of above-identified needs.
- Provide parent/caregiver education and training about TAND to ensure families can identify emerging TAND manifestations (e.g. autism spectrum disorder, language disorders, attention-deficit/hyperactivity disorder, anxiety disorders). Provide psychological and social support to families around diagnosis, coming to terms with the diagnosis of TSC and TAND, and ensure strategies are in place to support caregiver well-being.

6.2 Screening Visit

During the screening visit (screening and/or V1, week 0) eligibility criteria will be assessed, and an informed consent form (ICF) has to be signed prior to study related procedures. A careful history of the patient's illness, including TSC diagnosis, prior anti-TSC therapy, seizure history and prior ASM use will be assessed. A physical and neurological examination, including assessment of anthropometric data and vital signs, will be taken by an experienced team of trained pediatric neurologists, nurses and physiotherapists. Initial laboratory examinations (hematology, chemistry, lipids, urinalysis) and a screening EEG will be conducted. Patients will then be randomized. In case randomization to the treatment group occurs, caregivers will be instructed on how to administer the study medication and will further be advised to seek immediate medical attention on signs of pneumonia (respiratory distress), recurrent infection or appearance of oral aphthosis, as well as to avoid interacting CYP3A4 inhibitors and inductors. In both study arms, caregivers will receive a seizure diary and will be instructed on how to recognize and count possible prospective seizures and how to complete a daily seizure diary.

6.3 Titration Visits

In case of any sirolimus dose adjustments, an additional pre-dose PK blood sample should be collected after two weeks, which during the beginning of the trial should coincide with the next regular study visit and after that during an additional appointment.

6.4 Safety Lab Visits

Neonates who receive their first dose of IMP under the age of 4 weeks (corrected gestational age <4 weeks of life): Caregivers will have up to two additional telephone calls by the study personnel up to the end of the 4th week of life. These calls will take place in those weeks (+/-2 days) where no titration visit takes place. Caregivers will be asked about the condition of their child. If it turns out that any sign occurs that requires an on-site visit, the study staff will act accordingly. At the discretion of the investigator, a blood sample could be taken for a safety laboratory. On the day of this onsite-visit, IMP should be given to the child corresponding to the Treatment Visits (see also chapter 5.4). Visits could be on the same day as Titration Visits.
If a blood sample for a safety will be collected (collection of max. 1-2 ml blood), following parameters should be analyzed:

- Blood alcohol concentration (BAC)
- Surrogate parameters for potential propylene glycol (PG) intoxication: electrolytes, osmolality, creatinine, urea, pH, lactate, bicarbonate, anion gap, and the calculated osmol gap

Telephone calls instead of onsite-visits will be performed to reduce stress to the neonates wherever possible.

6.5 Treatment Visits

During the treatment phase of the study there will be visits at week 2 (V2) to months 24 (V12). If applicable, assessment of seizure diaries, the use of anti-seizure medications, adverse events to the study medication and concomitant medications will be done. Furthermore, assessment of anthropometric data, vital signs and physical/neurologic exams will be performed. Laboratory examinations during every study visit include hematology, chemistry, lipids, urinalysis, and pharmacokinetic testing if randomized to the treatment group. In the control group laboratory examinations will be reduced to a minimum at weeks 0 (V1), 6 months (V6), 12 months (V8), 24 months (V12) in order to control for laboratory abnormalities related to TSC. Refusal of participating in laboratory examinations in the control group will not lead to exclusion from the study. Dosing adjustment to the study medication (sirolimus) will be based on pre-dose PK blood sample concentrations (collection of 1-2 ml blood). Sirolimus will be dosed to attain target C_{min} ranges of 5-10 ng/ml. Additional pre-dose PK blood samples should be collected two weeks after any sirolimus dose adjustment. The high frequency of visits during the beginning of the core phase is essential to ensure that titration is being performed effectively and to ensure good compliance.

Additional sirolimus PK sampling: In addition to the time points described above, pre-dose blood samples for determination of sirolimus concentrations should be collected **one** week after the following events:

- Reduction in the dose of a CYP3A4/PgP inducer/inhibitor
- Starting, or changing the dose of, a CYP3A4/PgP inducer/inhibitor

Antiepileptic drug PK sampling: Sirolimus is metabolized by the CYP3A4 pathway in the liver. Many of the concomitantly administered ASMs are also metabolized by CYP3A4, which results in a potential for drug-drug interactions. Therefore, levels of the ASMs listed below (considered to be CYP3A4 substrates and /or inducers) will be measured to investigate the effect of sirolimus on the ASM level.

Risk-Benefit-Evaluation during study visits V6, V8, and V10:
At each study site, two subinvestigators with clinical experience in the treatment of children with TSC will evaluate the individual risk-benefit profile of the participants treated with the IMP at 6, 12, and 18 months of life, considering the individual IMP pharmacovigilance as well as possible novel findings in the research field being considered as relevant for the respective individual. If one of the evaluating pediatricians comes to the decision that the respective participant is unlikely to benefit from continuation of IMP application, the treatment will be terminated. The participant will be continued receiving SOC and will be followed-up until the primary endpoint of the study. The results from each risk-benefit evaluation will be documented in the eCRF and on a separate, signed worksheet at the respective study visit. All decisions will be reported to the coordinating investigator.

6.6 End of Trial Visit

Patients will be followed 1 month (+/-7 days) after end of treatment for continuing or new adverse events as well as serious adverse events and adverse events of special interest. Patients will be instructed to inform about future occurrence of any chronic clinical disease including hematological, oncological or immunological disease during the following 24 months after study end to monitor long-term safety.

6.7 Planned Treatment after End of Trial

There is no planned treatment after end of trial. Parents/caregivers will be instructed to continue therapeutic management according to SOC for all subjects after the end of the trial. The investigator will continue to observe all subjects (also withdrawals) for intolerable (S)AEs until the findings have been clarified or became stable. Currently, there are no data supporting that treatment with sirolimus after the age of 2 years improves cognitive outcomes, so we do not plan to continue administering sirolimus to study participants after the end of the study treatment period. However, if parents/caregivers wish to continue sirolimus treatment, the physician may consider individualized treatment.

6.8 Assessments

Informed consent: The informed consent procedure (date, version, agreement) will be documented.

Inclusion/ exclusion criteria: Once all examinations and procedures are completed and laboratory results are returned, the patient's results should be compared to the inclusion/exclusion criteria list (chapters 4.3 and 4.4). If all criteria are met at the start-of-treatment visit (V1) based on the examination findings and laboratory results from the screening visit, the patient can be randomized and receive the study medication, if randomized to the treatment group. The result of the inclusion/exclusion criteria procedure will be documented.

Diagnosis of TSC: Each patient must have a documented diagnosis of TSC according to the 2021 Updated International Tuberous Sclerosis Complex Diagnostic Criteria [58]. The date and criteria of diagnosis will be recorded in the eCRF.

Table 7: Diagnostic Criteria of TSC

Diagnostic Criteria

Major Criteria	Minor Criteria
Hypomelanotic macules (≥3; at least 5 mm diameter)	"Confetti" skin lesions
Angiofibroma (≥3) or fibrous cephalic plaque	Dental enamel pits (≥3)
Ungual fibromas (≥2)	Intraoral fibromas (≥2)
Shagreen patch	Retinal achromic patch
Multiple retinal hamartomas	Multiple renal cysts
Multiple cortical tubers and/or radial migration lines	Nonrenal hamartomas
Subependymal nodule (≥2)	Sclerotic bone lesions
Subependymal giant cell astrocytoma	
Cardiac rhabdomyoma	
LAM*	
Angiomyolipomas (≥2)*	

Abbreviations:
LAM = Lymphangiomyomatosis
TSC = Tuberous sclerosis complex
Definite TSC: 2 major features or 1 major feature with 2 minor features.
Possible TSC: either 1 major feature or ≥2 minor features.
Genetic diagnosis: A pathogenic variant in *TSC1* or *TSC2* is diagnostic for TSC (most TSC-causing variants are sequence variants that clearly prevent TSC1 or TSC2 protein production. Some variants compatible with protein production [e.g., some missense changes] are well established as disease-causing; other variant types should be considered with caution).
* A combination of the 2 major clinical features LAM and angiomyolipomas without other features does not meet criteria for a definite diagnosis.

Genetic information: Age at genetic diagnosis [months, if applicable], type of investigation [single gene, panel, exome, genome], inheritance [de novo, maternal, paternal, other], Transcript [NM_], Nucleotid change [c.], Amino acid change [p.], other genetic findings or variants [text].

General parameters: Sex, age [months], height [cm], weight without clothing [kg] and occipital-frontal circumference (OFC) [cm] will be documented at screening and measured at each study visit. Investigations are performed according to routine care and are used in the clinical trial analysis.

Relevant medical history /Current conditions: gestational age at birth [weeks + days], OFC at birth [cm], length at birth [cm], weight at birth [g], complications or abnormalities during pregnancy [infection, amniotic fluid complications, bleeding, placental complications, other (text)], mode of delivery [preterm labor, spontaneous, scheduled induction, scheduled caesarean, unplanned caesarean, other (text)], perinatal complications [respiratory distress syndrome, heart problems, temperature control problems, intraventricular hemorrhage, neonatal seizures, anaemia, metabolic problems, other (text)], history of any major illness or concomitant medical conditions [heart failure, cardiac arrhythmia, deterioration of lung function or pulmonary disease, impairment of gastrointestinal function or gastrointestinal disease, liver dysfunction or disease, active skin or mucosa disorders, active or uncontrolled severe infections, known history of HIV seropositivity or

other active viral infections, bleeding diathesis, hyperlipidaemia, major surgery or significant traumatic injury within 4 weeks of study entry, active seizures, renal insufficiency, other (text)]. This information, and whether or not the condition is ongoing at IC will be recorded in the eCRF.

Randomisation: Documentation of the randomisation process including date, time and result.

Prior and concomitant ASM therapy: History of all prior anti-seizure medications and current anti-seizure medications [name of active ingredient, date of onset, date of termination, maximum dose in mg/kg of each drug] will be recorded in the eCRF.

Other concomitant therapies: The caregiver will be queried at each visit regarding the use of any medication other than study medication. All current medications [name of active ingredient, date of onset, date of stop, reason for use, daily dose of each drug] will be recorded in the eCRF. All non-medicinal forms of treatment (e.g. physiotherapy) will be recorded in the eCRF [physiotherapy, other (text)].

Physical examination: The physical examination shall be performed by a qualified examiner and must include an evaluation of the entire body on the undressed patient, including: general appearance [good, normal, impaired], level of alertness [normal, impaired], fontanel (if applicable), skin, eyes, ears, nose, throat, lungs, heart, abdomen, back, genitals, lymph nodes, extremities. Abnormal findings are listed [normal / abnormal (text)] in the patient's eCRF. Significant new findings that occur or worsen after informed consent are additionally entered into the eCRF as Adverse Events (AE).

Neurological examination: The neurological examination will be performed by a qualified examiner and must include an assessment of the entire body on the undressed patient, including (if applicable for age of patient): level of consciousness, cranial nerves, passive tone/posture (assessed by observing the supine resting posture, and measured by the resistance to passive movement of the limbs), active motor tone and strength (symmetric and spontaneous movements evaluated in the axial and appendicular musculature in all extremities), deep tendon reflexes (biceps, patellar and ankle), sensory assessment, and primitive reflexes (stepping reflex, grasp reflexes (palmar and plantar), asymmetrical tonic neck reflex (ATNR), galant reflex) [present/absent]. Primitive reflexes will be assessed until Visit 7. Abnormal findings will be listed on the patient's eCRF [normal / abnormal (text)]. Significant new findings that occur or worsen after informed consent are additionally entered into the eCRF as Adverse Events.

Developmental milestones: Developmental screening is done by asking for and observing the patient’s abilities in relation to age-appropriate developmental norms and rating developmental progress based on information gained from normative populations, as described by R. Michaelis [96] at 3 months, 6 months, 9 months, 12 months, 18 months and 24 months [97].

Vital signs: Vital signs [pulse rate in beats per minute (bmp), respiratory rate per minute, systolic and diastolic blood pressure in mmHg, and body temperature in °C] will be measured in a supine position and will be repeated at each visit and entered in the eCRF starting at the screening visit until the End-of-trial-visit. Vital signs may be recorded at any time, if medically imperative for clarification of clinical signs and symptoms. Pathological and clinically relevant findings will be documented as (S)AEs.

Seizure diary: A paper seizure diary for the recording of epileptic seizures will be given to the caregivers at the time of inclusion into the study and will be collected at each study visit and replaced by a new seizure diary. Seizure events recorded by the caregivers will be transferred to the eCRF by study personnel. Documentation for each new seizure diary: seizures [yes/no], if yes: frequency of seizures [weekly = 1, daily = 2, more than daily = 3], type(s) of seizure(s) [Focal motor onset (e.g. automatism, atonic, clonic, hyperkinetic, myoclonic, tonic), Focal nonmotor onset (e.g. autonomic, behaviour arrest, cognitive, emotional, sensory), Focal to bilateral (tonic-clonic), Generalized onset motor (e.g. tonic-clonic, clonic, tonic, myoclonic, myoclonic-tonic-clonic,

myoclonic-atonic, atonic), Generalized onset non-motor (absence, typical or atypical), Unknown onset motor (tonic-clonic), Unknown onset non-motor (behaviour arrest), Infantile spasms], status epilepticus [n], occurrence and duration of worst episode [no status epilepticus = 0, status lasting half hour to 1h = 2, status lasting 1 h or more = 3], response to treatment in last 3 months [complete cessation of seizures = 1, partial cessation of seizures = 2, no improvement in seizures = 3]. At the time of study inclusion, caregivers will be instructed in the use of the seizure diary and informed about different seizure types, including infantile spasms.

Risk-Benefit-Evaluation:
At each study site, two subinvestigators with clinical experience in the treatment of children with TSC will evaluate the individual risk-benefit profile of the participants treated with the IMP at 6, 12, and 18 months of life, considering the individual IMP pharmacovigilance as well as possible novel findings in the research field being considered as relevant for the respective individual. These evaluations are implemented into the study visits V6, V8, and V10. If one of the evaluating pediatricians comes to the decision that the respective participant is unlikely to benefit from continuation of IMP application, the treatment will be terminated. The participant will be continued receiving SOC and will be followed-up until the primary endpoint of the study. The results from each risk-benefit evaluation will be documented in the eCRF and on a separate, signed worksheet at the respective study visit. All decisions will be reported to the coordinating investigator.

Bayley Scales of Infant and Toddler Development III (BSID-III, cognitive scale): The BSID-III is used to assess current cognitive, language, and motor functioning levels. Within the scope of the study, only cognition is examined. During the examination, tasks arranged by difficulty are presented to the child in a predetermined order. Age-related starting points as well as reversal and exit rules allow for a differentiated and time-effective assessment of the child's developmental level. Results will be entered in the eCRF by study personnel at each site after the external examiner has completed and scored the paper-based examination.

- Raw value (Kog_RW);
- Subtest value (Kog_UTW);
- Scale value (Kog_SK)

Vineland III Adaptive Behaviour Scale (VABS-3): The Vineland Adaptive Behaviour Scales - Third Edition (Vineland-3) is a multidimensional questionnaire procedure for third-party assessment of adaptive behaviour in children, adolescents, and young adults. The procedure is used, among other applications, as part of the diagnosis of intelligence impairment, developmental disorders and autism spectrum disorders. The form is completed by the participants guardian(s) or caregiver(s). The questionnaires can be administered and evaluated in paper form or in digital form via Q-global. Different scale scores (including communication, daily living skills, social skills) as well as a global score (the total adaptive behaviour score) can be determined. The Vineland Scale will be completed at 12 and 24 months of age +/- 2 weeks and results of scale scores and global scores will be entered in the eCRF by study personnel at each site after the external examiner has completed and scored the examination.

Communication:

- raw score listening and understanding (ZuV_RW)
- v-scale value listening and understanding (ZuV_v-W)
- raw value speaking (Spr_RW)
- v-scale value speaking (Spr_v-W)
- raw value reading and writing (LeS_RW)
- v-scale value reading and writing (LeS_v-W)
- communication scale value (KOM_SW)

Daily living skills:

- raw score Caring for oneself (Fss_RW)
- v-scale value Caring for oneself (Fss_v-W)
- household raw score (Haa_RW)
- v-scale value Household (Haa_v-W)
- raw value Living in the community (Gs_RW)
- v-scale value living in the community (Gs_v-W)
- everyday skills scale value (AF_SW)

Social skills:

- raw score Interaction with others (Umg_RW)
- v-scale value Interaction with others (Umg_v-W)
- raw score Play and leisure (Spi_RW)
- v-Scale value Play and Leisure (Spi_v-W)
- raw score Adjustment (Anp_RW)
- v-Scale Value Adjustment (Anp_v-W)
- social skills scale value (SF_SW)

Total value adaptive skills:

- scale value GAV (GAV_SW)

Gross motor skills and Fine motor skills:

- gross motor skills raw score (Gmo_RW)
- v-scale of gross motor skills (Gmo_v-W)
- fine motor skills raw score (Fmo_RW)
- v-scale of fine motor skills (Fmo_v-W)
- scale value MOT_(MOT_SK)

<u>Diagnostic Observation Scale for Autistic Disorders-2, toddler module (ADOS-2):</u> ADOS-2 is a reliable, valid and clinically very descriptive procedure for the clarification and classification of qualitative abnormalities of social interaction and reciprocal communication in autism spectrum disorders. The structured rating scale belongs to the international standard of diagnostics of autistic spectrum disorders. The toddler's module is selected in order to be able to examine facts and symptoms relevant for the diagnosis of autism on the basis of specifically staged playful elements, activities and conversations in the study participant at 12 and 24 months of age +/- 2 weeks. Results of scale scores and global scores will be entered in the eCRF by study personnel at each site after the external examiner has completed and scored the examination.

Each time a test is performed, the module used for the assessment must be recorded beforehand. This is not determined by age, but primarily by language skills (e.g., fluent speech, individual words, ...).

- Item-encodings: A1-9; B1-18; C1-3; D1-5; E1-4
- Raw score Social Affect (ADOS_SA_RW) following conversion of item encodings into algorithm values.
- Raw score Restrictive and Repetitive Behaviors (ADOS_RRV_RW) following conversion of item encodings into algorithm values
- Sum SA and RRV (ADOS_SA_RVV_SW)
- Age-specific comparison score (ADOS_Vergl)
- Symptom level (ADOS_symp)
- Diagnosis (ADOS_Diag)

Modified Checklist for Autism in Toddlers revised (M-CHAT-R/F) at 24 months of age: The Modified Checklist for Autism in Toddlers, Revised with Follow-Up (M-CHAT-R/F [98]) is a two-step screening instrument consisting of a parent/caregivers questionnaire to assess the risk for autism spectrum disorder (ASD). As a result, the M-CHAT returns a value depending on which a suspected autism spectrum disorder should be further clarified.

- abnormal values (Mchat), metric 0-20
- Low risk: total score 0-2; Medium risk: total score 3-7; High risk: total score 8-20; (Mchat_risk)

Screening for TSC-associated neuropsychiatric disorders (TAND): A TAND questionnaire is completed at the participating sites by qualified study personal together with the caregivers. Following results will be entered with date into the eCRF.

Chapter 03: Behaviors (TAND_03).

- Fears (yes/no)
- Depressed moods (yes/no)
- Severe shyness (yes/no)
- Mood swings (yes/no)
- Aggression (yes/no)
- Outbursts of anger (yes/no)
- Self-injurious behavior (e.g., hitting, biting, or scratching oneself) (yes/no)
- Failure or delayed onset of communicative speech (yes/no)
- Constant repetition of words or phrases (yes/no)
- Lack of eye contact (yes/no)
- Difficulty socializing with peers (yes/no)
- Repetitive behaviors (e.g., doing the same thing over and over) (yes/no)
- No or little flexibility in everyday situations or when deviating from routine (yes/no)
- Overactivity or hyperactivity (e.g., being constantly on the go) (yes/no)
- Difficulty with attention and/or concentration (yes/no)
- Restlessness or agitation (e.g., fidgeting, wiggling) (yes/no)
- Impulsivity (e.g., interrupting others, not being able to wait one's turn) (yes/no)
- Problems with eating behavior (e.g., eating too much or too little, unusual preferences) (yes/no)
- Sleep disturbances (e.g., unusual sleeping and/or waking times) (yes/no)

Chapter 09: Burden of problems caregiver (TAND_09) ordinal scale 0-10
Chapter 12: Burden of problems investigator (TAND_12) ordinal scale 0-10

EEG: All EEG recordings will be performed by the participating sites. All recordings should be entered with date and written summary as described in the original report into the eCRF [date]. Additionally, a copy on compact disc (CD) with the patient number, study name and date must be sent to the Coordinating Investigator. A blinded centralised EEG assessment using BESA (Brain Electrical Source Analysis) Research EEG-Rater-Software will be performed at the coordinating site.

Assessments:

Overall rating [normal, conspicuous, pathological].
Slow background activity [none, mild, moderate, severe].
Epileptiform spikes [no, focal, generalized].
Number of epileptiform spike "clusters" [n], proportion of epileptiform activity [percent] per cluster.
Hypsarrhythmia [yes/no]

cMRI: Is performed according to standard of care at time of diagnosis and in 1-3-year intervals. All brain MRIs should be entered as unscheduled visits with date and written summary as described in the original report into the eCRF [date, text]. Additionally, a copy on compact disc (CD) with the patient number, study name and date must be sent to the Coordinating Investigator.

Assessments:

Overall rating [normal, conspicuous, pathological].
Cortical tubers (n), size, if applicable.
subependymal nodules (n), size, if applicable.
subependymal giant cell astrocytoma (n), size, if applicable.
Other findings [text]
Further parameters can be added at a later timepoint for future research questions which are not known at the time of consent; exploratory approach of the data for the evaluation of the examination results.

Echocardiogram: Is performed according to SOC. All examinations should be entered as unscheduled Visits with date and written summary as described in the original report into the eCRF [date, text]. Additionally, a copy on compact disc (CD) with the patient number, study name and date must be sent to the Coordinating Investigator.

Assessments:

Overall rating [normal, conspicuous, pathological].
Cardiac rhabdomyomas (n), size, if applicable.
Other anatomical abnormalities [yes, no], if yes: type [text]
heart failure [yes, no], if yes [text]
Other findings [text]
Further parameters can be added at a later timepoint for future research questions which are not known at the time of consent; exploratory approach of the data for the evaluation of the examination results.

Electrocardiogram (ECG): Is performed according to SOC. All examinations should be entered as unscheduled Visits with date and written summary as described in the original report into the eCRF [date, text].

Assessments:
Overall rating [normal, conspicuous, pathological].
Functional disturbance [yes, no], if yes [text]
arrhythmia [yes, no]

Other findings [text]
Further parameters can be added at a later timepoint for future research questions which are not known at the time of consent; exploratory approach of the data for the evaluation of the examination results.

Abdominal sonography: Is performed according to SOC. All examinations should be entered as unscheduled Visits with date and written summary as described in the original report into the eCRF [date, text].

Assessments:
Overall rating [normal, conspicuous, pathological].
angiomyolipomas (n), size, if applicable.
kidney size [mm],
renal pelvis dilation [yes, no],
echogenicity [normal, abnormal]
Further parameters can be added at a later timepoint.

Laboratory analyses:

Clinical chemistry [albumin, alkaline phosphatase, ALT, AST, calcium, chloride, creatinine, direct bilirubin, total bilirubin, LDH (lactate dehydrogenase), magnesium, phosphate, potassium, sodium, uric acid, creatinine clearance (calculated)].

Haematology [leukocytes, neutrophils, eosinophils, basophiles, lymphocytes, monocytes, erythrocytes, thrombocytes, haematocrit, haemoglobin].

Lipid panel [triglycerides, total cholesterol (fasting), LDL, HDL]. The patient must be in a fasting state (2h for infants who are breastfed or receive formula milk feeding and 4h for infants and children who receive solid food) at the time of blood sampling for the lipid profile evaluation.

Urinalysis: Dipstick measurements in spot-urine for protein, ketones, bilirubin, nitrites, urobilinogen, glucose, pH and blood will be performed. Any significant findings on dipstick will be followed up with a microscopic evaluation, where WBC (White Blood Cells) and RBC (Red Blood Cells) sediments will also be measured. In the case a urine sample cannot be obtained, this test can be omitted.

After collection the samples will immediately be delivered to the laboratory for respective determinations. All parameters will be documented on appropriate eCRF-pages.

Further laboratory parameters may be determined at any time during the clinical trial at discretion of the responsible investigator. Pathological and clinically relevant findings will be documented as (S)AEs.

Safety laboratory analyses:

- Blood alcohol concentration (BAC)
- Surrogate parameters for potential propylene glycol (PG) intoxication: electrolytes, osmolality, creatinine, urea, pH, lactate, bicarbonate, anion gap, and the calculated osmol gap

Accompanying research:

- RNA samples will be collected for accompanying research at screening and 24 months (V12) (PAXgene® Blood RNA System, 2,7ml).
- DNA samples will be collected for accompanying research at 24 months (V12) of age.

- Vaccination titres: the titers of vaccines applied during the first year (measles, mumps, rubella, tetanus, pertussis, varicella) will be assessed in both groups.

Special tubes, packaging and labels will be provided by the sponsor or his delegate(s). After sample collection, probes will be labelled and send immediately to the Institute of human genetics Leipzig for RNA and DNA, according to the investigator's manual provided by the sponsor or his delegate(s).

Pharmacokinetics

During the titration period, pre-dose PK blood samples will be collected immediately prior to dosing on the study day. Any sample collected outside of these parameters is not considered a true trough and therefore should not be used as a basis for adjusting the patient's dose. It is expected that all patients will achieve the desired target pre-dose trough level (C_{min}) during the titration period (V1-V4). A local lab (at the treating centre) will measure the sirolimus concentrations. HPLC-MS/MS is the most appropriate method, other techniques (e.g. immonoassays) tend to under-/overestimating of drug concentrations. After obtaining current trough levels, the site will adjust the future dose as described in section 5.5.3 (to maintain, increase or decrease the dose). In order to minimize total blood samples, the amount of blood required for sirolimus PK samples will be 0.5 mL.

Therapy

The current dose and all dose adjustments (to maintain, increase or decrease the dose) of study medication [mg] will be entered into the eCRF at each study visit for the treatment group.

7. Discontinuation and early Termination

Any legal guardian(s) of the subject can withdraw from the treatment or the clinical trial verbally or in writing at any time without personal disadvantages and without having to provide a reason. However, the investigator should make a reasonable effort to ascertain possible reasons, while fully respecting the subject's rights. Specifically, the legal guardian(s) of the subject must not be coerced or unduly influenced to continue to participate. Any given reason should be documented in the subject's file and in the eCRF.

The investigator can also discontinue the study medication after considering the risk-to-benefit ratio, if he / she no longer considers the treatment justifiable. The date of and the primary reason for the withdrawal (one primary reason must be determined), as well as the observations available at the time of withdrawal are to be documented in the eCRF.

For details concerning sample and statistical considerations see 9.1.

7.1 Temporary Discontinuation from Treatment with the IMP

Temporary treatment (IMP) discontinuation at the discretion of the investigator is defined as one or more doses not administered to the subject.

Temporary treatment discontinuation may be considered by the investigator because of the following:

- Sirolimus may affect the response to vaccination. Sirolimus should be paused 2 weeks before until 2 weeks after receiving a live vaccine. The SmPC of Sirolimus recommends avoiding live vaccines during treatment with Sirolimus. Individual risks/ benefits should be discussed with infectious disease specialists.
- Surgical interventions: Treatment with sirolimus may affect wound healing. We recommend pausing treatment 14 days before until 14 days after elective surgical interventions. For non-elective interventions, therapy should also be paused until 14 days after the procedure.
- For a single grade 3 adverse event or for less serious adverse events, e.g. uncomplicated (viral) infections, sirolimus can be temporarily discontinued at the discretion of the investigator (for details see chapter 5.5.3)
- Grade 4 adverse event (for details see chapter 5.5.3)

7.2 Permanent Discontinuation from Treatment with the IMP

Permanent treatment discontinuation with the IMP is any treatment discontinuation associated with the investigator's or the legal guardian(s) of the subject's definitive decision not to re-expose the subject to the IMP. The following criteria will lead to a permanent treatment discontinuation:

- Withdrawal of consent to trial participation
- Loss to follow-up
- Death
- Legal guardian(s)'s request, i.e. withdrawal of consent for treatment → documentation in source data
- Use of illicit drugs, prohibited concomitant medications (e.g. other mTOR inhibitors), or other substances that, in the opinion of the investigator, are likely to increase toxicity or otherwise confound the results while taking the IMP.
- Recurring grade 4 adverse events (AE) or serious and unexpected adverse events (SAE) related to the IMP require permanent discontinuation of sirolimus.

- Lack of compliance, e.g. caregivers and participants fail to attend interim visits or to perform self-medication according to schedule.
- Any additional diagnosis due to which, in the investigator's opinion, participation in the treatment group of the trial may pose a risk for the study participant or that may interfere with protocol adherence.
- Negative Risk-Benefit Evaluation
- Subject did not meet in-/exclusion criteria (coming to light after inclusion), if it poses a risk for the study participant to continue the IMP in the opinion of the investigator.

The reason for the permanent discontinuation of treatment with IMP has to be documented in the source data.

Even if the trial treatment with the IMP is discontinued, the subjects may remain in the trial and, given the legal guardian(s) oral agreement (documentation in source data), will be followed-up as outlined in the schedule of assessments (further study visits according to the control group) and section 6.

7.3 Discontinuation of Trial

The following incidents will lead to discontinuation of a patient from the trial:

7.3.1 Withdrawal of Consent

Legal guardian(s) of the subjects may withdraw his/her/their consent to trial participation at any time without the need to justify the decision. If legal guardian(s) of the subject wants to withdraw his/her/their consent, the investigator should explain the difference between treatment discontinuation and withdrawal from trial participation, including the options for continued follow-up after discontinuation of the study medication (see section 7.2).

If the legal guardian(s)'s withdraw their consent for further study visits, they should be asked if they give their consent to take part at least in V12 (visit with 24 months of age) with respect to the primary endpoint measurement. The consent has to be as an additional agreement in written form on the last given written Informed Consent Form. 7.3.1

The withdrawal of consent has to be documented in the source data.

Usually, the legal guardian(s) of the subject's withdrawal of consent only relates to future information. Therefore, the sponsor may retain and continue to use any data collected before the withdrawal of consent.

7.3.2 Lost to Follow-up

Subjects will be considered lost to follow-up if they fail to return for visits and their caregiver(s) cannot be contacted by the trial site. Site personnel is expected to make diligent attempts to contact caregiver(s) of the subjects who did not appear for a scheduled visit or were otherwise not accessible by the site. These contact attempts should be documented in the subject's medical record.

7.3.3 Death

7.4 Criteria for Discontinuation of Dose Escalation

Not applicable.

7.5 Temporary Halt of the Clinical Trial

A temporary halt of a clinical trial is defined as an unforeseen interruption not provided in the protocol but with the intention to resume it. A temporary halt can be part of an urgent safety measure. If the reason for the temporary halt has the potential to affect the benefit-risk balance, the re-start is only possible through a substantial modification.

7.6 Early Termination of the Clinical Trial

Early termination is defined as the premature end of a clinical trial before the conditions specified in the protocol are met.

The following reasons or events may result in an early termination:

- The information on the product leads to doubt as to the benefit-risk ratio and safety concerns arise, the sponsor reserves the right to interrupt or terminate the trial, also if new scientific information make continuation unnecessary or impossible.
- Subject enrolment is insufficient and that it cannot be expedited by appropriate measures so that it is no longer possible to evaluate the primary endpoint.

DSMB recommends termination of the clinical trial or of treatment arms (see also section 12.5.1). An early end of the trial due to early inclusion of the total number of subjects is not considered an early termination. In the event of premature discontinuation of the trial for any reason whatsoever, the regulatory authorities should be informed according to applicable regulatory requirements. In case of an early termination of the trial, the date of the early termination will be the date of the end of trial. All involved investigators have to be informed immediately about a cessation / suspension of the clinical trial. The decision is binding to all clinical trial centres and investigators.

When the clinical trial is closed, all clinical trial materials (e.g. unused questionnaires) must be returned to the sponsor.

7.7 Premature Close-out of a Site

Premature closure of a single trial site by the sponsor may be considered for the following reasons:

- The investigator failed to recruit any subjects even though he/she had received all IMPs, means and information necessary to perform the clinical trial and had reasonable time to do so.
- Non-compliance of the investigator, sub-investigator or delegated staff with ICH-GCP, any provision of the clinical trial protocol or breach of the applicable laws and regulations.
- Structural problems and / or shortage of staff at trial site which affect the safe conduct of the study.
- Premature termination of a single centre is also possible if the sponsor notices that the conduction of the clinical trial is not compliant with ICH-GCP and / or the protocol and / or the quality of the data is insufficient.

The investigator may terminate participation in the trial himself/herself if at his/her own discretion the site or the investigator becomes unable to perform or complete the clinical trial according to the agreement in place. If this is possible at the discretion of the principal investigator, study participants already enrolled will be followed up until the end of the trial, or transferred to a nearby study site.

In the event of premature close-out for any reason whatsoever, the regulatory authorities should be informed according to applicable regulatory requirements. When trial site is closed, all clinical trial materials must be returned to the sponsor.

8. Adverse Events

8.1 Definitions

8.1.1 Adverse event

According to GCP, an adverse event (AE) is defined as follows: Any unexpected medical occurrence in a subject to whom a medicinal product is administered and which does not necessarily have a causal relationship with this treatment. An AE can therefore be any unfavorable and unintended sign (including an abnormal laboratory finding), symptom, or disease temporally associated with the use of an investigational medicinal product (IMP), whether or not related to the IMP.

An AE may be:

- New symptoms/medical conditions
- New diagnosis
- Changes of laboratory parameters which are considered as clinically significant
- Occurrence of new pathological and clinically relevant findings
- Aggregation of pre-existing symptoms in physical and neurological examinations, vital signs, ECGs, clinical chemistry, haematology etc.

The criteria that should be considered when determining whether an abnormal test finding should be reported as adverse event are as follows:

1. Test result is associated with accompanying symptoms, and/or
2. Test result requires diagnostic testing or medical/surgical intervention, and/or
3. Test result leads to a change in clinical trial dosing outside the protocol-stipulated dose adjustments, or discontinuation from the clinical trial, significant additional concomitant drug treatment, or other therapy, and/or
4. Test result is considered clinically relevant at the discretion of the investigator or sponsor
5. Intercurrent diseases and accidents
6. Worsening of medical conditions/ diseases existing before clinical trial start
7. Recurrence of disease
8. Increase of frequency or intensity of episodic diseases.

A pre-existing disease or symptom will not be considered an adverse event unless there will be an unexpected change in its intensity, frequency or quality. This change will be documented by an investigator (see also above).

Surgical procedures themselves are not AEs; they are therapeutic measures for conditions that require surgery. The condition for which the surgery is required may be an AE. Planned surgical measures permitted by the clinical trial protocol and the condition(s) leading to these measures are not AEs, if the condition leading to the measure was present prior to inclusion into the clinical trial.

In the latter case the condition should be reported as medical history.

AEs are classified as "non-serious" or "serious".

8.1.2 Serious Adverse Event

Serious adverse event (SAE) is any unexpected medical occurrence that at any dose:

1. Results in death
2. Is life-threatening (the term life-threatening refers to an event in which the subject was at risk of death at the time of event and not to an event which hypothetically might have caused death if it was more severe)
3. Requires inpatient hospitalization or prolongation of existing hospitalization
4. Results in persistent or significant disability/ incapacity*
5. Is otherwise medically relevant

* Persistent or significant disability or incapacity means that there is a substantial disruption of a person's ability to carry out normal life functions. The irreversible injury of an organ function (e.g. paresis, diabetes, cardiac arrhythmia) fulfils this criterion.

Medical and scientific judgement should be exercised in deciding whether expedited reporting to the sponsor is appropriate in other situations - such as important medical events that may not be immediately life threatening or result in death or hospitalization but may jeopardize the subject or may require intervention to prevent one of the other outcomes listed above. These should also usually be considered serious (examples of such events are intensive treatment in an emergency room or at home for allergic bronchospasm; blood dyscrasias or seizures that do not result in hospitalization).

However, since the following events do not change the ongoing benefit-risk assessment of the current clinical trial, they do not need to be reported as SAEs:

- Hospitalization aiming exclusively at diagnostic measures, SOC treatment, or due to technical, practical or social reasons, i.e. hospitalization without underlying adverse event

8.1.3 Serious Adverse Reaction

SAEs that potentially may be attributed to the investigational medicinal product (IMP) are to be classified as Serious Adverse Reactions (SARs).

8.1.4 Adverse Events of Special Interest (AESI)

The following events are defined as adverse events of special interest (AESI) in the current clinical trial and have to be reported by investigator in accordance with the requirement for reporting of SAE, even if none of the seriousness criteria apply:

- **Clinically significant elevation of AST and/or ALT** (grade 3 or grade 4) in combination with clinically significant elevation of total bilirubin will be reported as AESI (Hy´s Law).
- Clinical apparent infection leading to unplanned medical consultation without hospitalization
- Unexplained rashes (e.g. petechial rash) leading to unplanned medical consultation without hospitalization

8.2 Expectedness

The expectedness of an adverse reaction is set out in the Reference Safety Information (RSI). Only those SAR which are listed in the RSI may be considered ‘expected’. In determining whether an adverse event is unexpected, consideration shall be given to whether the event adds significant information on the specificity, increase of occurrence, or severity of a known, already documented serious adverse reaction. Specific examples would be (a) acute renal failure as an expected

adverse reaction with a subsequent new occurrence of interstitial nephritis and (b) hepatitis with a first occurrence of fulminant hepatitis.

In this trial Additional Information on Sirolimus including Reference Safety Information will be used as RSI. Each serious adverse reaction (SAR) is to be classified as unexpected.

8.3 Suspected Unexpected Serious Adverse Reaction (SUSAR)

SAEs that are both ‘suspected’, i.e., potentially related to the investigational medicinal products (IMPs) and ‘unexpected’ (for definition see above) are to be classified as Suspected Unexpected Serious Adverse Reactions (SUSARs).

In case, either the investigator who initially reported the SAE or the Sponsor’s designated person(s) (second assessor(s)), classifies the SAE as potentially related to the IMPs and the SAE is ‘unexpected’ it will be categorized as a SUSAR.

All SUSARs are subject to an expedited reporting (see section 8.8).

8.4 Characteristics of Adverse Events

8.4.1 Grading of AEs

The grading of AEs in this clinical trial will be carried out based on the 5-grade scale defined in the CTCAE V 5.0:

Grade 1:	Mild; asymptomatic or mild symptoms; clinical or diagnostic observations only; intervention not indicated.
Grade 2:	Moderate; minimal, local or non-invasive intervention indicated; limiting age-appropriate instrumental ADL*.
Grade 3:	Severe or medically significant but not immediately life-threatening; hospitalization or prolongation of hospitalization indicated; disabling; limiting self-care ADL**.
Grade 4:	Life threatening consequences; urgent intervention indicated.
Grade 5:	Death related to AE.

Activities of Daily Living (ADL)

*Instrumental ADL refer to preparing meals, shopping for groceries or clothes, using the telephone, managing money, etc.

**Self-care ADL refer to bathing, dressing and undressing, feeding self, using the toilet, taking medications, and not bedridden.

The grading of all AEs listed in the CTCAE v 5.0 will be based on the information contained therein. The grading of all other AEs, i.e., those which are not listed in the CTCAE v 5.0 will be performed by a responsible investigator, based on definitions given above.

If an AE shows an undulating course of intensity, it must be documented only once with predominant or medically most appropriate intensity.

<u>Clarification of the difference in meaning between "serious" and "severe</u>": The terms “serious” and “severe” are not synonymous. The term “severe” should be used to describe the intensity (severity) of a specific event; the event itself, however, may be of relatively minor significance (such as severe headache). This is not the same as “serious”, which is based on the existence of at least one of the above-mentioned seriousness criteria.

8.4.2 Causal Relationship

The investigator will evaluate each AE that occurred after administration of the IMP regarding the **relationship** with the IMP:

Related	There is a reasonable possibility of a causal relationship between the event and the IMP, i.e. the possibility for a causal relationship between the IMP and the AE cannot be excluded.
Not related	There is no reasonable possibility of a causal relationship between the AE and the respective IMP, i.e. there is a clear alternative explanation.

8.4.3 Outcome of AEs

All subjects who have reportable AEs, whether considered associated with the use of the clinical trial medication or not, must be monitored to determine the **outcome**. The clinical course of the AE will be followed up until resolution or normalization of changed laboratory parameters or until it has changed to a stable condition. This also holds for ongoing AEs/SAEs of withdrawn subjects.

The **outcome** of an AE at the time of the last observation will be classified as:

Recovered / resolved:	All signs and symptoms of an AE disappeared without any sequelae at the time of the last interrogation.
Recovering / resolving:	The intensity of signs and symptoms has been diminishing and / or their clinical pattern has been changing up to the time of the last interrogation in a way typical for its resolution.
Not recovered / not resolved:	Signs and symptoms of an AE are mostly unchanged or worsened at the time of the last interrogation.
Recovered / resolved with sequelae:	Actual signs and symptoms of an AE disappeared but there are sequelae related to the AE.
Fatal:	Resulting in death. If there is more than one adverse event only the adverse event leading to death (related) will be characterized as ‘fatal‘.
Unknown	The outcome is unknown or implausible and the information cannot be supplemented or verified.

8.4.4 Action taken with the IMP

The **action taken with the IMP** will be assigned to one of the following categories:

Dose not changed:	No change in the dose of the IMP.
Dose reduced:	Reduction in the dose of the IMP.
Drug withdrawn:	Discontinuation of the IMP.
Unknown:	The information is unknown or implausible and it cannot be supplemented or verified.
Not applicable:	The question is implausible (e.g. the subject is dead).

8.4.5 Countermeasures

The term "**countermeasures**" refers to the specific actions taken to treat or alleviate adverse events or to avoid their sequelae. The following categories will be used to classify the countermeasures taken for adverse events:

None:	No action taken.
Drug treatment:	Newly-prescribed medication or change in dose of a medication.
Others:	Other countermeasures, e.g. an operative procedure.

8.5 Special Situations Reporting

The following events are also to be immediately reported to the sponsor even if they do not result in an adverse event (for reporting obligations see section 8.7.1):

Medication error: This is an unintended failure in the IMP treatment process that leads to, or has the potential to lead to harm to the subject. This includes mistakes in the prescribing, dispensing, storing, preparation and administration of the IMP. The following deviations from the treatment are not regarded as medication errors:

- The medication was refused, spat out, or swallowed incompletely by the study participant.

Misuse: This refers to situations where the investigational medicinal product is intentionally and inappropriately used not in accordance with the terms of the trial protocol.

Overdose: This refers to the administration of a quantity of the investigational medicinal product given as repeated overdosing more than the double of intended dosage per administration. The following deviations are not regarded as overdose:

- Single overdosing up to double of intended dosage

Use outside of what is foreseen in the protocol: This relates to situations where the investigational medicinal product is used for a purpose being not in accordance with the indication defined in the trial protocol.

Abuse: This corresponds to the persistent or sporadic, intentional excessive use of the investigational medicinal product, which is accompanied by harmful physical or psychological effects.

Environmental exposure

This refers to the incidental exposure of a person other than the study participant to the investigational medicinal product. This may include exposure during pregnancy and lactation if the person is exposed incidentally to the product.

Occupational exposure

This refers to the exposure to a medicinal product, as a result of one's professional or non-professional occupation. It does not include the exposure to one of the ingredients during the manufacturing process before the release as finished product.

The reportable events listed above may be considered potential serious breaches after consultation with the second assessor. If applicable, they will be handled as described in section 13.5.

8.6 Period of Observation and Documentation

8.6.1 Adverse Events (AEs)

AEs will be ascertained by the investigators using non-leading questions, noted as spontaneously reported by the caregiver(s) of the subjects to the medical staff at any time during the trial or observed at any of the clinical trial visit.

The observational period begins with the signature of the informed consent form (ICF) and ends with the end of trial visit (V13). Same applies for SAEs/AESIs. AEs will be interrogated for at each contact between the responsible investigator and the caregiver(s) of the clinical trial subject. Thereafter, the investigator does not need to actively monitor subjects for adverse events. However, if the investigator becomes aware of a serious adverse event with a suspected causal relationship to the IMP (SAR) that occurred after the end of the observational period he or she shall report the SAE without undue delay to the Sponsor, as long as the clinical trial is still ongoing. This kind of SARs is also to be documented in the eCRF.

AEs will be documented in the subject file and in the eCRF. AEs will be reported with patient number, start and end date, description, grading, seriousness, relationship, action taken and outcome. These are the parameters that can be found on the standard AE-eCRF page. If applicable, all medical diagnoses or symptoms occurring prior to the beginning of the period of observation and documentation will be recorded in the eCRF as medical history.

The following general rules apply to the documentation of the AEs and SAEs: The start date of a SAE must not be earlier than that of the corresponding AE. The end date of a SAE is typically the same as that of the AE. The end date of the SAE must not be later than the end date of the corresponding AE. AEs and SAEs that are ongoing at the time of death are considered not resolved or resolving.

All SAEs/AESIs and their relevance for the benefit-risk assessment of the clinical trial will be evaluated continuously during the clinical trial and for the final report. All SAEs/AESIs will be documented in the eCRF and in the ‘SAE/AESI Report Form’ (see section 8.7.1).

8.6.2 Special Situations

Special situations (cases of medication error, misuse, overdose, use outside what is foreseen in the protocol, abuse, definitions see also chapter 8.5) shall be documented in the “Special Situation Reporting Form”. All other events may only occur in a close temporal connection with the administration of the IMP. However, if a reportable event comes to the attention of the investigator with a delay, it still has to be appropriately documented and reported to the Sponsor (see section 8.7.1).

8.7 Investigator’s Pharmacovigilance related reporting Obligations

8.7.1 Reporting of Serious Adverse Events, Adverse Events of Special Interest and Special Situations

All SAEs and AESIs must be reported by the investigator to the PV department of KKS Heidelberg **immediately, but not later than 24 hours after the SAE/AESI becomes known** using the ‘SAE/AESI Report Form’. The initial report must be as complete as possible including details of the (serious) adverse event and an assessment of the causal relationship between the event and the IMPs.

All SAEs, AESIs are reportable to Pfizer by the PV department of KKS Heidelberg.

Exposure to the Pfizer Product such as drug overdose, medication error, occupational/environmental exposure to the Pfizer Product are reportable to Pfizer. Medication

errors and overdoses are reportable only if associated with an SAE. Occupational/ environmental exposure is reported independently from any associated AE/SAE.

All other reportable events (see section 8.6.2) must also be reported **immediately, but not later than 24 hours after they come to the attention of the investigator** by the use of the "Special Situation Reporting Form". All available information must be included.

The reporting will be performed by faxing or e-mailing (in case of technical issues) of a completed 'SAE/AESI Report Form' / "Special Situation Reporting Form" to the PV department of KKS Heidelberg,

fax number: +49 (0)6221/56 33725
or e-Mail: pharmakovigilanz.KKS@med.uni-heidelberg.de

8.7.2 Reporting of Pregnancies

Not applicable.

8.8 Sponsor's Pharmacovigilance related Obligations

8.8.1 Sponsor's Assessment (Second Assessment)

All SAEs/AESIs will be subject to a **second assessment** by the sponsor's designated persons, who will be independent from the reporting investigator. Moreover, all other reportable events will be brought to the attention of the second assessor(s).

Despite the nature of the current trial (not blinded safety parameters are important trial endpoints), the assessor could be a study team member due to feasibility reasons and the fact that the primary endpoint is an efficacy endpoint.

The second assessor will fill out a "Second Assessment Sheet" for each SAE/AESI and send it back per fax or E-Mail to the PV department of KKS Heidelberg within 48 hours,

fax-number: +49 (0)6221/56 33725

or e-Mail pharmakovigilanz.KKS@med.uni-heidelberg.de

The 'Second Assessment Form' will contain the following information:

- assessment of relationship between SAE/AESI and IMP(s)
- assessment of relationship between SAE/AESI and the underlying disease (trial indication)
- assessment of expectedness of SAE/AESI (derived from RSI, i.e. SmPC)
- statement if the benefit/ risk assessment for the clinical trial did change as a result of SAE/AESI.

8.8.2 Expedited Reporting of SUSARs

SUSARs are to be reported to the competent authorities of all member states concerned via electronic transmission to the EudraVigilance portal within defined timelines, i.e. they are subject to an expedited reporting.

The expedited reporting to the competent authorities of all member states concerned will be carried out by the PV department of KKS Heidelberg.

The investigators will not be informed about each individual SUSAR. Instead, they will be provided with the executive summary of ASR (see section 8.8.3)

8.8.3 Reporting of Adverse Reactions of AxMP

All medicinal products defined as AxMPs in the current trial have market authorization in Germany, Austria and Switzerland. Therefore, all adverse reaction of AxMP will be reported in accordance with the applicable Medical Associations' professional codes of conduct.

8.8.4 Annual Safety Report

Once a year an annual safety report (ASR) regarding this single trial will be prepared by the PV department of KKS Heidelberg. The main reason for preparation of a trial-specific ASR is that the Sponsor does not have a knowledge of all ongoing clinical investigations involving the IMP(s).

The international birth date (IBD) is defined as the date of first authorization to conduct the first clinical trial with the IMP in any country worldwide. In accordance with the list 'European Union Reference Dates' (EURD), the IBD applicable for this trial is 13.03.2001 (IBD for *name of IMP*).

The format of ASR will be based on the guidance on content provided in the ICH guideline E2F.

As already mentioned above, the nature of the current trial precludes insight of the Sponsor or his/her representatives in the safety data. This is especially important for data presented in a cumulative form. Therefore, ASR will be prepared by an independent second assessor(s) in cooperation with the PV department of KKS Heidelberg. The Sponsor will only be informed if any measures are required. The Sponsor will be informed by provision of the executive summary of the ASR.

In order to keep all investigators informed about the main trial-related safety findings and conclusions, they will be provided with the executive summary of ASR and with the tables/listings attached to the ASR.

Concurrently with the ASR two versions of Reference Safety Information (RSI) will be provided: i) the RSI applicable at the beginning of the reporting period and, if applicable, ii) the version submitted for approval / approved within the current reporting period.

The ASR will be submitted via EU Portal (CTIS).

9. Statistical Procedures

9.1 Definition of Clinical Trial Population to be Analyzed

The **safety analysis set** (SAF) comprises all randomized patients and is identical with the **full analysis set** (FAS). The **per-protocol set** (PPS) comprises all patients of the FAS who followed treatment for at least 9 months and completed neuropsychologic testing (BSID-III) at 2 years. All patients of the PPS need to fulfil the inclusion criteria 1 and exclusion criteria 1 and 2. For efficacy analyses the patients will be analysed as allocated, for safety analyses as treated.
For the analyses “as treated”, participants randomized to the Sirolimus arm are regarded as treated according to standard of care only, if

- They are lost to follow-up before 6 months after randomization or
- They never started treatment

Participants randomized to the standard of care arm are regarded as treated with Sirolimus, if

- They received prophylactic mTOR inhibitor

Screening and disease characteristics will be analysed once by treatment groups as treated.

9.2 Analysis Variables

See section 2.1 and 2.2 for the variables to be analysed as well as section 6.8.

Cognitive impairment will be defined as cognitive scale score <70 and autistic spectrum disorders will be defined as ADOS>12.

A treatment-emergent adverse event is an adverse event that emerges after randomization having been absent pre-randomization, or worsens relative to the pre-randomization state.

9.3 General Considerations

All patient data entered into the eCRF will be listed. Free text will be listed only.
Patient disposition will be analysed based on the CONSORT statement, including number patients screened, patients randomized, patients terminating the study prematurely and the number of patients in the analysis populations.

Screening and disease characteristics will be analyzed descriptively by treatment group for the SAF using standard summary measures, i.e., number of evaluable observations, arithmetic mean, standard deviation, median, 25 %- and 75 %-quantile, minimum and maximum for continuous data and the number and percentage of patients per category for categorical data.

A statistical analysis plan will be written for the main study by the KKS.

All analyses of the main study will be described in the statistical analysis plan in greater detail. Additional analyses may be added. The accompanying research will not be part of this plan.
In case of any changes to the primary analysis described in this protocol an amendment will be issued pointing out the changes. If necessary, all other changes will be mentioned in the SAP, only.

SAS Version 9.4 or higher will be used for analyses.

9.4 Primary Analysis

As **primary analysis**, the BSID-III cognitive scale of 2 years will be analysed using a two-sided Mann-Whitney-U-test (alpha=0.05) stratified by age groups (0-<2 and 2-<4 months of age) comparing the treatment versus the control group. The Mann-Whitney-U-test is used instead of the t-test because there are hints that the values could be not normally distributed in this

population. All patients randomized will be evaluated following the treatment policy strategy (ICH E9 R1).
Missing values of patients (including missing values due to early termination of the study) without a BSID-III cognitive scale at the age of 2 years will be replaced

a) with randomly selected values of patients in the control group (single imputation) if the 1-year value is not available
b) with data of the control patients using a linear regression model with the 1-year value as independent variable and the 2-year value as the dependent variable (single imputation) if the 1-year value is available

Values of the control group will be used in order to achieve a conservative estimate. No fixed value is used for single imputation (e.g. the median) to reduce the risk to underestimate the variance. The seed to select values randomly is 4421360.
Additionally, the median of the BSID-III cognitive scale and the corresponding 95% CI will be displayed by group.

9.5 Secondary Analyses

9.5.1 Main Trial Analyses

All secondary parameters will be analyzed for the FAS.
BSID-III cognitive scale at the age of 1 year will be analysed similar to the primary endpoint. In addition, the BSID-III cognitive scale and important subscales will be analysed descriptively by group and time point using standard descriptive measures for continuous variables. The VABS total scales, important VABS subscales, ADOS total scales, and important ADOS subscales will be analysed descriptively by group and time point using standard descriptive measures for continuous variables, as well. In addition, for cognitive impairment and autism spectrum disorders at 1 and 2 years of age, 95% CIs will be calculated by treatment group in addition to the relative frequencies using the method of Wilson. The M-CHAT-R/F and TAND will be analysed descriptively by group and time point using absolute and relative frequencies.
The MRI characteristics (as defined in Section 6.8) will be analysed by group and time point, i.e. randomization (last image before randomization) and at 2 years of age ± 6 months.
Seizure characteristics, EEG characteristics, abdominal sonography characteristics, ECG and echocardiography characteristics (as defined in Section 6.8) will be analysed by treatment group and time point (if applicable).
All **safety parameters** will be analyzed for the SAF. Only treatment emergent-event adverse events will be analysed. Frequencies (number of adverse events & number and percentage of patients with at least one adverse event) will be tabulated according to system organ class and preferred term. One table each will be created showing: all adverse events, all serious adverse events, all adverse events of special interest, all non-serious adverse events, all adverse events leading to death, all adverse events related to the treatment (treated group only), all adverse events by severity. In addition, adverse events and special situations will be analysed by categories defined in the eCRF (number of events & number and percentage of patients with at least one event).
For laboratory values and vital signs, the analyses will be conducted by visit and shift tables comparing all values by visit to the relevant baseline value will be displayed additionally.

9.5.2 Accompanying Research Analyses

Accompanying research will be conducted by the medical coordinator or his representatives. Analyses will be described separately.

9.6 Interim Analyses

No **interim analyses** regarding efficacy or futility are planned but 15-16 DSMB meetings will take place to assure and evaluate the safety.

9.7 Sensitivity Analyses

The primary analysis will be repeated for the PPS as **sensitivity analysis.**

In addition, the primary analysis will be repeated using a linear mixed model with age groups and treatment as fixed factors and site as random factor. Missing values will be replaced using multiple imputation using age groups and the 1-year value as exploratory variables.

9.8 Subgroup Analyses

The primary analysis will be repeated by age groups (0-<2 and 2-<4 months of age).

The primary analysis will be repeated by the presence of IS at the time of randomization (yes/no).

9.9 Sample Size/Power Calculation

It seems feasible that 60 patients can be randomized. To randomize 60 patients, 120 patients need to be assessed for eligibility assuming 50% willingness to participate in the trial. Assuming that 30 patients per group can be randomized, a difference of 15 in the cognitive scale of BSID-III at age 2 years will be detectable with a power of >95% using a two-sided Mann-Whitney-U-test (alpha=0.05 and under normality assumption with a standard deviation (SD) in each group of 15). We consider an improvement of 15 in the cognitive scale of BSID-III at the age of two years (e.g. a score of 75 in the control group and a score of 90 in the treatment group) relevant for reducing neuropsychological impairment, therefore lifting burden of disease and improving social participation and opportunities in school and professional life. The following Table 8 shows the resulting power for 30 patients per group under different assumptions:

Table 8: Power for 30 patients per group under different assumptions

mean difference	SD per group	power	mean difference considering missing values	SD per group considering missing values	power considering missing values
15	15 (a)	>95%	~ 13.5	~14.7/~15.5	>90%
15	20	>80%	~ 13.5	~19.7/~20.2	>70%
15	25 (b)	>62%	~ 13.5	~24.6/~25.1	>50%
11.5	15 (a)	>80%	~ 10.4	~14.7/~15.2	>70%

a) [15, 99] b) [100]

If the improvement is only 11.5 and assuming a SD in each group of 15 (sample size of 30 per group), the power is still >80%. If the mean difference is 15 and the SD is 25 in each group, the power is reduced to >62%. If the improvement is 11.5 and the SD is 25 in each group, the probability to observe an improvement (difference in medians) >5 is still >75%. An observed improvement (point estimate) of 5 still seems encouraging to proceed, as in this range it can trigger

an individual change from mild intellectual disability to learning disability with implications on social integration, education and professional perspectives.

A rate of missing values of 10% is assumed, in accordance with assumptions made by a comparable trial of mTOR inhibitors in children with TSC [42]. In addition, the EXIST-3 trial, enrolling 366 children and adults with TSC, reported a drop-out rate of 5% [23]. Therefore, a rate of missing values of 10% seems reasonable. The table above (Table 8) shows the reduction of the power, the reduction of the effect and the change of the standard deviation considering 10% missing values and the replacement method. The standard deviation in the control group was slightly reduced, in the treatment group slightly increased on average in the simulations.

Patients with missing values (including missing values due to early termination of the study) will not be excluded from the FAS and the SAF. Therefore, patients won't be replaced.

10. Data Management

For more details refer to the corresponding data management plan (DMP) of this clinical trial. All data management activities will be conducted according to the current SOPs.

10.1 Data Collection and Handling

In this clinical trial a clinical data management system is used for data collection by using an electronic CRF (eCRF) with remote data entry (RDE).

There must be no data that are inconsistent between eCRF and source documents.

In addition, source documents must reflect that the subject has been included in this clinical trial and also include all medical information necessary for appropriate medical care outside of the clinical trial.

All protocol-required information collected during the clinical trial must be entered into the eCRF by the investigator or a designated representative. There must not be subject identifying data in the eCRF. A reason for change has to be entered if a data entry is corrected. Data entry should be completed within 10 working-days after a clinical trial subject participated in an examination, treatment, or any other clinical trial procedures. Any pending data entries have to be completed immediately after the final examination. Explanation should be given for all missing data. The completeness and correctness of all data entries in the eCRF have to be confirmed by dated electronic signatures of the responsible investigator.

The survey instruments for the optional 60-month follow-up study will be mailed (paper-based) directly to study participants and returned to the coordinating study center after they have been completed. In order to ensure the later contact, permission for the storage of personal data (name, address) is requested directly in the consent process.

10.2 Data Cleaning and Quality Checks

Data entries will be checked according to plausibility and consistency. The checks have to be defined in the clinical trial specific data validation plan (DVP). In case of implausibility, 'warnings' will be generated during data entry (edit checks). The responsible investigator or a designated representative will be obliged either to correct the implausible data or to confirm its authenticity and to give appropriate explanation. The responsible data manager will check all explanations and resolve the warnings if the explanation is appropriate. The responsible monitor can generate electronic questions (monitor queries) in the database, that will be send back to the responsible investigator as well. The investigator or a designated representative has to respond preferably within 10 working-days. The responsible monitor will check the query answer and resolve the monitor query if the answer is appropriate. A similar query flow can be used by the data manager (DM query) or Safety Manager (PV Query).

All missing data or inconsistencies have to be clarified by the responsible investigator prior to database lock. If no further corrections in the database are required it will be declared as locked and subsequently used for statistical analysis.

11. Archiving and Storage

11.1 Essential Documents and Source Data

The sponsor and investigator(s) will archive the content of the Trial Master File / Investigator Site File (ISF) as well as source data for at least 25 years after the end of the clinical trial.

These procedures shall include:

- the protocol including rationale, objectives and statistical design and methodology of the clinical trial, with conditions under which it is performed and managed, and details of the investigational product used
- standard operating procedures
- all written opinions on the protocol and procedures
- final report
- Electronic data of the electronic case report forms
- audit certificate(s), if available.
- all other relevant documents of the trial master file, according to the ICH-GCP guideline

Any change of data ownership shall be documented. All data shall be made available to relevant authorities on request.

11.2 Collection, Storage and future Use of Biological Samples and Corresponding Data only in Accompanying Research

Measures are in place to comply with the applicable rules for collection, biobanking and future use of biological samples and clinical data for accompanying research. Sample collection and data usage are in accordance with the separate biobanking informed consent. No biological materials are stored in the main study.

Facilities storing biological samples from clinical trial participants as well as the external banking facility are qualified for the storage of biological samples collected in accompanying research. An appropriate sample and data management system, incl. audit trail for clinical data and samples to identify and destroy such samples according to ICF is in place. A fit for purpose documentation (biomarker proposal, analysis plan and report) ensures compliant usage. Samples and/or data may be transferred to third parties and other countries as specified in the biobanking ICF. Stored samples will be coded throughout the sample storage and analysis process and will not be labelled with personal identifiers. Participants may withdraw their consent for their samples to be stored for research at any time.

12. Regulatory, Ethical and Trial Oversight Considerations

12.1 Compliance Statement

This clinical trial will be conducted in compliance with the protocol and in accordance with the following regulatory requirements:

- Consensus ethical principles derived from international guidelines including the Declaration of Helsinki and Council for International Organizations of Medical Sciences (CIOMS) International Ethical Guidelines
- Applicable ICH GCP Guidelines
- Applicable laws and regulations

Printouts of all applicable legal acts and guidelines will be filed in the trial master file and will be provided to the participating investigators as part of the investigator site file.

12.2 Data Protection and Subject Privacy

The data obtained in the course of the clinical trial will be treated pursuant to the EU General Data Protection Regulation (GDPR, DSGVO) and national regulatory requirements e.g., Bundesdatenschutzgesetz, BDSG, Landesdatenschutzgesetz Baden-Württemberg (LDSG BW)).

To ensure confidentiality of records and personal data, only pseudonymized data will be transferred to the sponsor by using a subject identification number instead of the subject's name. In accordance with §27 of the Bundesdatenschutzgesetz (BDSG), an exemption for the processing of special categories of personal data for circumscribed purposes is possible when the processing is necessary for scientific or statistical purposes (e.g. conduct of the study). The principle of data minimization must be maintained. The consent of the data subjects must be given. The responsible party need to provide for appropriate and specific measures to safeguard the interests of the data subject in accordance with Section 22 (2) Sentence 2. To calculate the age-specific starting dose of the IMP and the exact timing of study visits and neuro psychologic tests, it is necessary to determine the date of birth of study participants.

The subject identification number code is only available at the site and must not be forwarded to the sponsor. In case a subject's records will be forwarded e.g. for SAE processing or adjudication committees, personal data identifying the subject will be redacted by the site prior to forwarding. Access to the subject's files and clinical data is strictly limited: personalized treatment data may be given to the subject's personal physician or to other appropriate medical personnel responsible for the subject's welfare. Trial specific data generated at the site need to be available for inspection on request by the participating physicians, the sponsor's representatives, by the IRB / IEC and the regulatory authorities. A potential data security breach will be assessed regarding the implications for rights and privacy of the affected person(s). Immediate actions as well as corrective and preventive actions will be implemented. Respective regulatory authorities, IRBs / IECs and caregiver(s) of the subjects will be informed as appropriate.

Storage of trial data on a computer will be done in accordance with local data protection law and will be handled in strictest confidence. Distribution of these data to unauthorised persons has to be prevented strictly. The appropriate regulations of local data legislation will be fulfilled in its entirety.
The investigator will maintain a Patient Identification List (Patient IDs with the corresponding patient names) to enable records to be identified. Patients who did not consent to circulate their pseudonymized data will not be included into the trial.
This protocol, the CRFs and other trial-related documents and material must be handled with strict confidentiality and not be disclosed to third parties except with the express prior consent of

Sponsor. In particular, it must be ensured that the study medication is kept out of reach of third parties. Staffs of the investigators involved in this study are also bound by this agreement. Further details are described in local SOPs and study specific manuals, e.g. DMP.

12.3 Trial Approval

This trial will be initiated only after all required legal documentation has been reviewed and approved by the responsible IRB Independent Ethics Committee (IEC) and authorities according to national and international regulations. The same applies for the implementation of changes introduced through amendments.

12.4 Subject Information and Informed Consent

12.4.1 General Provisions

Before being admitted to the clinical trial, the guardian(s) must consent to participate after being fully informed by the investigator or a designated member of the investigating team about the nature, importance, risks and individual consequences of the clinical trial and the guardian(s)'right, to terminate the participation at any time. The guardian(s) should also have the opportunity to consult the investigator, or a physician of the investigating team about the details of the clinical trial. The Investigator will explain that the guardian(s) are completely free to refuse to enter their child to the clinical trial or to withdraw from it at any time, without any consequences for further care of the child and without the need to justify (see section 7.3.1).

Each guardian(s) will be informed that his/her source records may be reviewed by the clinical trial monitor, a quality assurance auditor or authority inspector, in accordance with applicable regulations, and that these persons are bound by confidentiality obligations.

After reading the informed consent document, guardian(s) and physician conducting the informed consent discussion must sign and personally date the informed consent form. A copy of the signed informed consent document must be given to the guardian(s); the original will be filed by the investigator.

Written subject information must be in a language understandable to the guardian(s) and must specify who informed the guardian(s).

The Rapamune® administration process shall be discussed and trained in detail with the caregivers by the trial staff. The caregivers will also receive an information / training leaflet (Additional Information to Rapamune® solution -Handling, Dosage, Administration-) as part of the Informed Consent (Annex II of the IC).

The guardian(s) will be informed as soon as possible if new information may influence his/her/their decision to participate his/her/their child in the clinical trial. The communication of this information should be documented.

12.4.2 Special Provision for Incapacitated Subjects

Not applicable.

12.5 Trial Committee Structure

The following committees are set up to monitor certain aspects of the clinical trial and to ensure expert advice and monitoring independent of the coordinating investigator:

12.5.1 Data Safety Monitoring Board (DSMB)

Ensuring the ethical conduct of the clinical trial and protecting the rights and welfare of the subjects are the tasks of the DSMB.

A DSMB made up of independent experts will be set up. It consists of 2 physicians and a statistician who are not involved in the conduct of the clinical trial. The task of the DSMB is to assess the safety of the clinical trial therapy (e.g. adverse events associated to mTOR inhibitor treatment), and to monitor the integrity and validity of the data collected (e.g. protocol adherence/ protocol deviations) and the conduct of the clinical trial (e.g. recruitment).

The first meeting (kick-off) will be performed **at study start.** Further DSMB-meetings will take place periodically, **in six monthly intervals after first patient in (approximately twice a year, in total 15 DSMB-meetings)** to review the study´s progress, to ensure adherence to the protocol, to advise whether to continue, modify, or stop the study. The DSMB may meet more often if safety concerns arise.

A DSMB-meeting will take place after 5 patients in the treatment group had their fourth visit (titration phase) to evaluate the dosing regarding safety.

For further details see DSMB charter (separate Document), submitted together with the protocol.

12.5.2 Steering Committee (SC)

Not applicable.

12.5.3 Scientific Advisory Board (SAB)

The SAB will consist of 5 independent researchers, who are renowned experts in the therapeutic areas. The SAB will ensure that state-of-the-art scientific standards are met, in particular with regard to the acknowledgement of national guidelines, adherence to trial protocol, implementation of the design, treatments, assessments and statistical analyses, and recommendations for publication of results. Furthermore, they will advise on continuation, modification, conflict resolution and on stopping the trial. In total, 4 SAB-meetings will take place, approximately once a year.

12.6 Insurance

Insurance is taken out for all submitted member states according to Art. 76 of CTR or other national requirements.

13. Quality Control and Quality Assurance

13.1 Quality Assurance

A risk-based approach is used for trial quality management. It is initiated by the assessment of critical data and processes for subject protection and reliability of the results as well as identification and assessment of associated risks. The rationale and strategies for risk management during trial conduct including monitoring approaches, vendor management and other processes focusing on areas of greatest risk will be documented. Continuous risk review and assessment may lead to adjustments in trial conduct, trial design or monitoring approaches. A quality assurance audit/inspection of this trial may be conducted by the sponsor, sponsor's designees, or by IRB / IEC or by regulatory authorities (see section 13.4).

13.2 Monitoring

Following a risk-based approach, the sponsor will use a combination of monitoring techniques (central, remote or on/off-site visits) to monitor the trial.

As the monitoring strategy will consider current aspects of risk-based quality management, frequency of monitoring activities per site will vary depending on recruitment and general performance, e.g. quality of documentation of the individual clinical trial sites.

The monitor will ensure that the clinical trial is conducted according to the protocol and regulatory requirements by review of essential documents, source documents and entries into the eCRFs (see section 13.3 and 13.3.1). The monitor will document the visits in a report for the sponsor. The site will be provided with a follow-up letter of the findings and the necessary actions to be taken.

In addition to on-site monitoring visits, remote contacts can occur. It is expected that during these remote contacts, site personnel will be available to provide an update on the progress of the trial at the site. Central monitoring can take place for data identified by the sponsor. Details of monitoring will be defined in the monitoring plan.

In case of critical findings during monitoring or an audit, the site might be closed prematurely by the sponsor (see section 7.7).

13.3 Source Documents

In accordance with regulatory requirements, the investigator should maintain adequate source documents and trial records including all observations/data pertinent to the investigation on each trial subject. Source data as well as reported data should follow the "ALCOA principles", i.e. should be attributable, legible, contemporaneous, original and accurate. Changes to the data should be traceable (audit trail). All Data reported on the eCRF must be consistent with and verifiable by the source data. Any discrepancies must be explained.

The current medical history of the subject may not be sufficient to confirm eligibility for the trial so that the investigator may need to request records on previous medical history and test results. If required, the investigator must make at least one documented attempt to retrieve previous medical records. If this fails, medical history reported orally by the caregiver, may be acceptable and must be documented in the subject's medical records. In case of incompliance, any corrective action e.g. repeated instructions must be documented in the subject's medical records, too.

13.3.1 Direct Access to Source Documents

According to ICH-GCP the investigator(s)/institution(s) must provide direct access to source data/documents for clinical trial related monitoring, audits and inspections by regulatory authorities. Via the written informed consent, the guardian(s) has/have agreed - to grant trial-

related monitors, auditors and inspectors from regulatory authorities' direct access to the child´s original medical records (see section 12.4.1).

In case of electronic medical records, the monitor's/auditor's/inspector's access must be restricted to the trial subjects. If this is not possible the files have to be reviewed in the presence of site staff. The electronic medical record should have an accessible audit trail.

13.4 Audits and Inspections

Representatives of the sponsor may visit the trial site at any time during or after completion of the trial to audit compliance with applicable regulatory requirements and sponsor policies. Similarly, officials of the responsible authorities may carry out inspections either as part of a national GCP compliance program or to review the trial results in support of a regulatory submission. Both audits and inspections will require access to all trial records and source documents (see section 13.3.1). The investigator and site personnel must be available for consultation during site audits/inspections.

The investigator should immediately notify the sponsor if he/she becomes aware of an upcoming inspection.

13.5 Serious Breaches

If the investigator becomes aware of a potential serious breach as defined by Art. 52 of CTR he/she must inform the sponsor without undue delay, if applicable. Details on the notification process are described in the applicable SOPs.

13.6 Protocol Deviations

Definition, categorization and examples of protocol deviations (PD) are described the in the trial specific PD-plan and the applicable SOPs.

14. Administrative Agreements

14.1 Financing of the Clinical trial

The clinical trial will be financed using funds of BMBF, Pfizer will provide drug (i.e. Sirolimus) free of charge.

14.2 Financial Disclosure

Investigators will provide the sponsor with accurate financial information in accordance with local regulations allowing the sponsor to submit complete and accurate financial certification or disclosure statements to the appropriate regulatory authorities. Investigators are responsible for providing information on financial interests during the conduct of the trial. The investigator agrees to update this information in case of significant changes.

14.3 Publication Policy / Dissemination of Trial Data

A summary of the clinical trial data will be written after all subjects have completed the trial in all countries (EU or non-EU). The sponsor will submit a summary of the final trial results within 6 months (pediatric trial) from the end of a clinical trial. A layperson summary will be provided, too. The rights of the investigator and the sponsor regarding publication of the trial results are described in the investigator contract. In general, no trial results should be published prior to finalization of the Clinical Trial Report.

Publication policy: Consistent with Good Publication Practices and International Committee of Medical Journal Editors (ICMJE) guidelines, the sponsor shall have the right to publish primary (multicenter) data and information without approval from the investigator. The investigator has the right to publish site-specific data after the primary data are published. If an investigator wishes to publish information from the trial, a copy of the manuscript must be provided to the sponsor for review at least 30 days before submission for publication or presentation.

14.4 Declaration regarding Data Sharing

The data from the PROTECT-clinical trial will be archived electronically at University Hospital of Heidelberg. “heiDATA”, a professional data repository provided by the Heidelberg University Competence Centre for Research Data, will be used as prospective data sharing platform. Data will be made available, as far as legally possible. Individual participant data will be available that underlie the results reported in the corresponding article publication after deidentification (text, tables, figures, and appendices, study protocol, statistical analysis plan, analytic code). Data will be available beginning 3 months following article publication with a storage duration depending on server capacity (minimum of 10 years). Data will be shared with researchers who provide a methodologically sound proposal, to achieve aims in the approved proposal. Proposals should be directed to the coordinating investigator or Heidelberg University’s data warehouse (heiDATA, https://heidata.uni-heidelberg.de).

15. References

Appendix 1

1. den Dunnen, J.T., et al., *HGVS Recommendations for the Description of Sequence Variants: 2016 Update.* Hum Mutat, 2016. **37**(6): p. 564-9.
2. Ebrahimi-Fakhari, D., et al., *Incidence of tuberous sclerosis and age at first diagnosis: new data and emerging trends from a national, prospective surveillance study.* Orphanet J Rare Dis, 2018. **13**(1): p. 117.
3. Curatolo, P., R. Moavero, and P.J. de Vries, *Neurological and neuropsychiatric aspects of tuberous sclerosis complex.* Lancet Neurol, 2015. **14**(7): p. 733-45.
4. Mettin, R.R., et al., *Wide spectrum of clinical manifestations in children with tuberous sclerosis complex--follow-up of 20 children.* Brain Dev, 2014. **36**(4): p. 306-14.
5. Zollner, J.P., et al., *A systematic review on the burden of illness in individuals with tuberous sclerosis complex (TSC).* Orphanet J Rare Dis, 2020. **15**(1): p. 23.
6. Crino, P.B., K.L. Nathanson, and E.P. Henske, *The tuberous sclerosis complex.* N Engl J Med, 2006. **355**(13): p. 1345-56.
7. Kingswood, J.C., et al., *TuberOus SClerosis registry to increase disease Awareness (TOSCA) - baseline data on 2093 patients.* Orphanet J Rare Dis, 2017. **12**(1): p. 2.
8. Capal, J.K., et al., *Influence of seizures on early development in tuberous sclerosis complex.* Epilepsy Behav, 2017. **70**(Pt A): p. 245-252.
9. Lipton, J.O. and M. Sahin, *The neurology of mTOR.* Neuron, 2014. **84**(2): p. 275-91.
10. de Vries, P.J. and C.J. Howe, *The tuberous sclerosis complex proteins--a GRIPP on cognition and neurodevelopment.* Trends Mol Med, 2007. **13**(8): p. 319-26.
11. Capal, J.K. and D.N. Franz, *Profile of everolimus in the treatment of tuberous sclerosis complex: an evidence-based review of its place in therapy.* Neuropsychiatr Dis Treat, 2016. **12**: p. 2165-72.
12. Hwang, S.K., et al., *Everolimus improves neuropsychiatric symptoms in a patient with tuberous sclerosis carrying a novel TSC2 mutation.* Mol Brain, 2016. **9**(1): p. 56.
13. Samueli, S., et al., *Everolimus in infants with tuberous sclerosis complex-related West syndrome: First results from a single-center prospective observational study.* Epilepsia, 2018. **59**(9): p. e142-e146.
14. Kotulska, K., et al., *Prevention of Epilepsy in Infants with Tuberous Sclerosis Complex in the EPISTOP Trial.* Ann Neurol, 2021. **89**(2): p. 304-314.
15. Moavero, R., et al., *Early Clinical Predictors of Autism Spectrum Disorder in Infants with Tuberous Sclerosis Complex: Results from the EPISTOP Study.* J Clin Med, 2019. **8**(6).
16. de Vries, P.J., et al., *TSC-associated neuropsychiatric disorders (TAND): findings from the TOSCA natural history study.* Orphanet J Rare Dis, 2018. **13**(1): p. 157.
17. Joinson, C., et al., *Learning disability and epilepsy in an epidemiological sample of individuals with tuberous sclerosis complex.* Psychol Med, 2003. **33**(2): p. 335-44.
18. Chu-Shore, C.J., et al., *The natural history of epilepsy in tuberous sclerosis complex.* Epilepsia, 2010. **51**(7): p. 1236-41.
19. Bolton, P.F., et al., *Neuro-epileptic determinants of autism spectrum disorders in tuberous sclerosis complex.* Brain, 2002. **125**(Pt 6): p. 1247-55.
20. Bombardieri, R., et al., *Early control of seizures improves long-term outcome in children with tuberous sclerosis complex.* Eur J Paediatr Neurol, 2010. **14**(2): p. 146-9.
21. Jozwiak, S., et al., *Preventive Antiepileptic Treatment in Tuberous Sclerosis Complex: A Long-Term, Prospective Trial.* Pediatr Neurol, 2019. **101**: p. 18-25.
22. Krueger, D.A., et al., *Everolimus treatment of refractory epilepsy in tuberous sclerosis complex.* Ann Neurol, 2013. **74**(5): p. 679-87.

23. French, J.A., et al., *Adjunctive everolimus therapy for treatment-resistant focal-onset seizures associated with tuberous sclerosis (EXIST-3): a phase 3, randomised, double-blind, placebo-controlled study.* Lancet, 2016. **388**(10056): p. 2153-2163.
24. Tsai, P.T., et al., *Autistic-like behaviour and cerebellar dysfunction in Purkinje cell Tsc1 mutant mice.* Nature, 2012. **488**(7413): p. 647-51.
25. Schneider, M., et al., *mTOR inhibitor reverses autistic-like social deficit behaviours in adult rats with both Tsc2 haploinsufficiency and developmental status epilepticus.* Eur Arch Psychiatry Clin Neurosci, 2017. **267**(5): p. 455-463.
26. Ehninger, D., et al., *Reversal of learning deficits in a Tsc2+/- mouse model of tuberous sclerosis.* Nat Med, 2008. **14**(8): p. 843-8.
27. Krueger, D.A., et al., *Short-term safety of mTOR inhibitors in infants and very young children with tuberous sclerosis complex (TSC): Multicentre clinical experience.* Eur J Paediatr Neurol, 2018.
28. Saffari, A., et al., *Safety and efficacy of mTOR inhibitor treatment in patients with tuberous sclerosis complex under 2 years of age - a multicenter retrospective study.* Orphanet J Rare Dis, 2019. **14**(1): p. 96.
29. Novartis. *A post authorization safety study to evaluate the long-term safety of Votubia® in patients with Tuberous Sclerosis Complex participating in the TOSCA Disease Registry*. 2020 14.08.2023]; Available from: https://www.encepp.eu/encepp/openAttachment/studyResult/36192.
30. Davis, P.E., et al., *Presentation and Diagnosis of Tuberous Sclerosis Complex in Infants.* Pediatrics, 2017. **140**(6).
31. Sahin, M., et al., *Advances and Future Directions for Tuberous Sclerosis Complex Research: Recommendations From the 2015 Strategic Planning Conference* Pediatr Neurol, 2016. **60**.
32. Rössler, J., et al., *Severe adverse events during sirolimus “off-label” therapy for vascular anomalies.* Pediatric Blood and Cancer, 2021. **68**(8).
33. Wang, Z., Yao, W., Sun, H., Dong, K., Ma, Y., Chen, L., Zheng, S., Li, K., *Sirolimus therapy for kaposiform hemangioendothelioma with long-term follow-up.* Dermatology, 2019. **46**(11): p. 956-961.
34. Shaw R, H.A., Luu T, O'Connor TE, Hamidi A, Fitzsimons J, Varda B, Kwon D, Whitcomb C, Gregorowicz A, Roloff GW, Bemiss BC, Kallwitz ER, Hagen PA, Berg S., *Multicenter analysis of immunosuppressive medications on the risk of malignancy following adult solid organ transplantation. .* Front Oncol, 2023. **13**.
35. Sasongko TH, K.K., Chai Soon Hou S, Jocelyn TXY, Zabidi-Hussin Z. , *Rapamycin and rapalogs for tuberous sclerosis complex.* Cochrane Database Syst Rev., 2023. **11**(7).
36. He, W., et al., *Sirolimus improves seizure control in pediatric patients with tuberous sclerosis: A prospective cohort study.* Seizure, 2020. **79**: p. 20-26.
37. *Ethical considerations for clinical trials on medicinal products conducted with minors*. 2017; Available from: https://health.ec.europa.eu/system/files/2018-02/2017_09_18_ethical_consid_ct_with_minors_0.pdf.
38. Lebwohl, D., et al., *Research and innovation in the development of everolimus for oncology.* Expert Opin Drug Discov, 2011. **6**(3): p. 323-38.
39. Bissler, J.J., et al., *Sirolimus for angiomyolipoma in tuberous sclerosis complex or lymphangioleiomyomatosis.* N Engl J Med, 2008. **358**(2): p. 140-51.
40. MacKeigan, J.P. and D.A. Krueger, *Differentiating the mTOR inhibitors everolimus and sirolimus in the treatment of tuberous sclerosis complex.* Neuro Oncol, 2015. **17**(12): p. 1550-9.
41. Franz, D.N. and D.A. Krueger, *mTOR inhibitor therapy as a disease modifying therapy for tuberous sclerosis complex.* Am J Med Genet C Semin Med Genet, 2018. **178**(3): p. 365-373.

42. Overwater, I.E., et al., *Sirolimus for epilepsy in children with tuberous sclerosis complex: A randomized controlled trial.* Neurology, 2016. **87**(10): p. 1011-8.
43. Moher, D., et al., *Preferred reporting items for systematic reviews and meta-analyses: the PRISMA statement.* J Clin Epidemiol, 2009. **62**(10): p. 1006-12.
44. Bevacqua, M., et al., *Off-Label Use of Sirolimus and Everolimus in a Pediatric Center: A Case Series and Review of the Literature.* Paediatr Drugs, 2019. **21**(3): p. 185-193.
45. Śmiałek, D., S. Jóźwiak, and K. Kotulska, *Safety of Sirolimus in Patients with Tuberous Sclerosis Complex under Two Years of Age—A Bicenter Retrospective Study.* Journal of Clinical Medicine, 2023. **12**(1).
46. Maria, G., et al., *Sirolimus: Efficacy and Complications in Children With Hyperinsulinemic Hypoglycemia: A 5-Year Follow-Up Study.* J Endocr Soc, 2019. **3**(4): p. 699-713.
47. Knoll, G.A., et al., *Effect of sirolimus on malignancy and survival after kidney transplantation: systematic review and meta-analysis of individual patient data.* BMJ, 2014. **349**: p. g6679.
48. Briem-Richter, A., et al., *Linear growth and sexual maturation with everolimus plus reduced calcineurin inhibitorbased regimen in pediatric liver transplant recipients: 12-month results from H2305 study.* Pediatric Transplantation, 2017. **21**: p. 13-14.
49. Gonzalez, D., et al., *Growth of kidney-transplanted pediatric patients treated with sirolimus.* Pediatr Nephrol, 2011. **26**(6): p. 961-6.
50. Hymes, L.C. and B.L. Warshaw, *Linear growth in pediatric renal transplant recipients receiving sirolimus.* Pediatr Transplant, 2011. **15**(6): p. 570-2.
51. Bischof, E., et al., *The potential of rapalogs to enhance resilience against SARS-CoV-2 infection and reduce the severity of COVID-19.* Lancet Healthy Longev, 2021. **2**(2): p. e105-e111.
52. Ihnen, S.K.Z., et al., *Epilepsy Is Heterogeneous in Early-Life Tuberous Sclerosis Complex.* Pediatr Neurol, 2021. **123**: p. 1-9.
53. Martin, K.R., et al., *The genomic landscape of tuberous sclerosis complex.* Nat Commun, 2017. **8**: p. 15816.
54. Au, K.S., C.H. Ward, and H. Northrup, *Tuberous sclerosis complex: disease modifiers and treatments.* Curr Opin Pediatr, 2008. **20**(6): p. 628-33.
55. Mannick, J.B., et al., *mTOR inhibition improves immune function in the elderly.* Sci Transl Med, 2014. **6**(268): p. 268ra179.
56. Mannick, J.B., et al., *TORC1 inhibition enhances immune function and reduces infections in the elderly.* Sci Transl Med, 2018. **10**(449).
57. de Vries, P.J., et al., *Measuring health-related quality of life in Tuberous sclerosis complex - psychometric evaluation of three instruments in individuals with refractory epilepsy.* Frontiers in Pharmacology, 2018. **9**(AUG).
58. Northrup, H., et al., *Updated International Tuberous Sclerosis Complex Diagnostic Criteria and Surveillance and Management Recommendations.* Pediatr Neurol, 2021. **123**: p. 50-66.
59. Pfizer, *INVESTIGATOR’S BROCHURE Sirolimus (PF-00835135)*. 2015, Pfizer GmbH.
60. Wu, C.Y. and L.Z. Benet, *Predicting drug disposition via application of BCS: transport/absorption/ elimination interplay and development of a biopharmaceutics drug disposition classification system.* Pharm Res, 2005. **22**(1): p. 11-23.
61. Mizuno, T., M.M. O'Brien, and A.A. Vinks, *Significant effect of infection and food intake on sirolimus pharmacokinetics and exposure in pediatric patients with acute lymphoblastic leukemia.* European Journal of Pharmaceutical Sciences, 2019. **128**: p. 209-214.
62. Marek, E. and W.K. Kraft, *Ethanol pharmacokinetics in neonates and infants.* Curr Ther Res Clin Exp, 2014. **76**: p. 90-7.
63. Lim, T.Y., R.L. Poole, and N.M. Pageler, *Propylene glycol toxicity in children.* J Pediatr Pharmacol Ther, 2014. **19**(4): p. 277-82.

64. Pandya, H.C., et al., *Essential medicines containing ethanol elevate blood acetaldehyde concentrations in neonates.* Eur J Pediatr, 2016. **175**(6): p. 841-7.
65. Tran, M.N., A.H. Wu, and D.W. Hill, *Alcohol dehydrogenase and catalase content in perinatal infant and adult livers: potential influence on neonatal alcohol metabolism.* Toxicol Lett, 2007. **169**(3): p. 245-52.
66. Whittaker, A., et al., *Toxic additives in medication for preterm infants.* Arch Dis Child Fetal Neonatal Ed, 2009. **94**(4): p. F236-40.
67. De Cock, R.F., et al., *Developmental pharmacokinetics of propylene glycol in preterm and term neonates.* Br J Clin Pharmacol, 2013. **75**(1): p. 162-71.
68. Yu, D.K., W.F. Elmquist, and R.J. Sawchuk, *Pharmacokinetics of propylene glycol in humans during multiple dosing regimens.* J Pharm Sci, 1985. **74**(8): p. 876-9.
69. Shehab, N., et al., *Exposure to the pharmaceutical excipients benzyl alcohol and propylene glycol among critically ill neonates.* Pediatr Crit Care Med, 2009. **10**(2): p. 256-9.
70. Pikkarainen, P.H. and N.C. Raiha, *Development of alcohol dehydrogenase activity in the human liver.* Pediatr Res, 1967. **1**(3): p. 165-8.
71. Allegaert, K., et al., *Prospective assessment of short-term propylene glycol tolerance in neonates.* Arch Dis Child, 2010. **95**(12): p. 1054-8.
72. Chicella, M., et al., *Propylene glycol accumulation associated with continuous infusion of lorazepam in pediatric intensive care patients.* Crit Care Med, 2002. **30**(12): p. 2752-6.
73. Akinmboni, T.O., et al., *Excipient exposure in very low birth weight preterm neonates.* J Perinatol, 2018. **38**(2): p. 169-174.
74. Barnes, B.J., et al., *Osmol gap as a surrogate marker for serum propylene glycol concentrations in patients receiving lorazepam for sedation.* Pharmacotherapy, 2006. **26**(1): p. 23-33.
75. **O'Callaghan FJK, J.K.S.W.E., Fabienne Dietrich Alber et al.** , ***Vigabatrin with hormonal treatment versus hormonal treatment alone (ICISS) for infantile spasms: 18-month outcomes of an open-label, randomised controlled trial.*** . Lancet Child Adolesc Health, 2018. **2**(10): p. 715-725.
76. Farwell, J.R., et al., *Phenobarbital for febrile seizures--effects on intelligence and on seizure recurrence.* N Engl J Med, 1990. **322**(6): p. 364-9.
77. Gesellschaft_für_Neuropädiatrie. *Therapie der Blitz-Nick-Salaam Epilepsie (West-Syndrom), aktualisierte Version 3.0.* 2021 02/2021 [cited 2021 22.11.2022]; Available from: https://www.awmf.org/leitlinien/detail/ll/022-022.html
78. Prevey, M.e.a. *Effect of valproate on cognitive functioning. Comparison with carbamazepine.* . 1996.
79. Mizuno, T., et al., *Model-based precision dosing of sirolimus in pediatric patients with vascular anomalies.* Eur J Pharm Sci, 2017. **109S**: p. S124-S131.
80. Emoto, C., et al., *Characterizing the Developmental Trajectory of Sirolimus Clearance in Neonates and Infants.* CPT Pharmacometrics Syst Pharmacol, 2016. **5**(8): p. 411-7.
81. Jacobsen, W., et al., *Comparison of the in vitro metabolism of the macrolide immunosuppressants sirolimus and RAD.* Transplant Proc, 2001. **33**(1-2): p. 514-5.
82. Emoto, C., et al., *Development of a Physiologically-Based Pharmacokinetic Model for Sirolimus: Predicting Bioavailability Based on Intestinal CYP3A Content.* CPT Pharmacometrics Syst Pharmacol, 2013. **2**(7): p. e59.
83. Hines, R.N. and D.G. McCarver, *The ontogeny of human drug-metabolizing enzymes: phase I oxidative enzymes.* J Pharmacol Exp Ther, 2002. **300**(2): p. 355-60.
84. Stevens, J.C., et al., *Developmental expression of the major human hepatic CYP3A enzymes.* J Pharmacol Exp Ther, 2003. **307**(2): p. 573-82.
85. Wang, D.D., et al., *Initial Dosage Recommendation for Sirolimus in Children With Tuberous Sclerosis Complex.* Front Pharmacol, 2020. **11**: p. 890.

86. Kearns, G.L., et al., *Developmental pharmacology--drug disposition, action, and therapy in infants and children.* N Engl J Med, 2003. **349**(12): p. 1157-67.
87. de Wildt, S.N., et al., *Cytochrome P450 3A: ontogeny and drug disposition.* Clin Pharmacokinet, 1999. **37**(6): p. 485-505.
88. Strolin Benedetti, M., R. Whomsley, and E.L. Baltes, *Differences in absorption, distribution, metabolism and excretion of xenobiotics between the paediatric and adult populations.* Expert Opin Drug Metab Toxicol, 2005. **1**(3): p. 447-71.
89. Mizuno, T., et al., *Developmental pharmacokinetics of sirolimus: Implications for precision dosing in neonates and infants with complicated vascular anomalies.* Pediatr Blood Cancer, 2017. **64**(8).
90. Chen, X., et al., *Population Pharmacokinetics and Initial Dose Optimization of Sirolimus Improving Drug Blood Level for Seizure Control in Pediatric Patients With Tuberous Sclerosis Complex.* Front Pharmacol, 2021. **12**: p. 647232.
91. Gehan, E.A. and S.L. George, *Estimation of human body surface area from height and weight.* Cancer Chemother Rep, 1970. **54**(4): p. 225-35.
92. Franz, D.N., et al., *Everolimus dosing recommendations for tuberous sclerosis complex-associated refractory seizures.* Epilepsia, 2018. **59**(6): p. 1188-1197.
93. *Rapamune - EPAR - Product Information, ANNEX I, SUMMARY OF PRODUCT CHARACTERISTICS*. [Product Information] 2022 25.07.2022 [cited 2023 28.02.]; Available from: https://www.ema.europa.eu/en/medicines/human/EPAR/rapamune.
94. Zhang, J., et al., *Effect of CYP3A4 and CYP3A5 Genetic Polymorphisms on the Pharmacokinetics of Sirolimus in Healthy Chinese Volunteers.* Ther Drug Monit, 2017. **39**(4): p. 406-411.
95. Neuropädiatrie, G.f. *Therapie der Blitz-Nick-Salaam Epilepsie (West-Syndrom), aktualisierte Version 3.0.* 2021 02/2021 [cited 2021 22.11.2022]; Available from: https://www.awmf.org/leitlinien/detail/ll/022-022.html
96. Michaelis, R., et al., *Validierte und teilvalidierte Grenzsteine der Entwicklung.* Monatsschrift Kinderheilkunde, 2013. **161**(10): p. 898-910.
97. *Entwicklungsneurologie und Neuropädiatrie*, R. Michaelis and G. Niemann, Editors. 2010, Georg Thieme Verlag KG: Stuttgart.
98. Robins, D.L., et al., *Validation of the modified checklist for Autism in toddlers, revised with follow-up (M-CHAT-R/F).* Pediatrics, 2014. **133**(1): p. 37-45.
99. Bayley, N., *Bayley Scales of Infant and Toddler Development: Bayley-III*. 3 ed. 2014, Frankfurt am Main: Pearson.
100. Bolton, P.F., et al., *Intellectual abilities in tuberous sclerosis complex: risk factors and correlates from the Tuberous Sclerosis 2000 Study.* Psychol Med, 2015. **45**(11): p. 2321-31.

16. Appendices

16.1 Appendix A

16.1.1 Patient Involvement: Letter of support of TSD (Tuberöse Sklerose Deutschland e.V.)

Tuberöse Sklerose Deutschland e. V.
www.tsdev.org

[redacted]
Sektion pädiatrische Epileptologie
Klinik für Kinderheilkunde
Universitätsklinikum Heidelberg
Im Neuenheimer Feld 430
69120 Heidelberg

Wiesbaden, 22. Mai 2020

Als Anlage zur Projektskizze
"Long-term neuropsychologic outcome of pre-emptive mTOR inhibitor treatment in children with tuberous sclerosis complex (TSC) under 4 months of age (PROTECT)"

Studienvorhaben zum prophylaktischen Einsatz von mTOR-Inhibitoren bei Kindern mit Tuberöse Sklerose Complex (TSC)

Sehr geehrte Damen und Herren,

als Patienten- und Elternvereinigung von Menschen mit tuberöser Sklerose unterstützen wir das vorgeschlagene Forschungsvorhaben, da es eine aktuelle und für die Patienten und deren langfristige Entwicklungschancen hochwichtige Frage adressiert.

Die Entwicklungschancen und die Einflussfaktoren auf die frühkindliche Entwicklung bei tuberöser Sklerose sind ein gerade für die betroffenen Familien wichtiges Thema und der frühe Einsatz von mTOR-Inhibitoren, wie Sirolimus, ist für Eltern eine sehr relevante Frage. Da es bisher keine verlässlichen Daten über den Nutzen oder Schaden eines sehr frühen Einsatzes dieser Medikamentengruppe auf die langfristige Entwicklung und die langfristigen Krankheitssymptome gibt, halten wir das Forschungsvorhaben von Herrn Dr. Steffen Syrbe und Herrn Dr. Afshin Saffari aus der Unikinderklinik Heidelberg für einen wichtigen Beitrag, der langfristig die Versorgung von Kindern mit tuberöser Sklerose stark verbessern kann.

Wir freuen uns, das Studienvorhaben durch unsere Struktur der 19 deutschen Tuberöse Sklerose Zentren und durch den Kontakt zu mehr als 1.000 Betroffenen unterstützen zu dürfen.

Mit freundlichen Grüßen

[redacted]

Helmut Hehn
Bundesvorsitzender

[redacted]

16.1.2 Patient Involvement: Letter of support of Achse e.V (Alliance of Chronic Rare Diseases (“Allianz Chronischer Seltener Erkrankungen“))

achse
Allianz Chronischer Seltener Erkrankungen

ACHSE e.V. c/o DRK [illegible] Mitte, Drontheimer Str. 39, [illegible] Berlin

Herrn
PD Dr. Steffen Syrbe
Universitätsklinikum Heidelberg
Sektion pädiatrische Eptileptologie
Im Neuenheimer Feld 672
69120 Heidelberg

[redacted]

Berlin, 24.01.2021

Letter of support for the confirmatory clinical trial study **PROTECT** for treatment in children with Tuberous Sclerosis Complex (TSC)

To whom it may concern

There are an estimated 4 million people living with Rare Disease in Germany. The German Alliance for Rare Diseases (ACHSE) e.V. has been founded in 2004 to increase the awareness of the needs and problems of people living with a Rare Disease and to contribute to the improvement of the living conditions of those affected and their families. Presently more than 130 patient organizations have joined the ACHSE to support each other, to exchange know-how and to strengthen their influence in the political arena. ACHSE voices the demands of patients living with Rare diseases.

As an umbrella organization, ACHSE does not work on a disease-specific basis. This is reserved for our member organizations, which offer this specific disease-related knowledge. However, in our exchange with parents of affected children we experience again and again how laborious, costly and physically as well as psychologically exhausting the care of young patients can be, especially with neurological/neuropsychological developmental disorders. And that there is so much hope associated with therapeutic approaches that can alleviate the symptoms of the disease and improve the developmental chances of those affected. Significant in this context - and this applies almost ubiquitously to the field of rare diseases - is the high willingness of parents of affected persons, but also of affected persons who can speak for themselves, to engage in therapeutic research

approaches, not only to contribute with biomaterial, but to be actively involved in the development of a project idea in terms of content, to be a multiplier in order to recruit study participants, but also to disseminate project results. A trustful, close collaboration that takes into account the strengths of the different participating parties will have a positive impact on the outcome of the study!

In the underlying project, this form of collaboration is met in many ways: through early involvement of Tuberous Sclerosis Germany e.V. into developing the project idea and design of the study, through the use of patient surveys in the course of the project and the dissemination of study results at the end.

ACHSE appreciates and supports efforts that aim to improve the quality of life for people living with Rare Diseases. We therefore hope that the application the clinical study "PROTECT" will be successful.

With best regards

[redacted]

Stellvertretende Geschäftsführerin ACHSE
ACHSE Lotsin für Ärzte und Therapeuten/
Leiterin Wissensnetzwerk und Beratung
